# Formalized peer referral to HIV pre-exposure prophylaxis supported with self-testing: a mixed-methods pilot study among young Kenyan women

**DOI:** 10.3389/fpubh.2024.1428609

**Published:** 2024-09-11

**Authors:** Maureen McGowan, Njeri Wairimu, Adriana M. Reedy, Peter Mogere, Carlos Culquichicon, Irene Njeru, Rachel C. Malen, Albrecht Jahn, Till Bärnighausen, Stephanie D. Roche, Kenneth Ngure, Katrina F. Ortblad

**Affiliations:** ^1^Heidelberg Institute of Global Health, Heidelberg University, Heidelberg, Germany; ^2^School for Data Science and Computational Thinking, Stellenbosch University, Stellenbosch, South Africa; ^3^Partners in Health and Research Development, Center for Clinical Research, Kenya Medical Research Institute, Nairobi, Kenya; ^4^Public Health Sciences Division, Fred Hutchinson Cancer Center, Seattle, WA, United States; ^5^Department of Epidemiology, University of Washington, Seattle, WA, United States; ^6^Harvard Center for Population and Development Studies, Cambridge, MA, United States; ^7^Africa Health Research Institute, Durban, KwaZulu-Natal, South Africa; ^8^School of Public Health, Jomo Kenyatta University of Agriculture and Technology, Nairobi, Kenya; ^9^Department of Global Health, University of Washington, Seattle, WA, United States

**Keywords:** HIV self-test, pre-exposure prophylaxis, peer delivery, AGYW, sub-Saharan Africa

## Abstract

**Background:**

The uptake of daily oral HIV pre-exposure prophylaxis (PrEP)—a highly effective intervention—remains low among African adolescent girls and young women (AGYW) who could benefit. AGYW who initiate PrEP often do so through informal peer referral, which may be enhanced with formalized peer referral and peer-delivered HIV self-testing (HIVST). To understand the feasibility of this PrEP referral model among AGYW, we conducted a pilot study in Kenya.

**Method:**

From March to May 2022, we recruited AGYW (≥16–24 years) using PrEP (i.e., “peer providers”) from public healthcare clinics in Kiambu County and trained them on HIV prevention, HIVST use, and peer-supported linkage to clinic-based HIV services. Following training, peer providers received eight HIVST kits and were encouraged to refer four peers (i.e., “peer clients”) to PrEP. We completed surveys with peer providers and clients one month following intervention delivery to assess PrEP initiation among peer clients. Later, we conducted focus group discussions (FGDs) with peer providers and clients to identify factors that facilitated or challenged intervention outcomes.

**Results:**

We trained 16 peer providers (median age: 23 years, IQR 21–24) who reported delivering the intervention to 56 peer clients; 30 peer clients (median age: 21 years, IQR 19–22) contacted the study team and were enrolled. Most of the enrolled peer clients reported behaviors associated with HIV risk (e.g., condomless sex; 80%, 24/30) and were PrEP-naïve (87%, 26/30). At one-month, PrEP initiation among eligible PrEP-naïve peer clients was high, as reported by providers (78%, 43/55) and clients (85%, 22/26); recent HIVST use was also high among peer clients (provider report: 95%, 53/56; client report: 97%, 29/30). In the FGDs, participants reported that intervention outcomes were facilitated by close preexisting relationships, HIVST assistance, and being escorted to clinic-based HIV services by peer providers; intervention barriers included conflicting priorities and limited HIVST experience.

**Conclusion:**

A formalized model of peer referral with HIVST delivery supported PrEP initiation among Kenyan AGYW. These findings demonstrate the potential for peer-delivered interventions to engage AGYW in HIV prevention services; however, more research is needed on the effectiveness and sustainability of this approach at scale.

## 1 Introduction

Free HIV testing and HIV pre-exposure prophylaxis (PrEP) services are widely available at public healthcare clinics in Kenya (1, 2), yet PrEP uptake remains low among adolescent girls and young women (AGYW) (3, 4). In sub-Saharan Africa, AGYW 16–24 years are at disproportionately high risk of acquiring HIV infection (5, 6). According to 2020 estimates, Kenyan AGYW accounted for roughly twice as many new HIV infections as their male counterparts (2, 6) due to factors such as: limited sex education, low perception of HIV risk, and physical and socioeconomic barriers to healthcare access (e.g., healthcare provider stigma toward unmarried sexually active AGYW) (6–12). Considering these circumstances, several African countries have identified AGYW as a priority population for the delivery of HIV prevention interventions, including PrEP (13).

Peer-delivered HIV prevention interventions have worked well for populations with tight social connectivity (e.g., men who have sex with men (14, 15), female sex workers (16–18)) but have not been widely implemented among AGYW (19, 20); research suggest that AGYW often initiate HIV prevention services through informal (i.e., word-of-mouth) peer referral (21, 22). Peers may help AGYW access HIV services by providing economic and/or emotional support (20, 23–25). For example, AGYW may feel more comfortable asking peers questions about HIV prevention than healthcare providers (20). To leverage the trust AGYW have with their peers and further engage them in HIV prevention services, we developed a formalized model of peer referral to clinic-based PrEP services that includes AGYW-appropriate HIV education and strategies for peer referral. In this model, we additionally included peer delivery of HIV self-testing (HIVST) kits based on evidence that HIVST increases recent HIV testing in other populations with tight social connectivity (16, 26, 27) and that confirmation of an HIV-negative status may motivate HIV prevention behaviors (28–30); a paradigm shift from most other HIVST interventions that have focused on identifying new individuals living with HIV and linking them to HIV treatment services (31–34).

Our formalized model of peer PrEP referral supported by HIVST delivery for AGYW was informed by formative qualitative research (22) and engagement with key Kenyan stakeholders (35). From interviews with PrEP-experienced and PrEP-naïve Kenyan AGYW, we found that many would be willing to engage in such an intervention if delivered by a peer with whom they had a close relationship and who expressed genuine concern about their wellbeing (22). The model was subsequently refined based on feedback from Kenyan PrEP stakeholders—including representatives from the Kenyan Ministry of Health, PrEP implementing organizations, and AGYW advocacy groups—during a formal one-day meeting. Here, the core components of the model (i.e., recruitment, training, HIVST use, linkage to PrEP) and implementation strategies were discussed to increase the model’s potential feasibility and effectiveness (35). In this study, we pilot tested the refined peer PrEP referral + HIVST delivery model to understand the model’s acceptability, appropriateness, and feasibility among Kenyan AGYW and identify model weak points for refinement.

## 2 Methods

### 2.1 Study design and setting

The “Peer PrEP Pilot” study (NCT04982250)^1^ was a single-arm, prospective pilot study that used a sequential explanatory mixed-methods design; first we collected quantitative data then collected qualitative data to explain our quantitative findings (36, 37). The pilot was conducted in Kiambu County, Kenya; a peri-urban region in central Kenya with a population-level HIV prevalence of 2.1% (38).

### 2.2 Participants

The Peer PrEP Pilot had two types of participants: (1) “peer providers,” AGYW who were using PrEP and trained to refer peers to PrEP services, and (2) “peer clients,” AGYW who were not using PrEP and were referred to PrEP services by peer providers. Eligible peer providers were ≥ 16–24 years old, had been using PrEP for at least 3 months (self-reported), and could identify at least four peers whom they thought might benefit from PrEP; eligible peer clients were ≥ 16–24 years old and received the intervention from a peer provider. All participants 16 or 17 years old were emancipated minors (e.g., a parent, head of household, pregnant), per Kenya’s research guidelines (39). Our target sample size was 16 peer providers and up to 64 peer clients, which we determined sufficient for providing preliminary evidence on the intervention that could inform a larger cluster-randomized controlled trial (40).

### 2.3 Recruitment

To recruit peer providers, we contacted AGYW who were engaged in PrEP services at public healthcare clinics, had prior experience participating in PrEP research studies, or were engaged in HIV prevention programs ongoing in the region—such as the Determined, Resilient, Empowered, AIDS-free, Mentored, Safe (DREAMS) program (a comprehensive sexual and reproductive health program tailored to AGYW to reduce risk of HIV (41)). All peer clients were recruited by peer providers. For peer clients to enroll in the study, they needed to call the study line, which was shared with them by a peer provider at the point of recruitment. If they did not have money to call the study line, they could “flash” it (i.e., call and hang up) to receive a call back from the study team. Those who contacted the study line were invited to the Partners in Health and Research Development (PHRD) research site one month later to validate eligibility and enrollment.

### 2.4 Peer provider training

A total of 16 peer providers were recruited and trained in groups of four to seven on the core components of the intervention. This one-day training was held at the PHRD research site and led by female Kenyan researchers who were experienced with PrEP delivery and AGYW engagement. The training curriculum and activities were adapted by the study staff from previous HIV prevention studies conducted among AGYW. The training included presentations on PrEP use and safety, HIVST use, strategies for engaging peers in conversations about HIV risk and prevention, and supporting peer client referral to nearby public healthcare clinics for free HIV prevention or treatment services. Presentation modules were accompanied by pre-and post-training surveys, AGYW-appropriate activities (e.g., a BINGO game), peer recruitment role-playing, and an opportunity to practice HIV self-testing.

### 2.5 Intervention: formalized peer PrEP referral with HIVST delivery

The intervention, delivered by peer providers to peer clients, included information on PrEP for HIV prevention and settings where PrEP services were freely available, as well as HIVST kits for determination of HIV status. Following training completion, peer providers were instructed to deliver the intervention to four peer clients between ≥16 and 24 years whom they thought might benefit from PrEP, ideally in private locations. To facilitate intervention delivery, peer providers were given a recruitment script (in English and Kiswahili, which they practiced during training) (*n* = 1), educational brochures with information on PrEP and HIVST (*n* = 4), printed lists with the information of nearby public healthcare clinics offering free PrEP and antiretroviral therapy (ART) services (*n* = 4), and HIVST kits (*n* = 8). Each recruited peer client was to receive one brochure, two HIVST kits (one for personal use and another for a sexual partner or repeat testing), and one list of nearby clinics.

At the point of intervention delivery, peer providers could disclose their PrEP use to peer clients and assist with HIVST at their discretion and that of their peer clients. Additionally, peer providers could accompany their peer clients to nearby public healthcare clinics for linkage to HIV services, if that seemed appropriate to both parties. For this study, we used the SURE CHECK HIV 1/2 Assay HIVST kit (ChemBio Diagnostic Systems Inc., NY, USA), a blood-based HIVST kit with 99.7% sensitivity and 99.9% specificity that presents results in 15 minutes (42).

### 2.6 Quantitative data collection

Peer providers completed two quantitative surveys, at enrollment (following training) and at follow-up one month later. Peer clients completed one quantitative survey one month after recruitment (at their enrollment). All surveys included information on participants’ demographics (e.g., age, income, education) and behaviors associated with HIV risk -- assessed using a modified version of Kenya’s eight-item Rapid Assessment Screening Tool (RAST) (43). The follow-up surveys also included information about what intervention tools peer providers delivered and peer clients received, and whether peer clients used the HIVST kit and initiated any clinic-based HIV services (i.e., PrEP or ART). Peer providers answered questions about the peer clients they recruited to the best of their knowledge; an unknown response category was available for all peer client outcomes reported by peer providers.

Additionally, the surveys assessed participants’ perceptions of the intervention’s acceptability, appropriateness, and feasibility using validated scales. To inform our acceptability assessment, we used seven statements (with 5-point Likert scales) that assessed different constructs (e.g., affective attitude, burden) of the Theoretical Framework of Acceptability (TFA) (44, 45). To inform our appropriateness and feasibility assessments, we used two statements (with 5-point Likert scales) based on the Intervention Appropriateness Measure (IAM) and the Feasibility of Intervention Measure (FIM) (46). Trained Kenyan research assistants administered all surveys in one-on-one interviews at the PHRD research site using an electronic data platform (CommCare, Dimagi, Cambridge, USA).

### 2.7 Quantitative data analysis

Our primary pilot outcome was the proportion of referred peer clients who initiated PrEP as reported by peer providers (to the best of their knowledge) and by peer clients. We defined PrEP initiation as any PrEP use among PrEP-naïve clients following intervention delivery. Our secondary outcomes included the number of peer clients referred to PrEP services and the proportion of these clients who completed any HIV testing and reported behaviors associated with HIV risk (according to a modified RAST). For our implementation outcomes (e.g., acceptability, appropriateness, feasibility), we considered an outcome construct achieved if ≥80% of participants agreed or strongly agreed with a statement (or disagreed or strongly disagreed with a reverse-coded statement). We used descriptive statistics to assess all quantitative outcomes using Stata v17.0 (StataCorp, College Station, USA).

### 2.8 Qualitative data collection and analysis

One month following pilot completion, we conducted focus group discussions (FGDs) with a subset of enrolled peer providers and clients recruited using convenience sampling. Three study staff trained in qualitative research methods (authors MM, NW, SDR) developed semi-structured FGD guides, which were pilot-tested among young female research staff and refined ahead of implementation. Questions solicited details about participants’ experiences delivering or receiving the intervention, with a specific emphasis on facilitators and barriers which may have influenced the intervention outcomes (e.g., peer recruitment; HIVST uptake; PrEP initiation). All FGDs were conducted at the research site and were moderated by author NW in English and/or Kiswahili (according to participants’ preferences). All FGDs were audio-recorded and transcribed verbatim, with Kiswahili text translated into English, as needed.

To better understand the factors that influenced our quantitative outcomes of interest, we analyzed FGD transcripts using an inductive approach (47, 48). Following each FGD, author NW summarized key topics posed in the interview guides (e.g., facilitators and barriers of peer recruitment; HIVST uptake; PrEP initiation) in debriefing reports. The same key topics were then used by the authors MM and NW to develop a memo template based on facilitators and barriers related to select intervention outcomes. In line with principles of rapid qualitative analysis (49, 50), author MM wrote a memo for each transcript that summarized the FGD content related to the indicated themes, added illustrative quotes, and identified emerging themes (e.g., trust, HIVST demonstrations); all memos were checked, refined, and approved by NW. Finally, author MM with the support of authors NW, SDR, and KFO, identified significant intervention facilitators and barriers and mapped these against quantitative data to triangulate the findings (36, 37).

### 2.9 Ethics statement

The study was approved by the Scientific Ethics Review Unit of the Kenya Medical Research Institute (Nairobi, Kenya: 0247/4349) and the Institutional Review Board of the Fred Hutchinson Cancer Center (Seattle, USA: 10773). All participants provided written informed consent and received 300 Kenyan shillings (KES;~$3 United States Dollars [USD]) for each survey completed and up to 700 KES (~$6 USD) for reimbursed travel to the research site, if needed. Peer providers received no compensation for delivering the intervention. A subset of participants received an additional 500 KES (~$4 USD) for participation in a FGD.

## 3 Results

**Figure 1:**
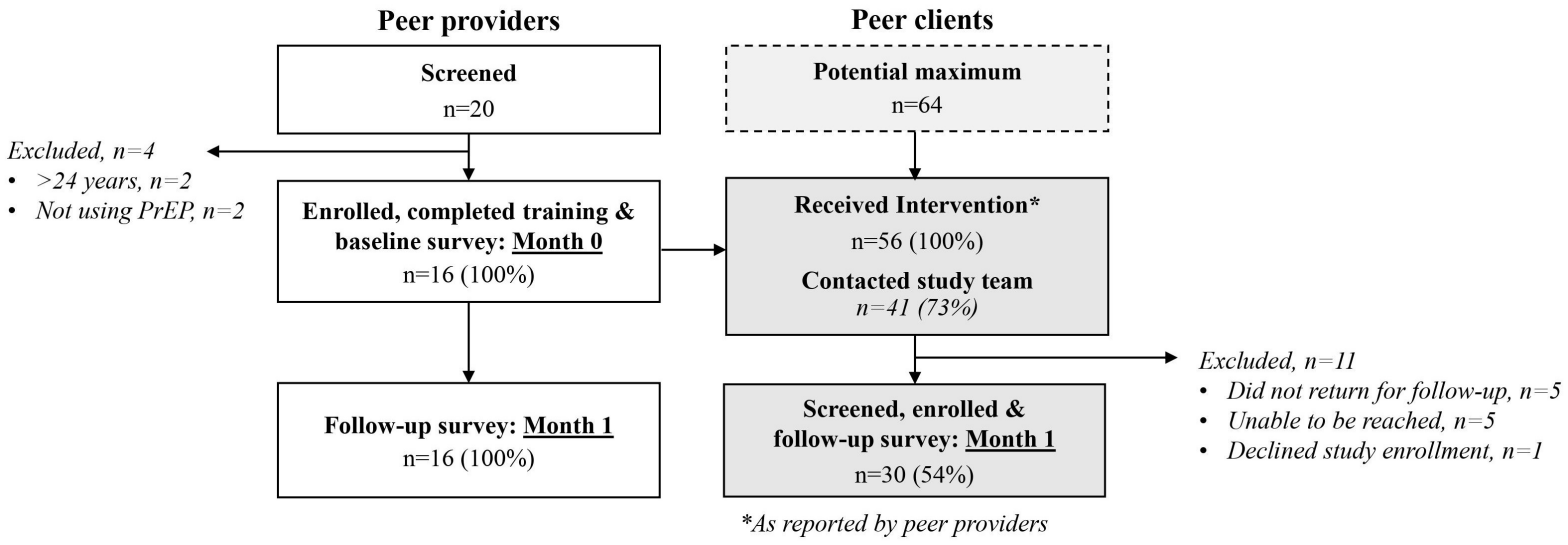
CONSORT diagram for recruitment, enrollment, and follow-up of peer providers and clients.

From March to May 2022, we screened 20 potential peer providers and enrolled and trained 16 (Figure 1). Of the peer providers enrolled in the study, 88% (14/16) were recruited through an HIV prevention program (e.g., DREAMS) and identified themselves as a PrEP champion. At one month, peer providers reported delivering the intervention to 56 total peer clients (median: 4 clients/provider, interquartile range [IQR] 3–4); representing 88% (56/64) completion of the delivery target. Of the 56 peer clients who received the intervention, 41 (73%) contacted the study team and 30 (54%) completed follow-up surveys. Among the 30 peer clients reached for follow-up, the median time from intervention delivery to follow-up was 2 months (IQR 1–3 months).

### 3.1 Participant demographics

The median age of peer providers was 23 years (IQR 21–24) and of peer clients was 21 years (IQR 19–22), and the median years of education was 14 years (IQR 12–15) for peer providers and 13 years (IQR 12–14) for peer clients. At the point of recruitment, half of peer providers (50%, 8/16) reported using PrEP for more than 6 months and half (50%, 8/16) reported PrEP initiation in the past 3–6 months. Nearly all peer clients (97%, 29/30) reported at least one behavior associated with HIV risk; four peer clients (13%) reported they were already using PrEP at the point of intervention delivery (Table 1).

**Table 1:** Demographic characteristics of peer providers and peer clients at enrollment.

Demographics	Peer providers: (*n* = 16) Month 0	Peer clients: (*n* = 30) Month 1
Age (median, IQR)	23 (21–24)	21 (19–22)
Years of education (median, IQR)	14 (12–15)	13 (12–14)
Monthly income, in Kenyan Shillings (USD)^1^		
No Income	3 (19%)	13 (43%)
<5,000 (<$44 USD)	2 (13%)	2 (7%)
5,000–10,000 ($44–88 USD)	7 (43%)	9 (30%)
11,000–20,000 ($97–176 USD)	3 (19%)	4 (13%)
>20,000 (> $176 USD)	1 (6%)	2 (7%)
Relationship status		
Casual partner(s) only	8 (50%)	22 (73%)
Primary partner only	6 (37%)	5 (17%)
Primary partner and casual partner(s)	0 (0%)	3 (10%)
Single, no partners	2 (13%)	0 (0%)
Has child(ren)	8 (50%)	9 (30%)
Self-reported HIV status as “negative”	16 (100%)	30 (100%)
Months prior to enrolling in the study when first started PrEP		
No prior PrEP use	0 (0%)	26 (86%)
3–6 months	8 (50%)	2 (7%)
> 6 months	8 (50%)	2 (7%)
Has disclosed PrEP use to others^2,3^	16 (100%)	–
To a peer	14 (88%)	–
To sexual partner(s)	2 (13%)	–
To family member(s)	2 (13%)		–
Previous experience as a PrEP educator/champion^3,4^	14 (88%)	–
Experienced social harm (verbal, emotional, physical abuse), past 3 months^5^	2 (13%)	2 (7%)
HIV risk behaviors^6^		
Number of sexual partners, past 3 months (median, IQR)	1 (1–1)	1 (1–2)
Has a sexual partner of unknown or positive HIV status	6 (38%)	16 (54%)
In the past 6 months^2^…		
Engaged in condomless sex with partner(s) of unknown or positive HIV status	11 (69%)	24 (80%)
Exchanged sex for money or gifts	3 (19%)	11(37%)
Used emergency contraception	3 (19%)	7 (23%)
Was diagnosed with an STI	1 (6%)	6 (20%)
Used post-exposure prophylaxis (PEP) ≥2 times	1 (6%)	2 (7%)

IQR, Interquartile range; PrEP, pre-exposure prophylaxis; HIVST, HIV self-test; KES, Kenyan shillings; STI, sexually transmitted infection; PEP, post-exposure prophylaxis.

^1^Calculated using the KES to USD exchange rate on March 1, 2022 of 113.78 KES to 1 USD (exchangerates.org.uk).

^2“Select all that apply” question.^

^3Asked only of peer providers.^

^4Received training about PrEP and how to promote it among peers from other studies or programs on HIV prevention among AGYW.^

^5^Experienced social harms by anyone (i.e., family member; sexual partner; community member; peer) in the past 3 months.

^6Majority of HIV risk behaviors were measured through a modified version of the RAST.^

### 3.2 Quantitative findings

#### 3.2.1 HIVST uptake

HIVST use was high among peer clients (Table 2). According to peer providers, 95% (53/56) of clients referred to PrEP services completed at least one HIVST kit; according to surveyed peer clients, 97% (29/30) reported using at least one HIVST kit and 45% (13/29) reported using both HIVST kits. Among peer clients who did not use both HIVST kits (*n* = 17), 14 (82%) said they saved the second kit for later use, two said they only received one HIVST kit, and one said she distributed the second HIVST kit to a sexual partner. Most peer providers (88%, 14/16) reported assisting clients with HIVST use and/or interpretation. Among peer clients who completed HIVST and shared their test results, almost all reported testing HIV-negative (98%, 51/52 according to peer providers; 100%, 29/29 according to peer clients); only one peer client tested HIV-positive according to their peer provider.

**Table 2:** Study outcomes as reported by peer providers and peer clients at 1 month.

	Peer provider reported outcomes		Peer client reported outcomes
	Peer provider outcome (*n* = 16)	Peer client outcome (*n* = 56)	Peer client outcome (*n* = 30)
Intervention delivery outcomes			
Peer clients received educational brochures	–	56/56 (100%)	–
Peer clients received two HIVST kits	–	–	28/30 (93%)
HIVST outcomes			
Peer clients’ used at least one HIVST	–	53/56 (95%)	29/30 (97%)
Used both HIVST kits	–	–	13/29 (45%)
Used only one HIVST kit	–	–	16/29 (55%)
Peer provider assisted any peer client(s) with HIVST kit use^1^	14/16 (88%)	–	24/29 (83%)
Peer clients’ disclosed HIV status to peer provider^1^	–	52/56 (93%)	26/29 (90%)
Peer client tested HIV-negative^1^	–	51/52 (98%)	29/29 (100%)
PrEP initiation outcomes			
Peer client initiated PrEP^2,3^	–	43/55 (78%)	22/26 (85%)
Peer provider disclosed PrEP use to any peer clients^4^	16/16 (100%)	–	22/29 (76%)
Peer provider accompanied peer client to HIV services^3^	–	–	9/26 (35%)
PrEP adherence support			
Peer providers delivered PrEP adherence support	13/16 (81%)	–	–
Types of adherence support provided^5^			
Phone call	9/13 (69%)	–	–
In-person support	5/13 (38%)	–	–
SMS reminders	4/13 (31%)	–	–
Other	2/13 (15%)	–	–

HIVST¸ HIV self-testing; PrEP, pre-exposure prophylaxis; SMS, short message service.

^1As reported by n = 29 peer clients who reported using at least one HIVST kit.^

^2As reported by n = 55 peer clients, excluding one peer client reported as HIV-positive.^

^3As reported by n = 26 peer clients, excluding four peer clients using PrEP at the point of intervention delivery.^

^4As reported by n = 29 peer clients, excluding one participant who did not answer the question.^

^5As reported by n = 13 peer providers who provided adherence support to peer clients.^

#### 3.2.2 PrEP initiation

PrEP initiation among PrEP-naïve peer clients following intervention delivery was high; 78% (43/55) according to peer providers and 85% (22/26) according to peer clients (Table 2). Additionally, the one peer client who tested HIV-positive following intervention delivery, initiated ART (according to their peer provider). Several (35%, 9/26) PrEP-naïve peer clients reported being accompanied to an HIV clinic by a peer provider to initiate PrEP. Additionally, most (81%, 13/16) peer providers reported supporting peer clients with PrEP adherence; the most common forms of adherence support were phone calls (69%, 9/13), in-person reminders (38%, 5/13), and SMS reminders (31%, 4/13).

#### 3.2.3 Acceptability, appropriateness, and feasibility

**Figure 2:**
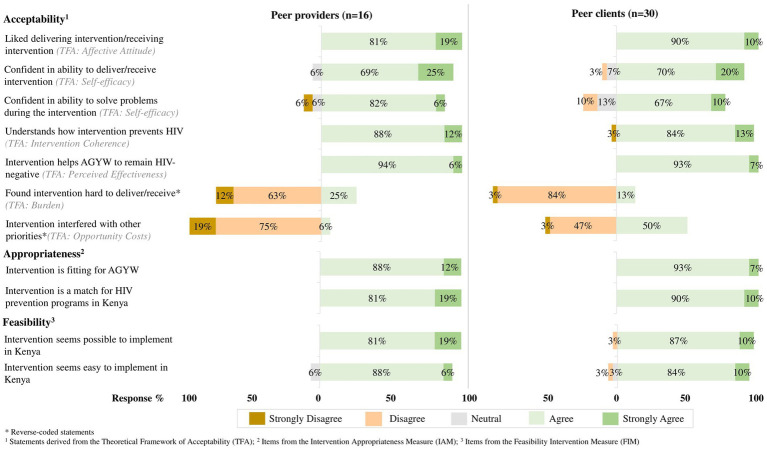
The intervention’s perceived acceptability, appropriateness, and feasibility among peer providers and peer clients.

The intervention’s perceived acceptability, appropriateness, and feasibility was high among peer providers and clients (Figure 2). Most peer providers (≥88%) and peer clients ( ≥ 87%) agreed or strongly agreed with at least five of seven statements assessing different constructs of acceptability. For example, almost all peer providers and clients liked delivering or receiving the intervention (*TFA: affective attitude*) and thought that it helped AGYW remain HIV-negative (*TFA: perceived effectiveness*). The only acceptability constructs that did not meet our response threshold of ≥ 80% agreement (or disagreement for reverse coded statements) were those assessing burden, self-efficacy, and opportunity costs; only 75% (12/16) of peer providers disagreed to some extent that the intervention was hard to deliver (*TFA: burden*). Among peer clients, only 77% (23/30) agreed that they felt confident to solve problems during the intervention (*TFA: self-efficacy*) and 50% (15/30) agreed that the intervention did not interfere with their other priorities (*TFA: opportunity cost*). All peer providers and clients agreed or strongly agreed with statements measuring intervention appropriateness and feasibility, indicating that they thought the intervention was fitting for AGYW and a match with HIV prevention programs in Kenya (IAM), and seemed possible and easy to implement (FIM).

### 3.3 Qualitative findings

From July to September 2022, we conducted five FGDs in group sizes of four to five participants; two FGDs with peer providers and three with peer clients. Roughly half of peer providers (56%, 9/16) and peer clients (43%, 13/30) participated in these FGDs. The median age of FGD peer providers was 24 years (IQR 23–24 years) and of FGD peer clients was 22 years (IQR 20–22 years). Among the 13 FGD peer clients, nine initiated PrEP following the intervention, two were using PrEP at the point of intervention delivery, and two remained PrEP-naïve.

Both participant groups identified factors that they perceived challenged or facilitated the following pilot outcomes: successful recruitment of peers at HIV risk, high HIVST uptake, and high PrEP initiation (Table 3). For example, peer clients described how they were motivated to accept the intervention because they had a close pre-existing relationship with the provider delivering it and perceived them to be knowledgeable about HIV and PrEP. Challenges with peer recruitment, however, included conflicting priorities (e.g., work, school) among providers and clients as well as limited privacy at the point of recruitment.

**Table 3:** Understanding the pilot outcomes with insights from five focus group discussions with select peer providers and clients.

	Possible drivers of quantitative outcomes	Exemplary quotes
Successful recruitment of peers at HIV risk	(+) Formalized peer provider training helped educate providers and gave them confidence to deliver the intervention	*“Since we were trained and, for me, I understood every part of what we were taught, I was good to answer any question that was brought up by those we were referring [peer clients].”* (Peer provider, FGD 1, P5)
	(+) Preexisting relationships between clients and peer providers reinforced the perception among clients that peer providers sincerely cared about their wellbeing and increased their engagement in the intervention	*“[The peer provider] approached me well—in a friendly way —and what even made me happier is that you know there is someone somewhere that cares about you not find[ing] yourself in a mess … [and becoming] HIV-positive. So when you see that someone is caring in such a way, you feel good and you can open up to them…”* (Peer client, FGD 1, P1)
	(+) Providing accurate information about HIV and PrEP motivated some clients to trust their peer provider and engage in the intervention	*“As for me since, I already had past knowledge [about PrEP], all that she [the peer provider] was telling me, I trusted it since I also knew that is how it goes.”* (Peer client, FGD 1, P4)
	(−) Limited resources (e.g., transport fare) and conflicting priorities (e.g., work, school) made it challenging for some peer providers and clients to deliver and receive the intervention, respectively	*“Some of our friends [peer clients] live very far, so you want to go [reach them] but you find that you do not have [transportation] fare. So until the time you will get the fare, that is when you will go. So it is a challenge to me.”* (Peer provider, FGD 1, P5)
	(−) Limited privacy at the point of intervention delivery made some clients hesitant to engage in the intervention	*“You know, she [the peer provider] had come with my sister first. So they were trying to convince me [to do the HIV test], … but I was just refusing. But afterwards, she [the peer provider] came alone, we sat and talked well, and it became easy [to accept the intervention].”* (Peer client, FGD 2, P2)
High HIVST uptake among peer clients	(+) Peer provider assistance with HIVST use and interpretation helped motivate clients to use the kits (with some providers using HIVST demonstration kits)	*“She [the peer provider] opened the kit and explained how I should wipe [sanitize], and what to do with the blood, then when the results show… if it is one line, then it is negative, and if it shows two lines, it is positive.”* (Peer client, FGD 1, P4)
	(+) Prior HIVST experience or novelty of HIVST motivated some clients to use the HIVST kit	*“For me, that experience [doing blood-based HIVST] was just good because I am used to this other kit [rapid clinic-based testing], I was not used to that one [HIVST]. I saw it for the first time. So I was happy because I also got the knowledge of using this other one and not the usual one that people are used to..”* (Peer client, FGD 2, P4)
	(−) Lack of prior experience doing HIVST made some clients hesitant to use the kits	*“The [blood-based HIVST] process itself [was challenging] because I had never used it before. The one I was used to using is the one for saliva [oral-fluid HIVST], so this one was a bit complicated to use.”* (Peer client, FGD 1, P1)
High PrEP initiation among peer clients	(+/−) Peer provider accompaniment to the clinic for linkage to specific PrEP providers helped some clients initiate PrEP, but some peer providers who opted to do this found it burdensome (e.g., time commitment)	*“…she [the peer provider] took me to the hospital [for PrEP services], and we sat together and were given PrEP… [Thereafter,] I would go [to the hospital for PrEP] alone. But for the first time, she escorted me.”* (Peer client, FGD 1, P3) *“For me, I found it [accompanying clients to the clinic for PrEP services] to be tiresome a lot because maybe I’m also busy… I felt that it was straining me regarding my finances and time.”* (Peer provider, FGD 2, P3)
	(+) Providers’ disclosure of their own PrEP use motivated some clients to initiate PrEP	*“PrEP was new to me. I have never heard about it before, but I was like something new, you need to know, you need to pay attention, then you listen. Then she [the peer provider] told me she had experienced it [taken PrEP] and challenged me to try one and see.”* (Peer client, FGD 2, P1)

(+) Factors facilitated pilot outcomes.

(−) Factors challenged pilot outcomes.

P, Participant.

Insights from the FGDs suggested that the high HIVST uptake among peers was at times motivated by the novelty of the technology, the desire to learn something new, and assistance from peer providers. A remaining barrier to HIVST identified by peers, however, included limited experience with HIVST kits. To make peer clients feel more comfortable with the HIVST process, some peer providers used one of the HIVST kits intended for clients to demonstrate kit use. Then, the high observed PrEP initiation in the pilot could have been attributable to support peer clients received from peer providers, which included providers sharing their own experiences with PrEP use and accompanying peer clients to the clinic, if requested. While peer clients expressed accompaniment as a strong motivator, providers often described this additional level of support as burdensome in terms of time commitment and resources (e.g., transport fares) required.

## 4 Discussion

This mixed-methods pilot study demonstrated that a formalized model of peer PrEP referral supported with HIVST delivery resulted in high PrEP initiation among Kenyan AGYW. Additionally, the model was found to be acceptable, appropriate, and feasible among both AGYW who delivered and received the intervention. In FGDs with AGYW participants, the close existing relationships between peers, assisted HIVST use by peer providers, and peer provider PrEP disclosure and accompaniment to clinics were identified as contributing to the successful peer recruitment, HIVST use, and PrEP initiation observed in the pilot study.

In this pilot, PrEP initiation among peer clients was facilitated by peer provider support, often in the form of peer accompaniment to HIV clinics. While this support was beneficial to peer clients, some peer providers reported it as a challenge in terms of the financial resources they needed to pay for transport fares and its conflict with their other priorities. In future iterations of this model, transportation vouchers could be considered for peer providers to support them in accompanying peer clients to healthcare clinics for HIV services (51). Additionally, future peer-delivered interventions might consider peer providers delivering one bottle of PrEP (i.e., 30 pills) directly to clients if they meet certain criteria (i.e., no history of liver or kidney disease (52)). Then clients could self-initiate PrEP services once they confirm they are HIV-negative via self-testing (53) and later link to a healthcare clinic for follow-up care.

While most peer clients recruited initiated PrEP in this pilot study, not all peer providers recruited the recommended number of peer clients; thus, opportunities remain to increase the number of peer clients reached with the intervention. One challenge with peer referral in this pilot might have been the saturation of HIV prevention programs tailored to AGYW (41), which already engaged most AGYW in the region who could benefit from PrEP. To expand the reach of PrEP services, peer providers could be recruited for this intervention outside of these existing PrEP programs and in settings where AGYW might have less access to HIV services, such as informal settlements or rural communities. Then, the peer-delivered nature of the intervention and associated snowball sampling (per the intervention design) might reach more PrEP-naïve AGYW who could benefit from HIV prevention services. Future peer-delivered interventions might also consider incorporating a train-the-trainer approach (54, 55), where recruited peer clients are trained in a subsequent wave of peer providers to increase intervention reach.

PrEP adherence support was not explicitly part of the intervention design—which primarily focused on linking AGYW to PrEP services—but was often reported among peer providers in this pilot study. This finding suggests that adherence support delivered by peers may be a normative and acceptable practice that could support continued PrEP use among AGYW. Future iterations of this peer-delivered model may benefit from formally adding an adherence component to support recruited peer clients that initiate PrEP (2, 56). Studies conducted among African AGYW often report poor PrEP continuation (i.e., refilling) due to internal and external factors, such as PrEP stigma, side effects, limited social support, and travel distance to the clinic (21, 57–59). To support continued PrEP use, peer providers could encourage peer clients to identify a treatment buddy who could hold them accountable for taking PrEP or could encourage active participation in an (virtual) adherence support club (60, 61). Alternatively, peer providers could deliver PrEP refills directly to peer clients; an intervention that has been implemented successfully among AGYW in various African settings including Kenya and Uganda (20, 62).

This study is not without limitations. First, most of the peer providers in this study were recruited from existing HIV prevention programs tailored to AGYW in the region—specifically, the DREAMS program (41)—and thus identified themselves as PrEP champions. Any prior training peer providers received from these programs might have inflated our PrEP initiation outcome if they used existing skills to recruit and refer peers to PrEP. Additionally, peer providers’ participation in other HIV prevention programs might have limited the generalizability of our study findings to other settings where such programs do not exist. Second, the percentage of recruited peer clients that reported PrEP use at the point of intervention delivery (13%, 4/30) was high for AGYW in Kenya, which suggests that peer providers might not have recruited a representative sample of AGYW in the region, thus limiting the generalizability of our findings. Third, we did not compensate peer providers for their efforts referring peer clients to PrEP with HIVST kits and did not assess how much they spent on transport to reach peer clients. Future iterations of this model might consider compensating peer providers per peer client who initiated PrEP or providing transportation vouchers to support intervention delivery, both which are likely to increase the impact of the intervention. Finally, the study outcomes reported by peer clients might have been subject to selection and desirability biases. The peer clients who contacted study staff for enrollment and participated in follow-up surveys might have had more time, economic resources (i.e., a phone or airtime), or interest in PrEP than those who did not, which may have inflated our HIVST use and PrEP initiation outcomes. Additionally, privacy concerns related to highly-stigmatized information (i.e., HIV status) might have prevented peer clients from sharing accurate information with peer providers and study staff. These concerns are attenuated, however, by the fact that our outcomes were similar when reported by peer providers and clients.

## 5 Conclusion

This pilot study suggests that peer referral to HIV PrEP services supported with HIVST has great potential to increase PrEP initiation among Kenyan AGYW. This intervention is promising as it leverages an existing common referral mechanism for AGYW (i.e., word-of-mouth referral) and utilizes HIVST in a new way—to confirm an HIV-negative status and support referral to prevention interventions. Now that our study demonstrated the acceptability, appropriateness, and feasibility of this intervention in this pilot study, more research is needed to understand how this intervention might be delivered and sustained at scale in real-world public clinics. Specifically, more research is needed on the feasibility of training young female PrEP users on the intervention components and the costs associated with intervention delivery outside a research setting; information that would be necessary to persuade the Kenyan Ministry of Health or an implementing organization to invest in formalized peer PrEP referral with HIVST delivery to improve PrEP initiation among AGYW.

## Data Availability

The data supporting the conclusions of this article will be made available by the authors.

## Footnotes

^1^ ClinicalTrials.gov

## References

[1] UNAIDS. Kenya launches self-test kits and PrEP [internet]. Geneva, Switzerland: UNAIDS (2017). Available at: https://www.unaids.org/en/resources/presscentre/featurestories/2017/may/20170505_kenya (Accessed November 25, 2022).

[2] National AIDS & STI Control Program. Framework for the implementation of pre-exposure prophylaxis of HIV in Kenya. Nairobi, Kenya: NASCOP (2017). 84 p.

[3] Katz IT Ngure K Kamolloh K Ogello V Okombo M Thuo NB. Multi-level factors driving pre-exposure prophylaxis non-initiation among young women at high risk for HIV in Kenya. AIDS Behav. (2023) 27:106–18. doi: 10.1007/s10461-022-03748-9 35930203

[4] Sila J Larsen AM Kinuthia J Owiti G Abuna F Kohler PK. High awareness, yet low uptake, of pre-exposure prophylaxis among adolescent girls and young women within family planning clinics in Kenya. AIDS Patient Care STDs. (2020) 34:336–43. doi: 10.1089/apc.2020.0037 32757980 PMC7415219

[5] National AIDS & STI Control Program. Kenya population-based HIV impact assessment: KENPHIA 2018. Nairobi, Kenya: NASCOP (2022). 322 p.

[6] National AIDS Control Council. Kenya AIDS strategic framework II. Nairobi, Kenya: NACC (2021). 80 p.

[7] Camlin CS Koss CA Getahun M Owino L Itiakorit H Akatukwasa C. Understanding demand for PrEP and early experiences of PrEP use among young adults in rural Kenya and Uganda: a qualitative study. AIDS Behav. (2020) 24:2149–62. doi: 10.1007/s10461-020-02780-x 31955361 PMC7909847

[8] Mugo NR Ngure K Kiragu M Irungu E Kilonzo N. PrEP for Africa: what we have learnt and what is needed to move to program implementation. Curr Opin HIV AIDS. (2016) 11:80–6. doi: 10.1097/COH.0000000000000224, PMID: 26575147 PMC4900687

[9] Ziraba A Orindi B Muuo S Floyd S Birdthistle IJ Mumah J. Understanding HIV risks among adolescent girls and young women in informal settlements of Nairobi, Kenya: lessons for DREAMS. PLoS One. (2018) 13:e0197479–20. doi: 10.1371/journal.pone.0197479, PMID: 29851988 PMC5978990

[10] Patton GC Sawyer SM Santelli JS Ross DA Afifi R Allen NB. Our future: a lancet commission on adolescent health and wellbeing. Lancet. (2016) 387:2423–78. doi: 10.1016/S0140-6736(16)00579-1 27174304 PMC5832967

[11] National AIDS Control Council. Kenya’s fast-track plan to end HIV and AIDS among adolescents and young people. Nairobi, Kenya: NACC (2015). 68 p.

[12] Wagner AD Wilson KS Babigumira JB Mugo C Mutiti PM Neary J. Can adolescents and young adults in Kenya afford free HIV testing services? J Assoc Nurses AIDS Care. (2020) 31:483–92. doi: 10.1097/JNC.0000000000000012, PMID: 30585863 PMC6586552

[13] Murewanhema G Musuka G Moyo P Moyo E Dzinamarira T. HIV and adolescent girls and young women in sub-Saharan Africa: a call for expedited action to reduce new infections. IJID Reg. (2022) 5:30–2. doi: 10.1016/j.ijregi.2022.08.009, PMID: 36147901 PMC9485902

[14] Shangani S Escudero D Kirwa K Harrison A Marshall B Operario D. Effectiveness of peer-led interventions to increase HIV testing among men who have sex with men: a systematic review and meta-analysis. AIDS Care. (2017) 29:1003–13. doi: 10.1080/09540121.2017.1282105, PMID: 28150501 PMC5570465

[15] Nyato D Kuringe E Drake M Casalini C Nnko S Shao A. Participants’ accrual and delivery of HIV prevention interventions among men who have sex with men in sub-Saharan Africa: a systematic review. BMC Public Health. (2018) 18:1–14. doi: 10.1186/s12889-018-5303-2, PMID: 29554867 PMC5859521

[16] Ortblad KF Kibuuka Musoke D Ngabirano T Nakitende A Magoola J Kayiira P. Direct provision versus facility collection of HIV self-tests among female sex workers in Uganda: a cluster-randomized controlled health systems trial. PLoS Med. (2017) 14:1–24. doi: 10.1371/journal.pmed.1002458 PMC5705079 29182634

[17] Chersich MF Luchters S Ntaganira I Gerbase A Lo Y-R Scorgie F. Priority interventions to reduce HIV transmission in sex work settings in sub-Saharan Africa and delivery of these services. J Int AIDS Soc. (2013) 16:1–8. doi: 10.7448/IAS.16.1.17980, PMID: 23462140 PMC3589546

[18] Lillie TA Persaud NE DiCarlo MC Gashobotse D Kamali DR Cheron M. Reaching the unreached: performance of an enhanced peer outreach approach to identify new HIV cases among female sex workers and men who have sex with men in HIV programs in west and Central Africa. PLoS One. (2019) 14:e0213743–11. doi: 10.1371/journal.pone.0213743, PMID: 30943205 PMC6447144

[19] Jackson-Gibson M Ezema AU Orero W Were I Ohiomoba RO Mbullo PO. Facilitators and barriers to HIV pre-exposure prophylaxis (PrEP) uptake through a community-based intervention strategy among adolescent girls and young women in Seme Sub-County, Kisumu, Kenya. BMC Public Health. (2021) 21:1–13. doi: 10.1186/s12889-021-11335-1, PMID: 34210288 PMC8252310

[20] Nakalega R Mukiza N Menge R Kizito S Babirye JA Kuteesa CN. Feasibility and acceptability of peer-delivered HIV self-testing and PrEP for young women in Kampala, Uganda. BMC Public Health. (2023) 23:1–12. doi: 10.1186/s12889-023-16081-0, PMID: 37322510 PMC10273744

[21] O’Rourke S Hartmann M Myers L Lawrence N Gill K Morton JF. The PrEP journey: understanding how internal drivers and external circumstances impact the PrEP trajectory of adolescent girls and young women in Cape Town, South Africa. AIDS Behav. (2021) 25:2154–65. doi: 10.1007/s10461-020-03145-0, PMID: 33521908 PMC8651257

[22] McGowan M Casmir E Wairimu N Mogere P Jahn A Ngure K. Assessing young Kenyan women’s willingness to engage in a peer-delivered HIV self-testing and referral model for PrEP initiation: a qualitative formative research study. Front Public Health. (2022) 10:1–12. doi: 10.3389/fpubh.2022.932948 PMC9583529 36276357

[23] Rousseau E Katz AWK O’Rourke S Bekker L-G Delany-Moretlwe S Bukusi E. Adolescent girls and young women’s PrEP-user journey during an implementation science study in South Africa and Kenya. PLoS One. (2021) 16:e0258542–18. doi: 10.1371/journal.pone.0258542, PMID: 34648589 PMC8516266

[24] Adeagbo OA Seeley J Gumede D Xulu S Dlamini N Luthuli M. Process evaluation of peer-to-peer delivery of HIV self-testing and sexual health information to support HIV prevention among youth in rural KwaZulu-Natal, South Africa: qualitative analysis. BMJ Open. (2022) 12:1–9. doi: 10.1136/bmjopen-2021-048780, PMID: 35165105 PMC8845207

[25] Matovu JKB Nambuusi A Nakabirye S Wanyenze RK Serwadda D. Formative research to inform the development of a peer-led HIV self-testing intervention to improve HIV testing uptake and linkage to HIV care among adolescents, young people and adult men in Kasensero fishing community, Rakai, Uganda: a qualitative study. BMC Public Health. (2020) 20:1582–16. doi: 10.1186/s12889-020-09714-1, PMID: 33081735 PMC7576713

[26] Chanda MM Ortblad KF Mwale M Chongo S Kanchele C Kamungoma N. HIV self-testing among female sex workers in Zambia: a cluster randomized controlled trial. PLoS Med. (2017) 14:e1002442–19. doi: 10.1371/journal.pmed.1002442, PMID: 29161260 PMC5697803

[27] Witzel TC Eshun-Wilson I Jamil MS Tilouche N Figueroa C Johnson CC. Comparing the effects of HIV self-testing to standard HIV testing for key populations: a systematic review and meta-analysis. BMC Med. (2020) 18:381–13. doi: 10.1186/s12916-020-01835-z, PMID: 33267890 PMC7713313

[28] Oldenburg CE Chanda MM Ortblad KF Mwale M Chongo S Kamungoma N. Effect of HIV self-testing on the number of sexual partners among female sex workers in Zambia. AIDS. (2018) 32:645–52. doi: 10.1097/QAD.0000000000001740, PMID: 29494424 PMC5844591

[29] Ortblad KF Musoke DK Ngabirano T Salomon JA Haberer JE McConnell M. Is knowledge of HIV status associated with sexual behaviours? A fixed effects analysis of a female sex worker cohort in urban Uganda. JIAS. (2019) 22:1–11. doi: 10.1002/jia2.25336, PMID: 31287625 PMC6615530

[30] Ortblad KF Chanda MM Mwale M Haberer JE Mcconnell M Oldenburg CE. Perceived knowledge of HIV-negative status increases condom use among female sex workers in Zambian transit towns. AIDS Patient Care STDs. (2020) 34:184–92. doi: 10.1089/apc.2019.0266, PMID: 32324483 PMC7194317

[31] Korte JE Kisa R Vrana-Diaz CJ Malek AM Buregyeya E Matovu JKB. HIV oral self-testing for male partners of women attending antenatal care in Central Uganda: uptake of testing and linkage to care in a randomized trial. J Acquir Immune Defic Syndr. (2020) 84:271–9. doi: 10.1097/QAI.0000000000002341, PMID: 32168168

[32] Shapiro AE Van Heerden A Krows M Sausi K Sithole N Schaafsma TT. An implementation study of oral and blood-based HIV self-testing and linkage to care among men in rural and peri-urban KwaZulu-Natal, South Africa. J Int AIDS Soc. (2020) 23:35–42. doi: 10.1002/jia2.25514 PMC7319114 32589337

[33] Chipungu J Bosomprah S Zanolini A Thimurthy H Chilengi R. Understanding linkage to care with HIV self-test approach in Lusaka, Zambia - a mixed method approach. PLoS One. (2017) 12:e0187998–12. doi: 10.1371/journal.pone.0187998, PMID: 29149194 PMC5693414

[34] Hlongwa M Moyo E Dzinamarira T. Approaches for improving linkage to HIV care among HIV self-testing individuals in sub-Saharan Africa. BMJ Glob Health. (2023) 8:1–5. doi: 10.1136/bmjgh-2023-012664 PMC10351227 37451688

[35] Wairimu N Mogere P Mc Gowan M Casmir E Mugo N. Design of a peer PrEP referral + HIV self-testing delivery model to increase PrEP initiation among young Kenyan women: use of the nominal group technique. Oral presentation at the University of Nairobi STD/HIV/SRH Collaborative Research Group Meeting (2023); Nairobi, Kenya.

[36] Palinkas LA Mendon SJ Hamilton AB. Innovations in mixed methods evaluations. Annu Rev Public Health. (2019) 40:423–42. doi: 10.1146/annurev-publhealth-040218-044215, PMID: 30633710 PMC6501787

[37] Ivankova NV Creswell JW Stick SL. Using mixed-methods sequential explanatory design: from theory to practice. Field Methods. (2006) 18:3–20. doi: 10.1177/1525822X05282260

[38] National Syndemic Diseases Control Council. Kenya AIDS strategic framework II: 2020/21-2024/25 mid term review report. Nairobi, Kenya: NSDCC (2024). 136 p.

[39] National AIDS & STI Control Program. Guidelines for conducting adolescent HIV sexual and reproductive health research in Kenya. Nairobi, Kenya: NASCOP (2015). 48 p.

[40] Wairimu N Malen RC Reedy AM Mogere P Njeru I Culquichicón C. Peer PrEP referral + HIV self-test delivery for PrEP initiation among young Kenyan women: study protocol for a hybrid cluster-randomized controlled trial. Trials. (2023) 24:1–12. doi: 10.1186/s13063-023-07734-x 37925450 PMC10625301

[41] U.S. Agency for International Development. DREAMS: Partnership to reduce HIV/AIDS in adolescent girls and young women. Washington D.C., USA: USAID (2024) Available at: https://www.usaid.gov/global-health/health-areas/hiv-and-aids/technical-areas/dreams (Accessed March 13, 2024).

[42] U.S. Food & Drug Administration. Chembio SURE CHECK HIV 1/2 ASSAY. Silver Spring, USA: FDA (2006). 15 p.

[43] National AIDS & STI Control Program. Pre-exposure prophylaxis for the prevention of HIV infection: A toolkit for providers. Nairobi, Kenya: NASCOP (2017). 57 p.

[44] Sekhon M Cartwright M Francis JJ. Acceptability of healthcare interventions: an overview of reviews and development of a theoretical framework. BMC Health Serv Res. (2017) 17:88–13. doi: 10.1186/s12913-017-2031-8, PMID: 28126032 PMC5267473

[45] Sekhon M Cartwright M Francis JJ. Development of a theory-informed questionnaire to assess the acceptability of healthcare interventions. BMC Health Serv Res. (2022) 22:279–12. doi: 10.1186/s12913-022-07577-3, PMID: 35232455 PMC8887649

[46] Weiner BJ Lewis CC Stanick C Powell BJ Dorsey CN Clary AS. Psychometric assessment of three newly developed implementation outcome measures. Implement Sci. (2017) 12:108–12. doi: 10.1186/s13012-017-0635-3, PMID: 28851459 PMC5576104

[47] Patton MQ. Qualitative research & evaluation methods: Integrating theory and practice. 4th ed. Thousand Oaks, USA: Sage publications (2014). 808 p.

[48] Pope C Ziebland S Mays N. Qualitative research in health care: Analysing qualitative data. BMJ. (2000) 320:114–6. doi: 10.1136/bmj.320.7227.114 10625273 PMC1117368

[49] Vindrola-Padros C Johnson GA. Rapid techniques in qualitative research: a critical review of the literature. Qual Health Res. (2020) 30:1596–604. doi: 10.1177/1049732320921835 32667277

[50] Hamilton A. Qualitative methods in rapid turn-around health services research. Washington D.C, USA: U.S. Department of Veteran Affairs Cyber Seminar (2013).

[51] Sindelar K Maponga C Lekoala F Mandara E Mohoanyane M Sanders J. Beyond the facility: an evaluation of seven community-based pediatric HIV testing strategies and linkage to care outcomes in a high prevalence, resource-limited setting. PLoS One. (2020) 15:e0236985–16. doi: 10.1371/journal.pone.0236985, PMID: 32877441 PMC7467225

[52] Ortblad KF Mogere P Roche S Kamolloh K Odoyo J Irungu E. Design of a care pathway for pharmacy-based PrEP delivery in Kenya: results from a collaborative stakeholder consultation. BMC Health Serv Res. (2020) 20:1–9. doi: 10.1186/s12913-020-05898-9 PMC7661206 33176785

[53] World Health Organization. Differentiated and simplified pre-exposure prophylaxis for HIV prevention: Update to WHO implementation guidance. Geneva, Switzerland: WHO (2022). 46 p.

[54] Tobias CR Downes A Eddens S Ruiz J. Building blocks for peer success: lessons learned from a train-the-trainer program. AIDS Patient Care STDs. (2012) 26:53–9. doi: 10.1089/apc.2011.0224, PMID: 22103430 PMC3242619

[55] Fordjuoh J Dolezal C Bhengu N Harrison AD Exner TM Hanass-Hancock J. Peer-to-peer chain recruitment for enrolling young south African women into an HIV pre-exposure prophylaxis (PrEP) intervention study: how did it perform? AIDS Behav. (2024) 28:1782–94. doi: 10.1007/s10461-023-04256-0, PMID: 38416275

[56] Irungu EM Baeten JM. PrEP rollout in Africa: status and opportunity. Nat Med. (2020) 26:655–64. doi: 10.1038/s41591-020-0872-x, PMID: 32405065

[57] Kayesu I Mayanja Y Nakirijja C Machira YW Price M Seeley J. Uptake of and adherence to oral pre-exposure prophylaxis among adolescent girls and young women at high risk of HIV-infection in Kampala, Uganda: a qualitative study of experiences, facilitators and barriers. BMC Womens Health. (2022) 22:440–14. doi: 10.1186/s12905-022-02018-z, PMID: 36357920 PMC9648457

[58] de Dieu TJ Cover J Obong OC Brady M Cressey TR Mori K. Continued attendance in a PrEP program despite low adherence and non-protective drug levels among adolescent girls and young women in Kenya: results from a prospective cohort study. PLoS Med. (2022) 19:e1004097–16. doi: 10.1371/journal.pmed.1004097, PMID: 36095005 PMC9521917

[59] Pintye J O’Malley G Kinuthia J Abuna F Escudero JN Mugambi M. Influences on early discontinuation and persistence of daily oral PrEP use among Kenyan adolescent girls and young women: a qualitative evaluation from a PrEP implementation program. JAIDS. (2021) 86:e83–9. doi: 10.1097/QAI.0000000000002587, PMID: 33273211 PMC8935942

[60] Were D Musau A Mugambi M Plotkin M Kabue M Manguro G. An implementation model for scaling up oral pre-exposure prophylaxis in Kenya: Jilinde project. Gates Open Res. (2021) 5:113–4. doi: 10.12688/gatesopenres.13342.1, PMID: 34988373 PMC8669463

[61] Roberts ST Mancuso N Williams K Nabunya HK Mposula H Mugocha C. How a menu of adherence support strategies facilitated high adherence to HIV prevention products among adolescent girls and young women in sub-Saharan Africa: a mixed methods analysis. JIAS. (2023) 26:1–13. doi: 10.1002/jia2.26189/full PMC10630658 37936551

[62] Rousseau E Julies RF Madubela N Kassim S. Novel platforms for biomedical HIV prevention delivery to key populations — community mobile clinics, peer-supported, pharmacy-led PrEP delivery, and the use of telemedicine. Curr HIV/AIDS Rep. (2021) 18:500–7. doi: 10.1007/s11904-021-00578-7 10.1007/s11904-021-00578-7 PMC8549812 34708316

