# Supplementary material for: Formalized peer referral to HIV pre-exposure prophylaxis supported with self-testing: a mixed-methods pilot study among young Kenyan women
Source: Front Public Health. 2024 Sep 11;12:1428609. doi: 10.3389/fpubh.2024.1428609 (PMC11422135; doi:10.3389/fpubh.2024.1428609)
Supplement: Supplementary file 1 [file Data_Sheet_1.pdf]

IRO REC'D 07/12/2022

Questionnaire: Baseline (PrEP User)

This baseline questionnaire is to be conducted by research staff. This baseline questionnaire has 8 parts: 1) demographics, 2) sexual behaviors, 3) depression screening 4) relationships to peers, 5) PrEP, 6) Self-Efficacy: Conducting Peer PrEP referral + HIVST, 7) acceptability of a peer PrEP referral + HIVST model 8) HPV. Participants should do their best to answer all questions and let the researcher know if they feel uncomfortable or prefer not to answer any of the questions.

**0. Logistics**<br>*Let's start with some basic logistics*		
	Baseline Questionnaire Completion Date	[Captured in CommCare]
	Participant ID:	|__|__|-|__|__|-|__|__|__||__|-|__|
	Where is this questionnaire being conducted?	☐ *PHRD*<br>☐ *Other:___________________*
**1. Demographics**<br>*First, I would like to ask you some basic questions about yourself.*		
1.	Age:	|__|__| *number of years*
2.	How many years of school have you completed? [Do not count repeat levels.]	|__|__| *number of years*<br>☐ *Prefer not to answer*
3.	Are you currently enrolled in school?	☐ *Yes*<br>☐ *No*
4.	Are you currently or have you previously been enrolled in an HIV study at the PHRD (e.g., "partners")?	☐ *Yes*<br>☐ *No*
4a	[If Q4 = Yes] Please briefly describe the study you were/are enrolled in.	*Explain:____________________*
5	Are you currently or have you previously been trained as a "PrEP champion" in your community either through the PHRD, DREAMS program, or any other HIV prevention organization?	☐ *Yes*<br>☐ *No*
6.	What is your relationship status?	☐ Single, no partners<br>☐ Casual partner(s) only<br>☐ One primary partner<br>☐ One primary partner and casual partners<br>☐Other:____________________
7.	What is your monthly income? (average of the last 3 months)	|__|__||__|__||__|__||__|__|_|__*kSH*
8.	What is your main source of income?	☐ No income<br>☐ Laborer/semi-skilled<br>☐ Trade/sales<br>☐ Student<br>☐ Professional<br>☐ Farming/animal raising<br>☐ Sex worker

FHCRC IRB Approval
AUG 11 2022
Document Released Date

		☐ Other:___________________<br>☐ Prefer not to answer
9.	How often did you have 4 or more drinks of alcohol (e.g., the equivalent of 4 regular beer bottles) on a single occasion in the past year?<br><br>*(Source: M SASQ- Public Health England, 2020)*	☐ Never<br>☐ Less than monthly<br>☐ Monthly<br>☐ Weekly<br>☐ Daily or almost daily
10.	How many times in the past year have you used an illegal drug or used a prescription medication for non-medical reasons (e.g., because of the experience or feeling it caused)?<br>*(Source: SIP-DU; Smith et al., Archives of Internal Medicine, 2010)*<br>*(Source: SISQ, McNeely et al., Journal of General Internal Medicine, 2015)*	|__|__| *number [enter 0 if never]*<br>☐ *Prefer not to answer*
11.	<u>In the last three months,</u> have you been verbally, physically or emotionally abused by anyone?	☐ *Yes* [*Complete social harm report]*<br>☐ *No*<br>☐ *Prefer not to answer*
**2. Sexual Behaviors**<br>*Now I would like to ask you some questions about your sexual behaviors.*<br>*Source: PrEP RAST, (DREAMS PrIYA, 2017)*		
1.	When did you last test for HIV before today?	_______ *months*
2.	What were the results of your last HIV test?	☐ *HIV-negative*<br>☐ *HIV-positive*<br>☐ *Unknown*<br>☐ *Prefer not to answer*
3.	How do you typically test for HIV?	☐ *Clinic-based: rapid testing*<br>☐ *Pharmacy-based (chemist): rapid testing*<br>☐ *Pharmacy-based (chemist): self-test (assisted/unassisted)*<br>☐ *Home-based: self-test*<br>☐ *Other: _______________*
4.	Who supports you when you go for HIV testing? [check all that apply]	☐ *No one*<br>☐ *Community member*<br>☐ *Family member*<br>☐ *Sexual partner(s)*<br>☐ *Friend/peer*<br>☐ *Other: _______________*
4a	Have you ever tested for HIV using an HIV self-test (e.g., an HIV test conducted outside of a healthcare facility)?	☐ *Yes*<br>☐ *No*<br>☐ *Prefer not to answer*
4b	Where would you most prefer to access HIVST kits from?	☐ *I would not like to access an HIVST kit*<br>☐ *Retail pharmacy*<br>☐ *Health facility*

		☐ *Family planning clinic*<br>☐ *Friend/family member*<br>☐ *Sexual partner(s)*<br>☐ *Other:___________________*
5.	In the past two weeks, how many times did you have sexual intercourse?	|__|__| *number of sex acts*<br>*(if 0, go to Q6)*<br>☐ *Prefer not to answer*
5a.	When you had sex in the past two weeks, how many times was a condom used?	|__|__| *number of times condom used*<br>☐ *Prefer not to answer*
6.	In the past three months, how many individuals have you had sex with?	|__|__| *number of sexual partners (if 0 go to Q8)*<br>☐ *Prefer not to answer*
7.	Of the individuals you had sex with in the past three months, how many are new sexual partners?	|__|__| *number of new sexual partners*<br>☐ *Prefer not to answer*
8.	Of your sexual partners, how many do you know their HIV status?	|__|__| *number of sexual partners*<br>☐ *N/A have no current sexual partners*<br>☐ *Don't know*<br>☐ *Prefer not to answer*
9.	Do you have a primary sexual partner?	☐ *Yes*<br>☐ *No (go to Q14)*
10.	Do you live with this sexual partner?	☐ *Yes*<br>☐ *No*
11.	Does this partner have other sexual partners?	☐ *Yes*<br>☐ *No*<br>☐ *Unsure*
12.	Have you ever tested for HIV with this primary sexual partner?	☐ *Yes*<br>☐ *No*
13.	[If Q12==Yes] What was your primary sexual partner's HIV test result:	☐ *HIV-negative*<br>☐ *HIV-positive*<br>☐ *Unknown*<br>☐ *Prefer not to answer*
14.	**In the past 6 months,** have you had sex without a condom with a partner(s) of unknown or positive HIV status?	☐ *Yes*<br>☐ *No*<br>☐ *Prefer not to answer*
15.	**In the past 6 months**, have you been diagnosed with or treated for an STI?	☐ *Yes*<br>☐ *No*<br>☐ *Prefer not to answer*
16.	**In the past 6 months**, have you used emergency contraception?	☐ *Yes*<br>☐ *No*<br>☐ *Prefer not to answer*
17.	Have you previously used emergency contraception more than twice?	☐ *Yes*<br>☐ *No*<br>☐ *Prefer not to answer*
18.	**In the past 6 months,** have you used post-exposure prophylaxis (PEP) two or more times?	☐ *Yes*<br>☐ *No*

		☐ *Prefer not to answer*
19.	**<u>In the past 6 months</u>,** have you had sex under the influence of drugs or alcohol?	☐ *Yes*<br>☐ *No*<br>☐ *Prefer not to answer*
20.	**<u>In the past 6 months</u>,** did you engage in sex in exchange of money or other favors? (e.g., sell sex)	☐ *Yes*<br>☐ *No*<br>☐ *Prefer not to answer*
21.	How old were you when you first had sex?<br>*(Source: Cleland; Illustrative Questionnaire for Interview Surveys with Young People)*	|__|__| *age*<br>☐ *Don't know*<br>☐ *Prefer not to answer*
22.	Have you ever been pregnant? [No== skip to Q24]	☐ *Yes*<br>☐ *No*
23.	Number of living children?	|__||__|| *number* |__|__| *number*
24.	Are you using any of the following birth control methods? [check all that apply]	☐ *None (skip to Q25)*<br>☐ *Oral birth control pills*<br>☐ *Implants*<br>☐ *IUD*<br>☐ *Tubal Ligation/Hysterectomy*<br>☐ *Pregnant [skip to section 3]*<br>☐ *Injectable*<br>☐ *Emergency contraception*<br>☐ *Condoms*<br>☐ *Other, specify: ___________*
24a.	How do you access these birth control methods? [check all that apply]	☐ *Retail pharmacy*<br>☐ *Health facility*<br>☐ *Family planning clinic*<br>☐ *Friend/family member*<br>☐ *Sexual partner(s)*<br>☐ *Other:___________________*
25.	Are you (and your partner) currently trying to conceive a child?	☐ *Yes*<br>☐ *No*

3. Depression

The next set of questions are a list of problems that people can get. These questions are about how you have been feeling during the ***past two weeks****. For both items, please let me know if you have felt this or experienced this not at all; several days; more than half the days; or nearly every day*

Source: PHQ-2 Scale (Kroenke et al., Medical Care, 2003)

1.	<u>In the past two weeks</u>, how often have you had little interest or pleasure in doing things?	☐ *Not at all (0 days)*<br>☐ *Several days (1-7 days) [complete PHQ 9]*<br>☐ *More than half the days (8-12 days) [complete PHQ 9]*<br>☐ *Nearly every day (13-14 days) [complete PHQ 9]*
2.	<u>In the past two weeks,</u> how often have you been feeling down, depressed, or hopeless?	☐ *Not at all (0 days)*

		☐ *Several days (1-7 days) [complete PHQ 9]*<br>☐ *More than half the days (8-12 days) [complete PHQ 9]*<br>☐ *Nearly every day (13-14 days) [complete PHQ 9]*
**4. Relationship to Peers**<br>*Now we will ask you some questions about your peers and friends.*<br>*Source: Questions based on formative research*		
1.	Do you have close girlfriends (e.g., peers) whom you trust and confine in?	☐ *Yes*<br>☐ *No*
2.	[If Q1 == yes] How many girlfriends do you feel close to?	|__|__||__|__ *number*
3.	[If Q1 == yes] How many of your close girlfriends do you think might be at risk of HIV?	|__|__||__||__||*number*|__ *number*
4.	How much time do you typically spend with your peers each week (in an average week)?	☐ *Do not frequently spend time with peers*<br>☐ *1-2 days per week*<br>☐ *3-4 days per week*<br>☐ *5 or more days per week*<br>☐ *Other: ____________*
5.	What types of things do you typically discuss with your friends/peers? [check all that apply]	☐ *Family planning*<br>☐ *HIV prevention (e.g., condoms, PrEP, PEP)*<br>☐ *Sex and relationships*<br>☐ *Family and friendships*<br>☐ *Work and/or school*<br>☐ *Coronavirus (COVID-19)*<br>☐ *Other:_______________*
6.	How has the COVID-19 pandemic influenced <u>in-person interactions</u> with your peers?	☐ *Has not changed in-person interactions with peers*<br>☐ *Has decreased in-person interactions with peers*<br>☐ *Has increased in-person interactions with peers*
7.	How has the COVID-19 pandemic influenced your <u>virtual (e.g., phone calls, text messaging, etc.) interactions</u> with peers?	☐ *Has not changed virtual interactions with peers*<br>☐ *Has decreased virtual interactions with peers*<br>☐ *Has increased virtual interactions with peers*
**5, PrEP**		
**5a. PrEP use and adherence**<br>*Now I would like to ask you some questions about your PrEP use and adherence to PrEP.*<br>*Source: Modified Medication Adherence Scale (Wilson et al., AIDS & Behavior, 2016)*		
1.	When did you first start taking PrEP?	*(Y Y Y Y) - (M M)*

2.	Have you stopped and re-started PrEP since then?	☐ *Yes*<br>☐ *No*
3.	How did you first learn about PrEP?	☐ Online/internet<br>☐ From healthcare workers/ at a health facility<br>☐ Research study<br>☐ Community outreach programs<br>☐ Advertisements (posters, pamphlets, brochures, etc.)<br>☐ Friend/peer<br>☐ Family member<br>☐ Sexual partner(s)<br>☐ Other________________________
4.	In the past 30 days, how many days did you miss taking your dose of PrEP?	|__|__| *days (number 0-30)*
5.	In the past 30 days, how often did you take your PrEP in the way you were supposed to?	☐ *Never*<br>☐ *Rarely*<br>☐ *Sometimes*<br>☐ *Usually*<br>☐ *Almost always*<br>☐ *Always*
6.	In the past 30 days, how good of a job did you do at taking your PrEP in the way you were supposed to?	☐ *Very poor*<br>☐ *Poor*<br>☐ *Fair*<br>☐ *Good*<br>☐ *Very good*<br>☐ *Excellent*
7.	What strategies do you use to remember to take PrEP? [Check all that apply]	☐ *I do not use any strategies*<br>☐ *Alarm*<br>☐ *Reminders from peer(s)*<br>☐ *Reminders from family members*<br>☐ *Reminders from a sexual partner(s)*<br>☐ *Other: _____________*
8.	Do you know anyone else taking PrEP?	☐ *Yes*<br>☐ *No*
8a	[If Q8==Yes] Who do you know who is taking PrEP? [Check all that apply]	☐ *Sexual partner(s)*<br>☐ *Friend/peer(s)*<br>☐ *Family member(s)*<br>☐ *Other: ________________*
9.	Does anyone know you are taking PrEP?	☐ *Yes*<br>☐ *No*
9a	[If Q10== Yes] Who knows you are taking PrEP? [Check all that apply]	☐ *Sexual partner(s)*<br>☐ *Friend/peer(s)*<br>☐ *Family member(s)*<br>☐ *Other: ________________*

10.	Have you had any side effects after starting PrEP?	☐ *Yes*<br>☐ *No*
10a	[If Q12== yes] Which side effects did you experience after starting PrEP? [Check all that apply]	☐ *Diarrhea*<br>☐ *Nausea*<br>☐ *Headache*<br>☐ *Stomach ache*<br>☐ *Fatigue*<br>☐ *Other:*______________________
11.	Thinking into the future, for much longer do you think you will continue using PrEP?	|__|__||__|__ *months*
12.	*A new form of PrEP might be available soon, which is an injection (e.g., shot) you would have to take every 8 weeks.*<br>How interested would you be in this form of PrEP delivery?	☐ *Not at all interested*<br>☐ *Slightly interested*<br>☐ *Neutral*<br>☐ *Interested*<br>☐ *Very interested*
12a	[If Q15 = neutral, interested or very interested]: If this form of long-acting injectable PrEP were available now, what form of PrEP would you most prefer?	☐ *Long-acting injectable PrEP (with shots every 2 months)*<br>☐ *Daily oral PrEP (with refills every 3 months)*<br>☐ *Neither*<br>☐ *Don't know*
12b	[If Q15 = neutral, interested or very interested]: Where would you most prefer to access this long-acting injectable PrEP from?	☐ *Healthcare clinic*<br>☐ *Family planning clinic*<br>☐ *Retail pharmacy*<br>☐ *Other:* ________________
13	[If Q15 = neutral, interested or very interested]: How much would you be willing to pay for 1 shot of long-acting injectable PrEP (that provides 8 weeks, or 2 months, of protective coverage)?	|__|__||__|__||__|__||__|__|_|__*kSH*
**5b. PrEP Perceptions**<br>*We will now ask you a few questions about your thoughts and feelings about daily (oral) PrEP.*<br>*Source: Modified scales from HIVST and Pharmacy PrEP questionnaires (Ortblad)*		
1.	Do you think PrEP makes sex completely safe from HIV?	☐ *Yes*<br>☐ *No*<br>☐ *Unsure*
2.	How worried are you about getting HIV in the next 3 months?	☐ *Not worried*<br>☐ *Somewhat worried*<br>☐ *Very worried*
3.	Do you agree with the following statement: *"My future health depends on me taking PrEP"*?	☐ *Completely agree*<br>☐ *Agree*<br>☐ *Neutral*<br>☐ *Disagree*

		☐ *Completely disagree*
4.	Before you began PrEP, how worried were you about getting side effects from PrEP?	☐ *Not worried*<br>☐ *Somewhat worried*<br>☐ *Very worried*
**5c. PrEP Stigma**<br>*We are interested in understanding how you think people in your community generally feel about PrEP. I am going to read some statements, and I'd like you to indicate how much you agree or disagree with the statement. Your answer choices are completely agree; agree; neutral; disagree; completely disagree; or don't know.*<br>*SOURCE: Modified perceived stigma (Kaai, Sahara-J: Journal of Social Aspects of HIV/AIDS, 2012).*		
1.	**The first statement is:** *"People in my community think people who take PrEP are promiscuous and having casual sex".*	☐ *Completely agree*<br>☐ *Agree*<br>☐ *Neutral*<br>☐ *Disagree*<br>☐ *Completely disagree*<br>☐ *Don't know*
2.	**The second statement is:** "*People in my community think people who take PrEP are being responsible for their sexual health".*	*See responses above*
3.	**The third statement is:** *"People in my community think PrEP may not be safe for your health".*	*See responses above*
4.	**The last statement is:** *"People in my community think that taking PrEP means you have HIV".*	*See responses above*
**6. Self-Efficacy: Conducting Peer PrEP referral + HIVST**<br>*Now I would like to ask you some questions about your perceptions surrounding your confidence to deliver HIVST kits to peers and refer them to PrEP*		
**6a. Intervention element: HIVST**		
1.	How comfortable will you be approaching and talking to your peers about HIVST?	☐ *Very comfortable*<br>☐ *Comfortable*<br>☐ *Neutral*<br>☐ *Uncomfortable*<br>☐ *Very uncomfortable*
2.	How comfortable will you be giving HIVST kits to your peers?	*Same answers as above*
3.	How confident are you in your ability to assist your peers to successfully HIVST?	☐ *Very confident*<br>☐ *Fairly confident*<br>☐ *Neutral*<br>☐ *A little confident*<br>☐ *Not at all confident*
4.	How confident are you in your ability to assist your peers to interpret their HIVST results?	*Same answers as above*
5.	How confident are you in your ability to keep your peers' HIVST results private (i.e., confidential)?	*Same answers as above*

6.	How confident are you in your ability to help your peers link to HIV care, if they test HIV-positive?	*Same answers as above*
7.	How beneficial do you think HIVST will be to your peers?<br><br>[TFA: Perceived Effectiveness]	☐ *Very beneficial*<br>☐ *Somewhat beneficial*<br>☐ *Neutral*<br>☐ *A little beneficial*<br>☐ *Not at all beneficial*
**6b. Intervention Element: PrEP**		
1.	How comfortable will you be approaching and talking to your peers about PrEP?	☐ *Very comfortable*<br>☐ *Comfortable*<br>☐ *Neutral*<br>☐ *Uncomfortable*<br>☐ *Very uncomfortable*
2.	How comfortable will you be telling your peers that you use PrEP?	*Same answers as above*
3.	[If Q2 == Very uncomfortable; Uncomfortable] Why do you think you would feel uncomfortable telling your peers that you use PrEP? [check all that apply]	☐ *Perceived to engage in risk behaviors*<br>☐ *PrEP mistaken for anti-retroviral therapy treatment (i.e., HIV-positive status)*<br>☐ *Stigmatization of PrEP use*<br>☐ *Other:_____________________*<br>☐ *Prefer not to answer*
4.	How confident are you in your ability to link a peer to PrEP, if they test HIV-negative?	☐ *Very confident*<br>☐ *Fairly confident*<br>☐ *Neutral*<br>☐ *A little confident*<br>☐ *Not at all confident*
5.	How confident are you in your ability to a support a peer to take PrEP medication every day?	*Same answers as above*
6.	How might you support a peer to take PrEP medication every day? [check all that apply]	☐ *Calling a peer*<br>☐ *Sending reminders/text messages*<br>☐ *Reminding a peer in-person (i.e., at work or school)*<br>☐ *I would not support a peer to take PrEP every day*<br>☐ *Other:_____________________*
7.	How beneficial do you think PrEP will be to your peers?<br><br>[TFA: Perceived Effectiveness]	☐ *Very beneficial*<br>☐ *Somewhat beneficial*<br>☐ *Neutral*<br>☐ *A little beneficial*<br>☐ *Not at all beneficial*
**7. Acceptability of a peer PrEP referral + HIVST model**<br><br>*Now we are going to ask you some questions about the perceived acceptability of an intervention in which you will deliver HIVST kits to peers and refer peers to PrEP*<br><br>*Source: Modified Theoretical Framework of Acceptability (TFA) adapted from the Pharmacy PrEP study (Ortblad & Roche).*		

I'm going to read a series of statements, and I would like you to say how much you agree or disagree with each statement. Your choices are "completely disagree", "disagree", "neutral", "agree", "completely agree"

1.	**The first statement is:** *"I think I will like delivering HIVST kits to my peers and referring them to PrEP."*<br><br>[TFA: Affective Attitude]	☐ *Completely disagree*<br>☐ *Disagree*<br>☐ *Neutral*<br>☐ *Agree*<br>☐ *Completely Agree*
2.	**The second statement is:** *"I think it will be hard to deliver HIVST kits to my peers and refer them to PrEP. "*<br><br>[TFA: Burden]	*Same answers as above*
3.	**The third statement is:** *"I am confident that I will be able to carry-out all elements of the intervention successfully (e.g., HIVST delivery and peer PrEP referral)."*<br><br>[TFA: Self-Efficacy]	*Same answers as above*
4.	**The fourth statement is:** *"I am confident that I will be able to solve any problems that may arise while delivering this intervention (e.g., HIVST and peer PrEP referral)."*<br><br>[TFA: Self-Efficacy]	*Same answers as above*
5.	**The fifth statement is:** *"I think it will be acceptable for me to deliver HIVST kits to my peers and refer them to PrEP."*<br><br>[General Acceptability]	*Same answers as above*
6.	**The sixth statement is: "***I don't understand why I should deliver HIVST kits to my peers and refer them to PrEP"*<br><br>[TFA: Intervention coherence]	*Same answers as above*
7.	**The seventh statement is***: "It is clear to me how this intervention will help to reduce HIV among young women in my community"*<br><br>[TFA: Intervention coherence]	*Same answers as above*
8.	**The eight statement is:** *"Delivering HIVST kits to my peers and referring them to PrEP will likely interfere with my other priorities"*<br><br>[TFA: Opportunity Costs]	*Same answers as above*
9.	**The ninth statement is:** *"Delivering HIVST kits to my peers and referring them to PrEP will not create any moral or ethical consequences for me"*<br><br>[TFA: Ethicality]	*Same answers as above*
10.	**The tenth statement is:** "*Delivering HIVST kits to my peers and referring them to PrEP will help them to remain HIV-negative"*<br><br>[TFA: Perceived effectiveness]	*Same answers as above*

8. Human Papillomavirus (HPV)

Now I would like to ask you some questions about HPV.

Source: Modified Gaborone Children's Health Survey (DiAngi et al., PLoS One, 2011)

Source: Modified Cervical Cancer and Awareness Screening (Mingo et al., International Journal of Gynecological Cancer, 2012)

1.	Have you ever heard of Human Papillomavirus or HPV? *(HPV is different from HIV).*	☐ *Yes*<br>☐ *No*
2.	Have you heard of genital warts?	☐ *Yes*<br>☐ *No*
2a	Has a doctor or other medical professional ever told you that you have genital warts or HPV?	☐ *Yes*<br>☐ *No*<br>☐ *Prefer not to answer*
3	Have you ever heard of cancer of the cervix or cervical cancer?	☐ *Yes*<br>☐ *No*
3a	Has a doctor or other medical professional ever told you that you have cervical cancer?	☐ *Yes*<br>☐ *No*<br>☐ *Prefer not to answer*
3b	Do you know anyone who has been diagnosed or treated for cervical cancer?	☐ *Yes*<br>☐ *No*<br>☐ *Prefer not to answer*
3c	[If Q6==Yes] Who do you know that has been diagnosed or treated for cervical cancer? [check all that apply]	☐ *Family member*<br>☐ *Friend/peer*<br>☐ *Neighbor/community member*<br>☐ *Other:______________________*
An HPV vaccine is now available that protects against most genital warts and cervical cancer. Sometimes it's called the cervical cancer vaccine, HPV shot, Cervavix, or Gardasil.		
4.	Have you heard of the HPV vaccine before today?	☐ *Yes*<br>☐ *No*
5.	[if Q4= Yes] Have you received the HPV vaccine (any or all doses)?	☐ *Yes*<br>☐ *No*<br>☐ *Unsure*
5a.	[If Q4= NO; If Q5= no/unsure] Would you be interested in getting an HPV vaccine in the future?	☐ *Yes*<br>☐ *No*<br>☐ *Unsure*
5b.	[If Q5a= No; Unsure]: Why would you not be interested in getting the HPV vaccine? *[check all that apply]*	☐ *Don't know enough about the HPV vaccine*<br>☐ *Don't feel at risk of HPV*<br>☐ *Worried about side effects*<br>☐ *Concerns about HPV vaccine safety*<br>☐ *Concerns of stigma associated with the HPV vaccine*<br>☐ *Other:_________________*
6	[If Q5==Yes | Q5a==Yes]: If the HPV vaccine were available at all of the following locations, where would you most prefer to access the HPV vaccine?	☐ *Public clinic*<br>☐ *Private clinic*<br>☐ *Retail pharmacy*

		☐ *Online delivery*<br>☐ *I would not like an HPV vaccine*<br>☐ *Other:________*
7.	[If Q5==Yes | Q5a==Yes]: How comfortable would you feel receiving your HPV vaccine at your local retail pharmacy?	☐ *Very uncomfortable*<br>☐ *Uncomfortable*<br>☐ *Neutral*<br>☐ *Comfortable*<br>☐ *Very comfortable*
8.	[If Q5==Yes | Q5a==Yes]: How much might you be willing to pay to receive 1 dose of the HPV vaccine at your local retail pharmacy?	|__|__||__|__||__|__||__|__|_|__*kSH*

9. Extended Depression Screening (PHQ 9)

[This portion of the questionnaire will only appear for individuals who reported depression in the depression screening. Please collect the following information in a culturally appropriate way in the context of a counseling session and be prepared to offer referral to mental health services. This PHQ 9 will only include the 7 questions not previously asked in this questionnaire]

Source: PHQ 9 (Kroenke & Spitzer, 2002)

1.	In the past two weeks, how often have you had trouble falling asleep, staying asleep, or sleeping too much?	☐ *Not at all (0 days)*<br>☐ *Several days (1-7 days)*<br>☐ *More than half the days (8-12 days)*<br>☐ *Nearly every day (13-14 days)*
2.	In the past two weeks, how often have you felt tired or had little energy?	*Same answers as above*
3.	In the past two weeks, how often have you been bothered by poor appetite or overeating?	*Same answers as above*
4.	In the past two weeks, how often have you been feeling bad about yourself or that you're a failure and have let yourself or your family down?	*Same answers as above*
5.	In the past two weeks, how often have you had trouble concentrating on things, such as reading a newspaper or watching televisions?	*Same answers as above*
6.	In the past two weeks, how often have you been moving so slowly that other people could have noticed. Or the opposite-being so fidgety and restless that you have been moving around a lot more than usual?	*Same answers as above*
7.	In the past two weeks, how often have you been bothered by thoughts that you would be better off dead or hurting yourself in some way?	*Same answers as above*

10. Social harm report

[This portion of the questionnaire will only appear for individuals who reported any social harm in the demographics section. Please collect the following information in a culturally appropriate way in the context of a counseling session and be prepared to offer referral to GBV/IPV services if warranted.]

1.	In the **last 3 months,** was the participant ***verbally*** abused by anyone?	☐ *Yes*<br>☐ *No*

		☐ *Prefer not to answer*
1a.	[If Q1='Yes'] How often was the participant ***verbally*** abused (in the last 3 months)?	|__|__|__| *number of times*<br>☐ *Don't know*<br>☐ *Prefer not to answer*
1b	[If Q1='Yes'] Who was the participant ***verbally*** abused by? *[check all that apply]*	☐ *Family member*<br>☐ *Intimate partner(s)*<br>☐ *Neighbor/ community member*<br>☐ *Friend/peer*<br>☐ *Other:_____________*<br>☐ *Prefer not to answer*
2.	In the **last 3 months,** was the participant ***physically*** abused by anyone?	☐ *Yes*<br>☐ *No*<br>☐ *Prefer not to answer*
2a.	[If Q2='Yes'] How often was the participant ***physically*** abused (in the last 3 months)?	|__|__|__| *number of times*<br>☐ *Don't know*<br>☐ *Prefer not to answer*
2b.	[If Q2='Yes'] Who was the participant ***physically*** abused by (in the last 3 months) *[check all that apply]*	☐ *Family member*<br>☐ *Intimate partner(s)*<br>☐ *Neighbor/ community member*<br>☐ *Friend/peer*<br>☐ *Other:_____________*<br>☐ *Prefer not to answer*
3.	In the **last 3 months,** was the participant ***emotionally*** abused by anyone?	☐ *Yes*<br>☐ *No*<br>☐ *Prefer not to answer*
3a.	[If Q3='Yes'] How often was the participant ***emotionally*** abused (in the last 3 months)?	|__|__|__| *number of times*<br>☐ *Don't know*<br>☐ *Prefer not to answer*
3b	[If Q3='Yes'] Who was the participant ***emotionally*** abused by (in the last 3 months)? *[check all that apply]*	☐ *Family member*<br>☐ *Intimate partner(s)*<br>☐ *Neighbor/ community member*<br>☐ *Friend/peer*<br>☐ *Other:_____________*<br>☐ *Prefer not to answer*
4.	Was the participant abused by anyone in any other way (not fitting into the categories of verbal, physical, or emotional abuse)?	☐ *Yes*<br>☐ *No*<br>☐ *Prefer not to answer*
4a.	[If Q4='Yes'] Please concisely describe the social harm:	☐ *Prefer not to answer*<br><br>*Explain:* ____________________
4b.	[If Q4='Yes'] How often did this social harm occur in the last three months?	|__|__|__| *number of times*<br>☐ *Don't know*

		☐ *Prefer not to answer*
4c.	[If Q4=='Yes'] Who did the participant experience this social harm from?	☐ *Family member*<br>☐ *Intimate partner(s)*<br>☐ *Neighbor/ community member*<br>☐ *Friend/peer*<br>☐ *Other:*_____________<br>☐ *Prefer not to answer*

Questionnaire: Follow-Up (PrEP User/Peer Provider)

			CommCare QuestionID
This follow-up questionnaire is to be conducted by research staff following the implementation of the peer PrEP referral + HIVST model. This follow-up questionnaire has **8 sections**: 1) Demographics, 2) Sexual behaviors, 3) Depression screening, 5) PrEP use, perceptions, stigma, 6) Experiences: Peer PrEP referral + HIVST model, 7) Acceptability, appropriateness, and feasibility of a Peer PrEP referral + HIVST model, 8) Acceptability of Peer Delivered PrEP. Participants should do their best to answer all questions and let the researcher know if they feel uncomfortable or prefer not to answer any of the questions. *Please note the section numbers are purposeful.*			
**Section 0. Logistics**<br>*Part 0: Let start some with basic logistics*			*P2_S0_logistics*
	Participant ID:	|__|__|-|__|__|-|__|__|__||__|-|__|	*P2_PTID*
	Follow-up Questionnaire Completion Date	[Captured in CommCare]	*P2_S0_visit_date*
	Where is this questionnaire being conducted?	☐ *PHRD*<br>☐ *Other:___________________*	*P2_S0_setting*<br>*P2_S0_setting_other*
	(Optional) RA notes about this form:<br>Please do not provide any Personal Health Information in these notes. Please mention any issues with sPTIDs, PTIDs, dates, etc. Otherwise please skip this question.		*P2_S0_notes*
**Section 1. Demographics**<br>*First, I would like to ask you some basic questions about yourself.*			*P2_S1_demographics*
1.	What is your relationship status?	☐ Single, no partners<br>☐ Casual partner(s) only<br>☐ One primary partner<br>☐ One primary partner and casual partners<br>☐Other:____________________	*P2_S1_Q1*<br>*P2_S1_Q1_other*
2.	**In the last month**, have you been verbally, physically or emotionally abused by anyone?	☐ *Yes [Complete social harm report]*<br>☐ *No*<br>☐ *Prefer not to answer*	*P2_S1_Q2*
**Section 2. Sexual Behaviors**<br>*Now I would like to ask you some questions about your sexual behaviors.*<br>*Source: PrEP RAST, (DREAMS PrIYA, 2017)*			*P2_S2_sexual_behaviors*
1.	When did you last test for HIV before today?	________ *months*<br>☐ *Within the last month*	*P2_S2_Q1*
2.	What were the results of your last HIV test?	☐ *HIV-negative*<br>☐ *HIV-positive*<br>☐ *Unknown*<br>☐ *Prefer not to answer*	*P2_S2_Q2*
3.	**In the past two weeks**, how many times have you had sexual intercourse?	|__|__|__| *number of sex acts*<br>*(if 0, go to Q4)*<br>☐ *Prefer not to answer*	*P2_S2_Q3*

3a.	When you had sex **in the past two weeks**, how many times was a condom used?	||____|__| *number of times condom used*<br>☐ *Prefer not to answer*	*P2_S2_Q3a*
4.	**In the past three months**, how many individuals have you had sex with?	|__|__| *number of sexual partners (if 0 go to Q5)*<br>☐ *Prefer not to answer*	*P2_S2_Q4*
4a.	Of the individuals you have had sex with **in the past three months**, how many are new sexual partners?	|__|__| *number of new sexual partners*<br>☐ *Prefer not to answer*	*P2_S2_Q4a*
5.	Of your sexual partners, how many do you know their HIV status?	|__|__| *number of sexual partners*<br>☐ *N/A have no current sexual partners*<br>☐ *Don't know*<br>☐ *Prefer not to answer*	*P2_S2_Q5*
6.	Do you have a primary sexual partner?	☐ *Yes*<br>☐ *No (go to Q7)*	*P2_S2_Q6*
6a.	Is this the same a primary sexual partner as in the past month (i.e., since the last questionnaire)?	☐ *Yes*<br>☐ *No, this is a new primary partner*	*P2_S2_Q6a*
6b.	Do you live with this sexual partner?	☐ *Yes*<br>☐ *No*	*P2_S2_Q6b*
6c.	Does this partner have other sexual partners?	☐ *Yes*<br>☐ *No*<br>☐ *Unsure*	*P2_S2_Q6c*
6d.	Have you ever tested for HIV with this primary sexual partner?	☐ *Yes*<br>☐ *No*	*P2_S2_Q6d*
6e.	[If Q6d=="Yes"] What was your primary sexual partner's HIV test result?	☐ *HIV-negative*<br>☐ *HIV-positive*<br>☐ *Unknown*<br>☐ *Prefer not to answer*	*P2_S2_Q6e*
7.	**In the past 6 months**, have you had sex without a condom with a partner(s) of unknown or positive HIV status?	☐ *Yes*<br>☐ *No*<br>☐ *Prefer not to answer*	*P2_S2_Q7*
8.	**In the past 6 months**, have you been diagnosed with or treated for an STI?	☐ *Yes*<br>☐ *No*<br>☐ *Prefer not to answer*	*P2_S2_Q8*
9.	**In the past 6 months**, have you used emergency contraception?	☐ *Yes*<br>☐ *No*<br>☐ *Prefer not to answer*	*P2_S2_Q9*
10.	Have you previously used emergency contraception more than twice?	☐ *Yes*<br>☐ *No*<br>☐ *Prefer not to answer*	*P2_S2_Q10*
11.	**In the past 6 months,** have you used post-exposure prophylaxis (PEP) two or more times?	☐ *Yes*<br>☐ *No*<br>☐ *Prefer not to answer*	*P2_S2_Q11*
12.	**In the past 6 months**, have you had sex under the influence of drugs or alcohol?	☐ *Yes*<br>☐ *No*<br>☐ *Prefer not to answer*	*P2_S2_Q12*
13.	**In the past 6 months**, did you engage in sex in exchange of money or other favors? (e.g., sell sex)	☐ *Yes*<br>☐ *No*	*P2_S2_Q13*

		☐ *Prefer not to answer*	
14.	Have you recently become pregnant (i.e., in the past month) or are you currently pregnant?	☐ *Yes*<br>☐ *No*<br>☐ *Unsure [if unsure of current pregnancy- offer pregnancy test and counselling following survey]*	*P2_S2_Q14*<br>*P2_S2_Q14b_unsure*
15.	Are you using any of the following birth control methods? [check all that apply]	☐ *None*<br>☐ *Oral*<br>☐ *Implants*<br>☐ *IUD*<br>☐ *Tubal Ligation/Hysterectomy*<br>☐ *Pregnant [skip to section 3]*<br>☐ *Injectable*<br>☐ *Emergency contraception*<br>☐ *Condoms*<br>☐ *Other, specify: ___________*	*P2_S2_Q15*<br>*P2_S2_Q15_other*
16.	Are you (and your partner) currently trying to conceive a child?	☐ *Yes*<br>☐ *No*	*P2_S2_Q16*
**Section 3. Depression**<br>*The next set of questions are a list of problems that people can get. These questions are about how you have been feeling during the* ***past two weeks****. For both items, please let me know if you have felt this or experienced this not at all; several days; more than half the days; or nearly every day*<br>*Source: PHQ-2 Scale (Kroenke et al., Medical Care, 2003)*			*P2_S3_depression*
1.	**In the past two weeks**, how often have you been feeling little interest or pleasure in doing things?	☐ *Not at all (0 days)*<br>☐ *Several days (1-7 days) [complete PHQ 9]*<br>☐ *More than half the days (8-12 days) [complete PHQ 9]*<br>☐ *Nearly every day (13-14 days) [complete PHQ 9]*	*P2_S3_Q1*
2.	**In the past two weeks,** how often have you felt down, depressed, or hopeless?	☐ *Not at all (0 days)*<br>☐ *Several days (1-7 days) [complete PHQ 9]*<br>☐ *More than half the days (8-12 days) [complete PHQ 9]*<br>☐ *Nearly every day (13-14 days) [complete PHQ 9]*	*P2_S3_Q2*
**Section 5: PrEP use, perceptions, stigma**			*(Note: No section 4 in follow-up)*<br>*P2_S5_PrEP*
**5a. PrEP use and adherence**<br>*Now I would like to ask you some questions about your PrEP use and adherence to PrEP.*<br>*Source: Modified Medication Adherence Scale (Wilson et al., AIDS & Behavior, 2016)*			*P2_S5_PrEP_use_adherence*
1.	Have you stopped and re-started PrEP in the past month (i.e., since the last questionnaire)?	☐ *Yes*<br>☐ *No*	*P2_S5_Q1*
2.	Have you stopped(i.e., discontinued) PrEP during	☐ *Yes*	*P2_S5_Q2*

	the past month (i.e., since the last questionnaire)?	☐ *No*	
2a.	[If Q2=Yes] When did you stop (i.e., discontinue) using PrEP?	*(YYYY- MM- DD)*	*P2_S5_Q2a*
2b.	[If Q2 = Yes] Why have you stopped (i.e., discontinued) using PrEP? [check all that apply]	☐ *Experienced side effects*<br>☐ *Concerns about PrEP stigma*<br>☐ *No longer at HIV risk (e.g., relationship ended)*<br>☐ *Had a hard time using PrEP*<br>☐ *Had a hard time accessing PrEP*<br>☐ *Could not afford PrEP (e.g., transportation)*<br>☐ *Do not believe PrEP prevents HIV*<br>☐ *Other:________________*	*P2_S5_Q2b*<br>*P2_S5_Q2b_other*
3.	**In the past 30 days,** on how many days did you miss taking your dose of PrEP?	|__|__| *days (number 0-30)*	*P2_S5_Q3*
4.	**In the past 30 days**, how often did you take your PrEP in the way you were supposed to?	☐ *Never*<br>☐ *Rarely*<br>☐ *Sometimes*<br>☐ *Usually*<br>☐ *Almost always*<br>☐ *Always*	*P2_S5_Q4*
5.	**In the past 30 days,** how well do you think you took your PrEP in the way you were supposed to?	☐ *Very poor*<br>☐ *Poor*<br>☐ *Fair*<br>☐ *Good*<br>☐ *Very good*<br>☐ *Excellent*	*P2_S5_Q5*
6.	What strategies do you use to remember to take PrEP? [Check all that apply]	☐ *I do not use any strategies*<br>☐ *Alarm*<br>☐ *Reminders from peers*<br>☐ *Reminders from family members*<br>☐ *Reminders from sexual partner(s)*<br>☐ *Other: _______________*	*P2_S5_Q6*<br>*P2_S5_Q6_other*
7.	Do you know anyone else taking PrEP?	☐ *Yes*<br>☐ *No*	*P2_S5_Q7*
7a	[If Q7==Yes] Who do you know who is taking PrEP? [Check all that apply]	☐ *Sexual partner(s)*<br>☐ *Friend/peer(s)*<br>☐ *Family member(s)*<br>☐ *Other: _______________*	*P2_S5_Q7a*<br>*P2_S5_Q7a_other*
8	Does anyone know you are taking PrEP?	☐ *Yes*<br>☐ *No*	*P2_S5_Q8*
8a.	[If Q8== Yes] Who knows you are taking PrEP? [Check all that apply]	☐ *Sexual partner(s)*<br>☐ *Friend/peers*<br>☐ *Family members*	*P2_S5_Q8a*<br>*P2_S5_Q8a_other*

		☐ *Other: ______________*	
9.	Have you had any side effects after starting PrEP?	☐ *Yes*<br>☐ *No*	*P2_S5_Q9*
9a	[If Q9== yes] Which side effects did you experience after starting PrEP? [Check all that apply]	☐ *Diarrhea*<br>☐ *Nausea*<br>☐ *Headache*<br>☐ *Stomach ache*<br>☐ *Fatigue*<br>☐ *Other:____________________*	*P2_S5_Q9a*<br>*P2_S5_Q9a_other*
10.	Thinking into the future, how much longer do you think you will continue using PrEP?	|__|__||__|__ *months*	*P2_S5_Q10*
**5b. PrEP Perceptions**<br>*Source: Modified scales from HIVST and Pharmacy PrEP questionnaires (Ortblad)*			*P2_S5_PrEP_perceptions*
11.	Do you think PrEP makes sex completely safe from HIV?	☐ *Yes*<br>☐ *No*<br>☐ *Unsure*	*P2_S5_Q11*
12.	How worried are you about getting HIV **in the next 3 months**?	☐ *Not worried*<br>☐ *Somewhat worried*<br>☐ *Very worried*	*P2_S5_Q12*
13.	Do you agree with the following statement: *"My future health depends on me taking PrEP"*?	☐ *Completely agree*<br>☐ *Agree*<br>☐ *Neutral*<br>☐ *Disagree*<br>☐ *Completely disagree*	*P2_S5_Q13*
14.	**Before you began PrEP,** how worried were you about getting side effects from PrEP?	☐ *Not worried*<br>☐ *Somewhat worried*<br>☐ *Very worried*	*P2_S5_Q14*
**5c. PrEP Stigma**<br>*We are interested in understanding how you think people in your community generally feel about PrEP. I am going to read some statements, and I'd like you to indicate how much you agree or disagree with the statement. Your answer choices are completely agree; agree; neutral; disagree; or completely disagree; don't know.*<br><br>*SOURCE: Modified perceived stigma (Kaai, Sahara-J: Journal of Social Aspects of HIV/AIDS, 2012).*			*P2_S5_PrEP_stigma*
15.	**The first statement is:** *"People in my community think people who take PrEP are promiscuous and having casual sex."*	☐ *Completely agree*<br>☐ *Agree*<br>☐ *Neutral*<br>☐ *Disagree*<br>☐ *Completely disagree*<br>☐ *Don't know*	*P2_S5_Q15*
16.	**The second statement is:** *"People in my community think people who take PrEP are being responsible for their sexual health."*	☐ *Completely agree*<br>☐ *Agree*	*P2_S5_Q16*

		☐ *Neutral*<br>☐ *Disagree*<br>☐ *Completely disagree*<br>☐ *Don't know*	
17.	**The third statement is:** *"People in my community think PrEP may not be safe for your health."*	☐ *Completely agree*<br>☐ *Agree*<br>☐ *Neutral*<br>☐ *Disagree*<br>☐ *Completely disagree*<br>☐ *Don't know*	*P2_S5_Q17*
18.	**The last statement is:** *"People in my community think that taking PrEP means you have HIV."*	☐ *Completely agree*<br>☐ *Agree*<br>☐ *Neutral*<br>☐ *Disagree*<br>☐ *Completely disagree*<br>☐ *Don't know*	*P2_S5_Q18*

**Section 6. Experiences: Peer PrEP referral + HIVST**<br>*Following the delivery of HIVST kits to your peers, and referring them to PrEP, I would like to ask you some questions for feedback on the intervention. When we ask about peers in this section, we are only asking about the peers you referred to this study (max. 4 peers)*<br>*Source: Questions based on formative research stage*			*P2_S6_Experiences_PrEP_HIVST*
0.	How many peers did you deliver the peer PrEP referral + HIVST intervention to?	|__|__| *number (0-4 peers)*	
0a.	[If Q0>0] How many of these peers pinged the study staff to enroll in the pilot and be contacted at 1 month?	|__|__| *number (0-4 peers)*	
0b.	[If Q0!=Q0a] Why did these peers not ping the study staff? *(Check all that apply)*	☐ *Forgot to tell them*<br>☐ *Didn't have a phone*<br>☐ *Didn't feel comfortable sharing phone number*<br>☐ *Didn't want to participate in research activities*<br>☐ *Didn't want others to know they were participating in the study*<br>☐ *PrEP stigma*<br>☐ *Other*	
**6a. Experiences: HIVST delivery**			*P2_S6_HIVST*
1.	Did you **approach** and **talk** to your peers about HIVST?	☐ *Yes*<br>☐ *No*	*P2_S6_Q1*
1a.	[If Q1== Yes] How comfortable were you **approaching** and **talking** to peers about HIVST?	☐ *Very comfortable*<br>☐ *Comfortable*<br>☐ *Neutral*	*P2_S6_Q1a*

		☐ *Uncomfortable*<br>☐ *Very uncomfortable*	
1b.	[If very uncomfortable; uncomfortable] Why were you uncomfortable **approaching** and **talking** to your peers about HIVST? [check all that apply]	☐ *I did not know how to approach peers about HIVST*<br>☐ *I did not want to accuse my peers of having an HIV-positive status*<br>☐ *I do not have enough information/knowledge about conducting HIVST*<br>☐ *Prefer not to answer*<br>☐ *Other:____________________*	*P2_S6_Q1b*<br>*P2_S6_Q1b_other*
2.	How many HIVST kits did you **give** to peers? *[in total]*	|__|__| *number (0-8 HIVST kits)*	*P2_S6_Q2*
2a	[If Q2 = 0; after Q2a skip to Q10] Why did you **not give** your peers any HIVST kits? [check all that apply]	☐ *I did not know how to approach peers about HIVST*<br>☐ *Other peers, not included in this study, wanted HIVST kits*<br>☐ *Some peers wanted/needed more than 2 HIVST kits*<br>☐ *I do not have enough information/knowledge about conducting HIVST*<br>☐ *I had safety concerns delivering HIVST kits*<br>☐ *Prefer not to answer*<br>☐ *Other:__________________*	*P2_S6_Q2a*<br>*P2_S6_Q2a_other*
3.	To the best of your knowledge, how many of your peers **used** the HIV-self test kits you gave to them?	|__|__| *number (0-4 peers)*	*P2_S6_Q3*
4.	How comfortable were you **giving** HIVST kits to your peers?	☐ *Very comfortable*<br>☐ *Comfortable*<br>☐ *Neutral*<br>☐ *Uncomfortable*<br>☐ *Very uncomfortable*	*P2_S6_Q4*
4a	[If very uncomfortable; uncomfortable] Why were you uncomfortable **giving** HIV self-tests to your peers? [check all that apply]	☐ *I did not know how to approach peers about HIVST*<br>☐ *Other peers, not included in this study, wanted HIVST kits*<br>☐ *Some peers wanted/needed more than 2 HIVST kits*<br>☐ *I do not have enough information/knowledge about conducting HIVST*<br>☐ *I had safety concerns delivering HIVST kits*<br>☐ *Prefer not to answer*<br>☐ *Other:__________________*	*P2_S6_Q4a*<br>*P2_S6_Q4a_other*
5.	How often did you help your peers **use** their HIV self-tests?	☐ *Sometimes*<br>☐ *Always*<br>☐ *Never*	*P2_S6_Q5*
5a.	[If Q5= sometimes; always] How confident were you **assisting** your peers to successfully HIV self-test?	☐ *Very confident*	*P2_S6_Q5a*

		☐ *Fairly confident*<br>☐ *Neutral*<br>☐ *A little confident*<br>☐ *Not at all confident*	
5b.	[If not at all confident; a little confident] Why were you not confident **assisting** your peer to HIV self-test? [check all that apply]	☐ *I did not know how to help my peer(s) use their HIVST*<br>☐ *I had trouble reading and understanding the HIVST instructions*<br>☐ *Prefer not to answer*<br>☐ *Other:___________________*	*P2_S6_Q5b*<br>*P2_S6_Q5b_other*
6.	How often did you help your peers **interpret** their HIV self-test results?	☐ *Sometimes*<br>☐ *Always*<br>☐ *Never*	*P2_S6_Q6*
6a.	[If Q6== sometimes; always] How confident were you assisting your peers to **interpret** their HIV self-test results?	☐ *Very confident*<br>☐ *Confident*<br>☐ *Neutral*<br>☐ *A little confident*<br>☐ *Not at all confident*	*P2_S6_Q6a*
6b.	[If not at all confident; a little confident] Why were you not confident assisting your peer(s) to **interpret** their HIV self-test results? [check all that apply]	☐ *I did not know how to interpret HIVST results*<br>☐ *I feared my peer having an HIV positive status*<br>☐ *I feared my peer having an HIV negative status*<br>☐ *I did not know how to link my peers to care (i.e., HIV treatment or PrEP)*<br>☐ *Prefer not to answer*<br>☐ *Other:___________________*	*P2_S6_Q6b*<br>*P2_S6_Q6b_other*
7	Following HIV self-testing, how many *peers* shared (disclosed) their HIV statuses with you?	**|__|__|** *number (0-4 peers) (If 0, skip to Q10)*	*P2_S6_Q7*
7a	Have you shared your peers' HIV status(es) with anyone else?	☐ *Yes*<br>☐ *No*<br>☐ *Prefer not to answer*<br>☐ *Strongly agree*<br>☐ *Agree*<br>☐ *Neutral*<br>☐ *Disagree*<br>☐ *Strongly disagree*	*P2_S6_Q7a*
7b	[If Q7b==Yes] Who did you **share** your peer's HIV status(es) with? [check all that apply]	☐ *Your sexual partner(s)*<br>☐ *Your peer's sexual partner(s)*<br>☐ *Other friend/peers*<br>☐ *Your family members*<br>☐ *Your peer's family members*<br>☐ *Community member/neighbors*<br>☐ *Prefer not to answer*<br>☐ *Other: _______________*	*P2_S6_Q7b*<br>*P2_S6_Q7b_other*
8.	[If Q7>0] **Please do not tell me the name of your peer(s*).*** Did any of your peers test *negative* for HIV using the HIV self-test?	☐ *Yes*<br>☐ *No (Skip to Q9)*<br>☐ *Unsure (Skip to Q9)*	*P2_S6_Q8*

8a	[If Q8==Yes] How many of your peers tested negative for HIV using the HIV self-test?	|__|__| *number (0-4 peers)*	*P2_S6_Q8a*
8b	[If Q8a>0] How many of these peers were you able to successfully **link** to PrEP services?	|__|__| *number (0-4 peers)*	*P2_S6_Q8b*
8c	[If discrepancy between Q8a and Q8b] Why were you unable to **link** some peers to PrEP services?	*Explain:*___________________<br>☐ *Prefer not to answer*	*P2_S6_Q8c*
9.	[If Q7>0 and Q8 ne 4] **Please do not tell me the name of your peer(s).** Did any of your peers test *positive* for HIV using the HIV self-test?	☐ *Yes*<br>☐ *No (Skip to Q10)*<br>☐ *Unsure (Skip to Q10)*	*P2_S6_Q9*
9a	[If Q9==Yes] How many of your peers tested positive for HIV using the HIV self-test?	|__|__| *number (0-4 peers)*	*P2_S6_Q9a*
9b	[If Q9a>0] How many of these peers were you able to **link** to confirmatory testing and/or HIV treatment?	|__|__| *number (0-4 peers)*	*P2_S6_Q9b*
9c	[If Q9b>0] To the best of your knowledge, how many of the peers you **linked** to HIV treatment, started HIV treatment (e.g., ART)?	|__|__| *number (0-4 peers)*	*P2_S6_Q9c*
9d	[If discrepancy between Q9b and Q9c] Why were you unable to **link** some peers to confirmatory testing and/or HIV treatment?	*Explain:*___________________<br>☐ *Prefer not to answer*	*P2_S6_Q9d*
10.	How beneficial did you think HIVST is to your peers?<br>[TFA: Perceived Effectiveness]	☐*Very beneficial*<br>☐ *Somewhat beneficial*<br>☐ *Neutral*<br>☐ *A little beneficial*<br>☐ *Not at all beneficial*	*P2_S6_Q10*
11.	In the future, do you think your peers would be willing to pay for an HIV self-test kit?	☐ *Yes*<br>☐ *No*	*P2_S6_Q11*
11a	[If Q11== Yes] How much do you think your peers would be willing to pay for an HIV self-test kit?	|__|__||__|__||__|__||__|__|_|__*kSH*	*P2_S6_Q11a*
	Now I am going to show you a series of pictures with different possible HIV self-test results and am going to ask you to interpret each result. Possible test results include: "HIV positive", "HIV negative" or "Invalid" (e.g., failed test).<br>*[These next four images will be presented to participants]* *Source: HIVST Questionnaire (Ortblad)*<br>Image Citation:<br>Chembio Diagnostic Systems Inc. Sure Check HIV Self Test [Internet]. Medford, NY, United States: Chembio (2024). https://chembio.com/products/sure-check-hiv-self-test-international/. [Accessed August 13, 2024]		*P2_S6_HIVST_interpret*
12	How would you interpret the results of this self-test?	☐ *HIV-negative*<br>☐ *HIV-positive*<br>☐ *Invalid*	*P2_S6_Q12*

	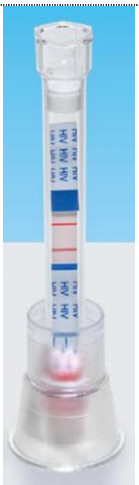	☐ *Don't know*	
13	How would you interpret the results of this self-test? 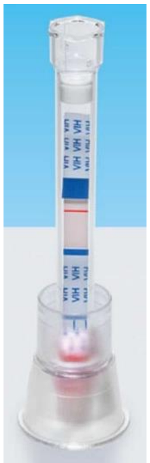	☐ *HIV-negative*<br>☐ *HIV-positive*<br>☐ *Invalid*<br>☐ *Don't know*	*P2_S6_Q13*
14	How would you interpret the results of this self-test? 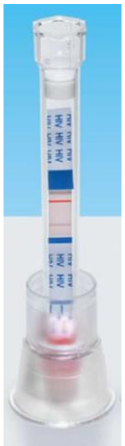	☐ *HIV-negative*<br>☐ *HIV-positive*<br>☐ *Invalid*<br>☐ *Don't know*	*P2_S6_Q14*
15	How would you interpret the results of this self-test?	☐ *HIV-negative*<br>☐ *HIV-positive*	*P2_S6_Q15*

	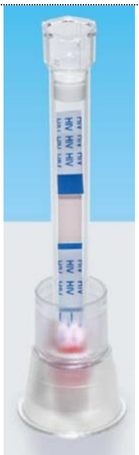	☐ *Invalid*<br>☐ *Don't know*	
**6b. Experiences: PrEP Referral**			
16.	Did you **approach** and **talk** to your peers about PrEP?	☐ *Yes*<br>☐ *No [skip to Q18]*	*P2_S6_Q16*
16a.	[If Q16==Yes] How comfortable were you **approaching** and **talking** to your peers about PrEP?	☐ *Very comfortable*<br>☐ *Comfortable*<br>☐ *Neutral*<br>☐ *Uncomfortable*<br>☐ *Very uncomfortable*	*P2_S6_Q16a*
16b.	[If very uncomfortable; uncomfortable]: Why were you uncomfortable **approaching** and **talking** to your peers about PrEP? [check all that apply]	☐ *I did not know how to approach peers about PrEP*<br>☐ *I did not want to accuse my peers of having an HIV positive status*<br>☐ *I do not have enough information/knowledge about PrEP*<br>☐ *Prefer not to answer*<br>☐ *Other:_____________________*	*P2_S6_Q16b*<br>*P2_S6_Q16b_other*
16c.	[If Q16==Yes] How many peer did you talk to about PrEP?	|__|__| *number (0-4 peers)*	
16d.	[If Q16c>0] How many of these peers did you give a PrEP study brochure?	|__|__| *number (0-4 peers)*	
17.	[If Q16==Yes] How often did you **tell** your peers about *your* PrEP use?	☐ *Sometimes*<br>☐ *Always*<br>☐ *Never*	*P2_S6_Q17*
17a.	[If Q17 == sometimes; always] How comfortable were you **telling** *your* peers that *you* use PrEP?	☐ *Very comfortable*<br>☐ *Comfortable*<br>☐ *Neutral*<br>☐ *Uncomfortable*<br>☐ *Very uncomfortable*	*P2_S6_Q17a*
17b.	[If very uncomfortable; uncomfortable] Why were you uncomfortable **telling** your peers that *you* use PrEP? [check all that apply]	☐ *Perceived to engage in risk behaviors*<br>☐ *PrEP mistaken for anti-retroviral therapy (i.e., HIV-positive status)*<br>☐ *Stigmatization of PrEP use*<br>☐ *Other:_____________________*	*P2_S6_Q17b*<br>*P2_S6_Q17b_other*
18.	How often did you **link** any peers to PrEP services?	☐ *Sometimes*	*P2_S6_Q18*

		☐ *Always*<br>☐ *Never*	
18a.	[If Q18= sometimes; always] How many peers did you **link** to PrEP services?	|__|__| *number (0-4 peers)*	*P2_S6_Q18a*
19.	How often did you **accompany** your peers to a clinic to start PrEP?	☐ *Sometimes*<br>☐ *Always*<br>☐ *Never*	*P2_S6_Q19*
19a.	[If Q19== sometimes; always] How many peers did you **accompany** to a clinic to start PrEP?	|__|__| *number (0-4 peers)*	*P2_S6_Q19a*
20.	[If Q18a>= 1 or Q19a >=1] To the best of your knowledge, how many of the peers you referred to PrEP services, started PrEP?	|__|__| *number (0-4 peers)*	*P2_S6_Q20*
21.	[If Q20>0] Did you **support** your peers to take their PrEP medication every day?	☐ *Yes*<br>☐ *No [skip to Q22]*	*P2_S6_Q21*
21a.	[If Q21==Yes] How many peers did you **support** to take PrEP medication every day?	|__|__| *number (0-4 peers)*	*P2_S6_Q21a*
21b.	[If Q21==Yes] How did you **support** your peers to take PrEP medication every day? [check all that apply]	☐ *By calling peers*<br>☐ *By sending reminders/text messages*<br>☐ *By reminding a peer in-person (i.e., at work or school)*<br>☐ *Prefer not to answer*<br>☐ *Other:________________*	*P2_S6_Q21b*<br>*P2_S6_Q21b_other*
22.	How beneficial do you think PrEP is to your peers?<br><br>[TFA: Perceived Effectiveness]	☐ *Very beneficial*<br>☐ *Somewhat beneficial*<br>☐ *Neutral*<br>☐ *A little beneficial*<br>☐ *Not at all beneficial*	*P2_S6_Q22*
**Section 7. Acceptability, Appropriateness, and Feasibility of Peer PrEP Referral + HIVST model**<br>*Now we are going to ask you some questions about how acceptable you found the intervention.*<br>*Source: Modified Theoretical Framework of Acceptability (TFA) adapted from the Pharmacy PrEP study (Ortblad & Roche).*			*P2_S7_acceptability_appropriateness_feasibility_PrEP_HIVST*<br><br>*P2_S7_acceptability_PrEP_HIVST_label*
I'm going to read a series of statements, and I would like you to say how much you agree or disagree with each statement. Your choices are "completely disagree", "disagree", "neutral", "agree", "completely agree"			*P2_S7_acceptability_PrEP_HIVST_instructions*
**Section 7a. Acceptability of a peer PrEP referral + HIVST model**			*P2_S7_acceptability*
1.	**The first statement is:** *"I liked delivering HIVST kits to my peers and referring them to PrEP (or HIV treatment)."*<br><br>[TFA: Affective Attitude]	☐ *Completely disagree*<br>☐ *Disagree*<br>☐ *Neutral*<br>☐ *Agree*<br>☐ *Completely agree*	*P2_S7_Q1*
2.	**The second statement is:** *"It was hard to deliver HIVST kits to my peers and refer them to PrEP (or HIV*	☐ *Completely disagree*	*P2_S7_Q2*

	treatment).”<br>[TFA: Burden]	☐ *Disagree*<br>☐ *Neutral*<br>☐ *Agree*<br>☐ *Completely agree*	
3.	**The third statement is:** *“I was confident in my ability to carry-out all elements of the intervention successfully (e.g., HIVST delivery and peer PrEP referral).”*<br>[TFA: Self-Efficacy]	☐ *Completely disagree*<br>☐ *Disagree*<br>☐ *Neutral*<br>☐ *Agree*<br>☐ *Completely agree*	*P2_S7_Q3*
4.	**The fourth statement is:** *“I was confident in my ability to solve any problems that arose while delivering this intervention (e.g., HIVST delivery and peer PrEP referral).”*<br>[TFA: Self-Efficacy]	☐ *Completely disagree*<br>☐ *Disagree*<br>☐ *Neutral*<br>☐ *Agree*<br>☐ *Completely agree*	*P2_S7_Q4*
5.	**The fifth statement is:** *“It was acceptable for me to deliver HIVST kits to my peers and refer them to PrEP (or HIV treatment).”*<br>[General Acceptability]	☐ *Completely disagree*<br>☐ *Disagree*<br>☐ *Neutral*<br>☐ *Agree*<br>☐ *Completely agree*	*P2_S7_Q5*
6.	**The sixth statement is: “***I didn’t understand why I should deliver HIVST kits to my peers and refer them to PrEP (or HIV treatment).”*<br>[TFA: Intervention coherence]	☐ *Completely disagree*<br>☐ *Disagree*<br>☐ *Neutral*<br>☐ *Agree*<br>☐ *Completely agree*	*P2_S7_Q6*
7.	**The seventh statement is***:* *“It is clear to me how this intervention helps to reduce HIV among young women in my community”*<br>[TFA: Intervention coherence]	☐ *Completely disagree*<br>☐ *Disagree*<br>☐ *Neutral*<br>☐ *Agree*<br>☐ *Completely agree*	*P2_S7_Q7*
8.	**The eight statement is:** *“Delivering HIVST kits to my peers and referring them to PrEP (or HIV treatment) interfered with my other priorities”*<br>[TFA: Opportunity Costs]	☐ *Completely disagree*<br>☐ *Disagree*<br>☐ *Neutral*<br>☐ *Agree*<br>☐ *Completely agree*	*P2_S7_Q8*
9.	**The ninth statement is:** *“Delivering HIVST kits to my peers and referring them to PrEP (or HIV treatment) did not create any moral or ethical consequences for me”*	☐ *Completely disagree*<br>☐ *Disagree*<br>☐ *Neutral*	*P2_S7_Q9*

	[TFA: Ethicality]	☐ *Agree*<br>☐ *Completely agree*	
10.	**The tenth statement is:** "*Delivering HIVST kits to my peers and referring them to PrEP helped them to remain HIV-negative*"<br>[TFA: Perceived effectiveness]	☐ *Completely disagree*<br>☐ *Disagree*<br>☐ *Neutral*<br>☐ *Agree*<br>☐ *Completely agree*	*P2_S7_Q10*
**7b. Appropriateness of a peer PrEP referral + HIVST model**			*P2_S7_appropriateness*
11.	This intervention (i.e., HIVST delivery with peer PrEP referral) seems fitting to adolescent girls and young women.	☐ *Completely disagree*<br>☐ *Disagree*<br>☐ *Neutral*<br>☐ *Agree*<br>☐ *Completely agree*	*P2_S7_Q11*
12.	This intervention (i.e., HIVST delivery with peer PrEP referral) seems like a good match to HIV prevention programs in Kenya.	☐ *Completely disagree*<br>☐ *Disagree*<br>☐ *Neutral*<br>☐ *Agree*<br>☐ *Completely agree*	*P2_S7_Q12*
**7c. Feasibility of a peer PrEP referral + HIVST model**			*P2_S7_feasibility*
13.	This peer delivered intervention (i.e., HIVST delivery and peer PrEP referral) seems possible to implement in Kenya.	☐ *Completely disagree*<br>☐ *Disagree*<br>☐ *Neutral*<br>☐ *Agree*<br>☐ *Completely agree*	*P2_S7_Q13*
14.	This intervention seems easy to implement in Kenya.	☐ *Completely disagree*<br>☐ *Disagree*<br>☐ *Neutral*<br>☐ *Agree*<br>☐ *Completely agree*	*P2_S7_Q14*
**Section 8. Acceptability of Peer-delivered PrEP**<br>*In the future, PrEP may be delivered by peers in the community. We would like to ask you a few questions about how acceptable you would find delivering or receiving PrEP from a peer. Please remember that we are now asking about peer delivered PrEP and not a peer referral to PrEP.*<br>*Source: Modified Theoretical Framework of Acceptability (TFA) adapted from the Pharmacy PrEP study (Ortblad & Roche).*			*P2_S8_acceptability*<br>*P2_S8_acceptability_label*
1.	**The first statement is:** *"I think I would like delivering PrEP directly to my peers"*<br>[TFA: Affective Attitude]	☐ *Completely disagree*<br>☐ *Disagree*<br>☐ *Neutral*<br>☐ *Agree*	*P2_S8_Q1*

		☐ *Completely Agree*	
2.	**The second statement is:** *"I think I would like receiving PrEP directly from my peers"*<br>[TFA: Affective Attitude]	☐ *Completely disagree*<br>☐ *Disagree*<br>☐ *Neutral*<br>☐ *Agree*<br>☐ *Completely Agree*	*P2_S8_Q2*
3.	**The third statement is:** *"I think it would be hard to deliver PrEP directly to my peers "*<br>[TFA: Burden]	☐ *Completely disagree*<br>☐ *Disagree*<br>☐ *Neutral*<br>☐ *Agree*<br>☐ *Completely Agree*	*P2_S8_Q3*
4.	**The fourth statement is:** *"I am confident that I would be able to deliver PrEP directly to my peers"*<br>[TFA: Self-Efficacy]	☐ *Completely disagree*<br>☐ *Disagree*<br>☐ *Neutral*<br>☐ *Agree*<br>☐ *Completely Agree*	*P2_S8_Q4*
5.	**The fifth statement is:** *"It is clear to me how the PrEP delivered by peers can reduce HIV among young women in my community"*<br>[TFA: Intervention coherence]	☐ *Completely disagree*<br>☐ *Disagree*<br>☐ *Neutral*<br>☐ *Agree*<br>☐ *Completely Agree*	*P2_S8_Q5*
6.	**The sixth statement is:** *"Delivering PrEP directly to my peers would likely interfere with my other priorities"*<br>[TFA: Opportunity Costs]	☐ *Completely disagree*<br>☐ *Disagree*<br>☐ *Neutral*<br>☐ *Agree*<br>☐ *Completely Agree*	*P2_S8_Q6*
7.	**The seventh statement is:** *"Delivering PrEP directly to my peers would not create any moral or ethical consequences for me"*<br>[TFA: Ethicality]	☐ *Completely disagree*<br>☐ *Disagree*<br>☐ *Neutral*<br>☐ *Agree*<br>☐ *Completely Agree*	*P2_S8_Q7*
8.	**The eighth statement is:** "*Delivering PrEP directly to my peers would help them to remain HIV-negative"*<br>[TFA: Perceived effectiveness]	☐ *Completely disagree*<br>☐ *Disagree*<br>☐ *Neutral*<br>☐ *Agree*<br>☐ *Completely Agree*	*P2_S8_Q8*

**Section 3 EX: Extended Depression Screening (PHQ 9)**<br>*[This portion of the questionnaire will only appear for individuals who reported depression in the depression screening. Please collect the following information in a culturally appropriate way in the context of a counseling session and be prepared to offer referral to mental health services. This PHQ 9 will only include the 7 questions not previously asked in this questionnaire]*<br>*Source: PHQ 9 (Kroenke & Spitzer, 2002)*			*P2_S3_depression_ex*
3.	**In the past two weeks**, how often have you had trouble falling asleep, staying asleep, or sleeping too much?	☐ *Not at all (0 days)*<br>☐ *Several days (1-7 days)*<br>☐ *More than half the days (8-12 days)*<br>☐ *Nearly every day (13-14 days)*	*P2_S3_Q3*
4.	**In the past two weeks,** how often have you felt tired or had little energy?	☐ *Not at all (0 days)*<br>☐ *Several days (1-7 days)*<br>☐ *More than half the days (8-12 day*<br>☐ *Nearly every day (13-14 days)*	*P2_S3_Q4*
5.	**In the past two weeks**, how often have you been bothered by poor appetite or overeating?	☐ *Not at all (0 days)*<br>☐ *Several days (1-7 days)*<br>☐ *More than half the days (8-12 day*<br>☐ *Nearly every day (13-14 days)*	*P2_S3_Q5*
6.	**In the past two weeks,** how often have you been feeling bad about yourself or that you're a failure and have let yourself or your family down?	☐ *Not at all (0 days)*<br>☐ *Several days (1-7 days)*<br>☐ *More than half the days (8-12 day*<br>☐ *Nearly every day (13-14 days)*	*P2_S3_Q6*
7.	**In the past two weeks**, how often have you had trouble concentrating on things, such as reading a newspaper or watching televisions?	☐ *Not at all (0 days)*<br>☐ *Several days (1-7 days)*<br>☐ *More than half the days (8-12 day*<br>☐ *Nearly every day (13-14 days)*	*P2_S3_Q7*
8.	**In the past two weeks**, how often have you been moving so slowly that other people could have noticed. Or the opposite- being so fidgety and restless that you have been moving around a lot more than usual?	☐ *Not at all (0 days)*<br>☐ *Several days (1-7 days)*<br>☐ *More than half the days (8-12 day*<br>☐ *Nearly every day (13-14 days)*	*P2_S3_Q8*
9.	**In the past two weeks**, how often have you been bothered by thoughts that you would be better off dead or hurting yourself in some way?	☐ *Not at all (0 days)*	*P2_S3_Q9*

		☐ *Several days (1-7 days)*<br>☐ *More than half the days (8-12 days)*<br>☐ *Nearly every day (13-14 days)*	
**SHR: Social harm report**<br>*[This portion of the questionnaire will only appear for individuals who reported any social harm above. Please collect the following information in a culturally appropriate way in the context of a counseling session and please be prepared to offer referral to GBV/IPV services if warranted.]*			*P2_SHR_social_harms*
1.	In the **last month** (i.e., since the last questionnaire), was the participant **verbally** abused by anyone?	☐ *Yes*<br>☐ *No*<br>☐ *Prefer not to answer*	*P2_SHR_Q1*
1a.	[If Q1='Yes'] How often was the participant **verbally** abused (in the last month)?	|__|__|__| *number of times*<br>☐ *Don't know*<br>☐ *Prefer not to answer*	*P2_SHR_Q1a*
1b.	[If Q1='Yes'] Who was the participant **verbally** abused by? [check all that apply]	☐ *Family member*<br>☐ *Intimate partner(s)*<br>☐ *Neighbor/ community member*<br>☐ *Friend/peer*<br>☐ *Other:____________*<br>☐ *Prefer not to answer*	*P2_SHR_Q1b*<br>*P2_SHR_Q1b_other*
1c.	[If Q1='Yes'] Was this **verbal** abuse a consequence of this study/ study activities?	☐ *Yes*<br>☐ *No*<br>☐ *Prefer not to answer*	*P2_SHR_Q1c*
1d.	[If Q1c='Yes'] Please concisely describe the **verbal abuse** resulting from this study/study activities.	☐ *Prefer not to answer*<br><br>*Explain:* ___________________	*P2_SHR_Q1d*
2.	In the **last month** (i.e., since the last questionnaire), was the participant **physically** abused by anyone?	☐ *Yes*<br>☐ *No*<br>☐ *Don't know*<br>☐ *Prefer not to answer*	*P2_SHR_Q2*
2a.	[If Q2='Yes'] How often was the participant **physically** abused (in the last month)?	|__|__|__| *number of times*<br>☐ *Prefer not to answer*	*P2_SHR_Q2a*
2b.	[If Q2='Yes'] Who was the participant **physically** abused by? [check all that apply]	☐ *Family member*<br>☐ *Intimate partner(s)*<br>☐ *Neighbor/ community member*<br>☐ *Friend/peer*<br>☐ *Other:____________*<br>☐ *Prefer not to answer*	*P2_SHR_Q2b*<br>*P2_SHR_Q2b_other*
2c.	[If Q2='Yes'] Was this **physical** abuse a consequence of this study/study activities?	☐ *Yes*<br>☐ *No*<br>☐ *Prefer not to answer*	*P2_SHR_Q2c*
2d	[If Q2c=Yes] Please concisely describe the **physical abuse** resulting from this study/study activities:	☐ *Prefer not to answer*	*P2_SHR_Q2d*

		Explain: ____________________	
3.	In the **last month** (i.e., since the last questionnaire), was the participant **emotionally** abused by anyone?	☐ *Yes*<br>☐ *No*<br>☐ *Prefer not to answer*	*P2_SHR_Q3*
3a.	[If Q3='Yes'] How often was the participant **emotionally** abused (in the past month)?	|__|__|__| *number of times*<br>☐ *Don't know*<br>☐ *Prefer not to answer*	*P2_SHR_Q3a*
3b.	[If Q3='Yes'] Who was the participant **emotionally** abused by? [check all that apply]	☐ *Family member*<br>☐ *Intimate partner(s)*<br>☐ *Neighbor/ community member*<br>☐ *Friend/peer*<br>☐ *Other:____________*<br>☐ *Prefer not to answer*	*P2_SHR_Q3b*<br>*P2_SHR_Q3b_other*
3c.	[If Q3='Yes'] Was this **emotional** abuse a consequence of this study/study activities?	☐ *Yes*<br>☐ *No*<br>☐ *Prefer not to answer*	*P2_SHR_Q3c*
3d.	[If Q3c= Yes] Please concisely describe the **emotional abuse** resulting from this study/study activities:	☐ *Prefer not to answer*<br><br>*Explain: ____________________*	*P2_SHR_Q3d*
4.	Was the participant abused by anyone in any other way (not fitting into the categories of verbal, physical, or emotional abuse)?	☐ *Yes (If Yes, describe in Q4a)*<br>☐ *No*<br>☐ *Prefer not to answer*	*P2_SHR_Q4*
4a.	Please concisely describe the social harm:	☐ *Prefer not to answer*<br><br>*Explain: ____________________*	*P2_SHR_Q4a*
4b	[If Q4='Yes'] How often did this social harm occur in the last month?	|__|__|__| *number of times*<br>☐ *Don't know*<br>☐ *Prefer not to answer*	*P2_SHR_Q4b*
4c.	[If Q4=='Yes'] Who did the participant experience this social harm from? [check all that apply]	☐ *Family member*<br>☐ *Intimate partner(s)*<br>☐ *Neighbor/ community member*<br>☐ *Friend/peer*<br>☐ *Other:____________*<br>☐ *Prefer not to answer*	*P2_SHR_Q4c*<br><br>*P2_SHR_Q4c_other*
4d.	[If Q4=='Yes'] Was this social harm a consequence of the study/study activities?	☐ *Yes*<br>☐ *No*<br>☐ *Prefer not to answer*	*P2_SHR_Q4d*

IRO REC'D 07/12/2022

Questionnaire: Follow-up (Peer Client)

This follow-up questionnaire is to be conducted by research staff. This questionnaire has **9 sections**: 1) Demographics, 2) Sexual behaviors, 3) Depression screening, 4) Relationships to peers, 5) Feedback: Peer PrEP referral + HIVST model, 6) PrEP use, perceptions, stigma, 7) Acceptability, appropriateness, and feasibility of a peer PrEP referral + HIVST model, 8) Acceptability of peer delivered PrEP 9) HPV. Participants should do their best to answer all questions and let the researcher know if they feel uncomfortable or prefer not to answer any of the questions.	*CommCare QuestionID*<br>*C2_introduction*

**Section 0. Enrollment and Logistics**<br>Please complete this form for all eligible participants only AFTER they have completed written informed consent.<br>*Let's start some with basic logistics*			*C2_S0_Enrollment_logistics_label*<br>*C2_S0_E_instructions*
	Participant's screening number<br>**Screening sPTID entry instructions:** PP-XXX<br>Please do not enter hyphens.<br>P= (01) peer provider, (02) peer client; XXX = unique screening ID - e.g., 001, 002, 003<br>***Make sure this matches that entered in the screening form for this participant***	|__|__|-|__|__|__|	*C2_E_sPTID*<br>*C2_E_sPTID_c*
	Did the participant meet all eligibility criteria?	☐ *Yes*<br>☐ *No [If No, do not proceed with questionnaire]*	*C2_E_eligible*<br>*C2_E_not_eligible*
	[If eligible==Yes] Date participant met eligibility criteria:	[Captured in CommCare]	*C2_E_eligible_date*
	[If eligible==Yes] Participant ID:	|__|__|-|__|__|-|__|__|__||__|-|__|	*C2_PTID*<br>*C2_PTID_c*
	[If eligible==Yes] What is the PTID of the peer provider who recruited this peer?	|__|__|-|__|__|-|__|__|__||__|-|__|	*C2_PP_PTID*
	[If eligible==Yes] Follow-up Questionnaire Completion Date	[Captured in CommCare]	*C2_S0_visit_date*
	[If eligible==Yes] Where is this questionnaire being conducted?	☐ *PHRD research clinic*<br>☐ *Other:__________*	*C2_S0_setting*<br>*C2_S0_setting_other*
	(Optional) RA notes about this form:<br>Please do not provide any Personal Health Information in these notes. Please mention any issues with sPTIDs, PTIDs, dates, etc. Otherwise please skip this question.		*C2_S0_notes*
**Section 1. Demographics**<br>*First, I would like to ask you some basic questions about yourself.*			*C2_S1_demographics*

FHCRC IRB Approval
AUG 11 2022
Document Released Date

1.	Age:	|__|__| *number of years*	*C2_S1_Q1*
2.	How many years of school have you completed? [Do not count repeat levels.]	|__|__| *number of years*<br>☐ *Prefer not to answer*	*C2_S1_Q2*
3.	Are you currently enrolled in school?	☐ *Yes*<br>☐ *No*	*C2_S1_Q3*
4.	What is your relationship status?	☐ Single, no partners<br>☐ Casual partner(s) only<br>☐ One primary partner<br>☐ One primary partner and casual partners<br>☐ Other______________________	*C2_S1_Q4*<br>*C2_S1_Q4_other*
5.	What is your monthly income *(average of the last 3 months)?*	|__|__||__|__||__|__||__|__|_|__*kSH*<br>☐ *Prefer not to answer*	*C2_S1_Q5*
6.	What is your main source of income *(in the past year)?*	☐ No income<br>☐ Laborer/semi-skilled<br>☐ Trade/Sales<br>☐ Student<br>☐ Professional<br>☐ Farming/animal raising<br>☐ Sex Worker<br>☐ Other:____________<br>☐ Prefer not to answer	*C2_S1_Q6*<br>*C2_S1_Q6_other*
7.	How often did you have 4 or more drinks of alcohol (e.g., the equivalent of 4 regular beer bottles) on a single occasion in the past year?<br>*(Source: M SASQ- Public Health England, 2020)*	☐ Never<br>☐ Less than monthly<br>☐ Monthly<br>☐ Weekly<br>☐ Daily or almost daily	*C2_S1_Q7*
8.	How many times in the past year have you used an illegal drug or used a prescription medication for non-medical reasons (e.g., because of the experience or feeling it caused)?<br>*(Source: SIP-DU; Smith et al., Archives of Internal Medicine, 2010)*<br>*(Source: SISQ, McNeely et al., Journal of General Internal Medicine, 2015)*	|__|__| *number [enter 0 if never]*<br>☐ *Prefer not to answer*	*C2_S1_Q8*
9.	In the **last three months**, have you been verbally, physically or emotionally abused by anyone?	☐ *Yes [Complete social harm report]*<br>☐ *No*<br>☐ *Prefer not to answer*	*C2_S1_Q9*
**Section 2. Sexual Behaviors**<br>*Now I would like to ask you some questions about your sexual behaviors.*<br>*Source: PrEP RAST, (DREAMS PrIYA, 2017)*			*C2_S2_sexual_behaviors*
1.	When did you last test for HIV before today?	_______ *months*<br>☐ *Within the last month*<br>☐ *Don't know*	*C2_S2_Q1*

		☐ *I have never tested for HIV (skip to Q5)*	
2.	What were the results of your last HIV test?	☐ *HIV-negative*<br>☐ *HIV-positive*<br>☐ *Unknown*<br>☐ *Prefer not to answer*	*C2_S2_Q2*
3.	How do you typically test for HIV?	☐ *Clinic-based: rapid testing*<br>☐ *Pharmacy-based (chemist): rapid testing*<br>☐ *Pharmacy-based (chemist): self-test (assisted/unassisted)*<br>☐ *Home-based: self-test*<br>☐ *Other: _______________*	*C2_S2_Q3*<br>*C2_S2_Q3_other*
4.	Who supports you when you go for HIV testing? [check all that apply]	☐ *No one*<br>☐ *Community member*<br>☐ *Family member*<br>☐ *Sexual partner(s)*<br>☐ *Friend/peer*<br>☐ *Other: _______________*	*C2_S2_Q4*<br>*C2_S2_Q4_other*
5.	**In the past two weeks,** how many times have you had sexual intercourse?	__||__|__| *number of sex acts*<br>*(if 0, go to Q6)*<br>☐ *Prefer not to answer*	*C2_S2_Q5*
5a.	When you had sex **in the past two weeks,** how many times was a condom used?	__|__|__| *number of times condom used*<br>☐ *Prefer not to answer*	*C2_S2_Q5a*
6.	**In the past three months**, how many individuals have you had sex with?	|__|__| *number of sexual partners*<br>☐ *Prefer not to answer*	*C2_S2_Q6*
6a.	Of the individuals you had sex with **in the past three months**, how many are new sexual partners?	|__|__| *number of new sexual partners*<br>☐ *Prefer not to answer*	*C2_S2_Q6a*
7.	Of your sexual partners, how many do you know their HIV status?	|__|__| *number of sexual partners*<br>☐ *N/A have no current sexual partners*<br>☐ *Don't know*<br>☐ *Prefer not to answer*	*C2_S2_Q7*
8.	Do you have a primary sexual partner?	☐ *Yes*<br>☐ *No (if no go to Q9)*	*C2_S2_Q8*
8a.	Do you live with this sexual partner?	☐ *Yes*<br>☐ *No*	*C2_S2_Q8a*
8b.	Does this partner have other sexual partners?	☐ *Yes*<br>☐ *No*<br>☐ *Unsure*	*C2_S2_Q8b*
8c.	Have you ever tested for HIV with this primary sexual partner?	☐ *Yes*<br>☐ *No*	*C2_S2_Q8c*
8d.	[If Q8c==Yes] What was your primary sexual partner's HIV test result?	☐ *HIV-negative*<br>☐ *HIV-positive*<br>☐ *Unknown*<br>☐ *Prefer not to answer*	*C2_S2_Q8d*

9.	**In the past 6 months**, have you had sex without a condom with a partner(s) of unknown or positive HIV status?	☐ *Yes*<br>☐ *No*<br>☐ *Prefer not to answer*	*C2_S2_Q9*
10.	**In the past 6 months**, have you been diagnosed with or treated for an STI?	☐ *Yes*<br>☐ *No*<br>☐ *Prefer not to answer*	*C2_S2_Q10*
11.	**In the past 6 months**, have you used emergency contraception?	☐ *Yes*<br>☐ *No*<br>☐ *Prefer not to answer*	*C2_S2_Q11*
12.	Have you previously used emergency contraception more than twice?	☐ *Yes*<br>☐ *No*<br>☐ *Prefer not to answer*	*C2_S2_Q12*
13.	**In the past 6 months,** have you used post-exposure prophylaxis (PEP) two or more times?	☐ *Yes*<br>☐ *No*<br>☐ *Prefer not to answer*	*C2_S2_Q13*
14.	**In the past 6 months**, have you had sex under the influence of drugs or alcohol?	☐ *Yes*<br>☐ *No*<br>☐ *Prefer not to answer*	*C2_S2_Q14*
15.	**In the past 6 months,** did you engage in sex in exchange of money or other favors? (e.g., sell sex)	☐ *Yes*<br>☐ *No*<br>☐ *Prefer not to answer*	*C2_S2_Q15*
16.	How old were you when you first had sex?<br>*(Source: Cleland; Illustrative Questionnaire for Interview Surveys with Young People)*	|__|__| *age*<br>☐ *N/A I have never had sex*<br>☐ *Don't know*<br>☐ *Prefer not to answer*	*C2_S2_Q16*
17.	Have you ever been pregnant? [No== skip to Q18]	☐ *Yes*<br>☐ *No*<br>☐ Prefer not to answer	*C2_S2_Q17*
17a.	Number of living children?	|__|__| number<br>☐ Prefer not to answer	*C2_S2_Q17a*
18.	Are you using any of the following birth control methods? [check all that apply]	☐ *None (Skip to Q19)*<br>☐ *Oral birth control pills*<br>☐ *Implants*<br>☐ *IUD*<br>☐ *Tubal Ligation/Hysterectomy*<br>☐ *Pregnant [skip to Section 3]*<br>☐ *Injectable contraception*<br>☐ *Emergency contraception*<br>☐ *Condoms*<br>☐ *Other, specify: ____________*	*C2_S2_Q18*<br>*C2_S2_Q18_other*
18a.	Where are you accessing these birth control methods? [check all that apply]	☐ *Retail pharmacy*<br>☐ *Health facility*<br>☐ *Family planning clinic*<br>☐ *Friend/family member*<br>☐ *Sexual partner(s)*<br>☐ *Other:___________________*	*C2_S2_Q18a*<br>*C2_S2_Q18a_other*

19.	Are you (and your partner) currently trying to conceive a child?	☐ *Yes*<br>☐ *No*	*C2_S2_Q19*
**Section 3. Depression**<br>*The next set of questions are a list of problems that people can get. These questions are about how you have been feeling during the **past two weeks**. For both items, please let me know if you have felt this or experienced this not at all; several days; more than half the days; or nearly every day*<br>*Source: PHQ-2 Scale (Kroenke et al., Medical Care, 2003)*			*C2_S3_depression*
1.	**In the past two weeks,** how often have you had little interest or pleasure in doing things?	☐ *Not at all (0 days)*<br>☐ *Several days (1-7 days) [complete PHQ 9]*<br>☐ *More than half the days (8-12 days) [complete PHQ 9]*<br>☐ *Nearly every day (13-14 days) [complete PHQ 9]*	*C2_S3_Q1*
2.	**In the past two weeks,** how often have you been feeling down, depressed, or hopeless?	☐ *Not at all (0 days)*<br>☐ *Several days (1-7 days) [complete PHQ 9]*<br>☐ *More than half the days (8-12 days) [complete PHQ 9]*<br>☐ *Nearly every day (13-14 days) [complete PHQ 9]*	*C2_S3_Q2*
**Section 4. Relationship to Peers**<br>*Now we will ask you some questions about your peers and friends.*<br>*Source: Questions based on formative research*			*C2_S4_peer_relationship*
1.	Do you have close girlfriends (e.g., peers) whom you trust and confide in?	☐ *Yes*<br>☐ *No*	*C2_S4_Q1*
1a.	[If Q1 == yes] How many girlfriends do you feel close to?	|__|__||__|__ *number*	*C2_S4_Q1a*
1b.	[If Q1 == yes] How many of your close girlfriends do you think might be at risk of HIV?	|__|__||__|__ *number*	*C2_S4_Q1b*
2.	How much time do you typically spend with your peers each week (*in an average week*)?	☐ *Do not frequently spend time with peers*<br>☐ *1-2 days per week*<br>☐ *3-4 days per week*<br>☐ *5 or more days per week*<br>☐ *Other: ____________*	*C2_S4_Q2*<br>*C2_S4_Q2_other*
3.	What types of things do you typically discuss with your friends/peers? [check all that apply]	☐ *Family planning*<br>☐ *HIV prevention (e.g., condoms, PrEP, PEP)*<br>☐ *Sex and relationships*<br>☐ *Family and friendships*<br>☐ *Work and/or school*<br>☐ *Coronavirus (COVID-19)*<br>☐ *Other:_______________*	*C2_S4_Q3*<br>*C2_S4_Q3_other*

4.	How has the COVID-19 pandemic influenced **in-person interactions** with your peers?	☐ *Has not changed in-person interactions with peers*<br>☐ *Has decreased in-person interactions with peers*<br>☐ *Has increased in-person interactions with peers*	*C2_S4_Q4*
5.	How has the COVID-19 pandemic influenced your **virtual interactions** (e.g., phone calls, text messaging, etc.) with peers?	☐ *Has not changed virtual interactions with peers*<br>☐ *Has decreased virtual interactions with peers*<br>☐ *Has increased virtual interactions with peers*	*C2_S4_Q5*
6.	How do you know the peer that delivered the intervention to you (e.g., delivered the HIV self-test and referred you to PrEP or HIV care)? *(select the primary way you know them)*	☐ *From school*<br>☐ *From work*<br>☐ *Through friends*<br>☐ *Through family*<br>☐ *Through sexual partner(s)*<br>☐ *Other:__________________*	*C2_S4_Q6*<br>*C2_S4_Q6_other*
7.	How long have you known the peer that delivered the intervention to you (e.g., delivered the HIV self-test and referred you to PrEP/ HIV care)?	|__|__||__|__ *months*	*C2_S4_Q7*
8.	How close of a peer would you consider the peer that delivered this intervention to you (e.g., delivered the HIV self-test and referred you to PrEP/HIV care)?	☐ *Very close friend*<br>☐ *Close friend*<br>☐ *Somewhat close friend*<br>☐ *Not close friend*<br>☐ *Very distant friend*	*C2_S4_Q8*
9.	How trustworthy would you consider the peer that delivered this intervention to you (e.g., delivered the HIV self-test and referred you to PrEP/HIV care)?	☐ *Very trustworthy*<br>☐ *Trustworthy*<br>☐ *No opinion*<br>☐ *Untrustworthy*<br>☐ *Very untrustworthy*	*C2_S4_Q9*
**Section 5. Feedback: Peer PrEP referral + HIVST**<br>*Now I would like to ask you some questions about your perceptions about this research study - the peer PrEP referral + HIVST model. When we ask about your peer in this section, we are referring to the peer who referred you to this research study.*<br>*Source: Questions based on formative research stage*			*C2_S5_Feedback_PrEP_HIVST*
**5a. Feedback: HIVST**			*C2_S5_HIVST*
1.	Did your peer **approach** and **talk** to you about *HIVST*?	☐ *Yes*<br>☐ *No (Skip to Q2)*	*C2_S5_Q1*
1a.	[If Q1== Yes] How comfortable did you feel being **approached** and **talked** to about HIVST by your peer?	☐ *Very comfortable*<br>☐ *Comfortable*<br>☐ *Neutral*<br>☐ *Uncomfortable*<br>☐ *Very uncomfortable*	*C2_S5_Q1a*

1b.	[If very uncomfortable; uncomfortable] Why were you uncomfortable being **approached** and **talked** to about HIVST by your peer? [check all that apply]	☐ *They did not approach me appropriately*<br>☐ *I felt my peer accused me of being HIV positive*<br>☐ *I do not feel that my peer had enough information/knowledge about conducting HIVST*<br>☐ *I do not feel that I had enough information/knowledge about conducting HIVST*<br>☐ *Prefer not to answer*<br>☐ *Other:___________________*	*C2_S5_Q1b*<br>*C2_S5_Q1b_other*
2.	How many HIV self-test kits did you **receive** from your peer?	|__|__| *number (if 0 skip to Q14)*<br>*If participant reports "0" HIVST kits, please confirm this number is correct before proceeding with questionnaire.*	*C2_S5_Q2*<br>*C2_S5_2a_c2*
3.	[If Q2=>1 HIVST kits How many of these tests did you **use** to test *yourself* for HIV?	|__|__| *number (if 0 skip to Q8)*	*C2_S5_Q3*
4.	[If Q3=>1 HIVST kits] When did you first test yourself for HIV using the self-test your peer gave you?	*YYYY-MM-DD*	*C2_S5_Q4*
4a.	[If Q4 ne 0] What were the results of your first HIVST your peer gave to you?	☐ *HIV-negative*<br>☐ *HIV-positive (skip to Q7)*<br>☐ *Unsure*<br>☐ *Prefer not to answer*	*C2_S5_Q4a*
5.	[If Q3= ≥ 2 HIVST kits] When did you use the second HIV self-test your peer gave to you to test for HIV?	*YYYY-MM-DD*	*C2_S5_Q5*
5a.	What were the results of your second HIVST your peer gave to you?	☐ *HIV-negative*<br>☐ *HIV-positive (skip to Q7)*<br>☐ *Unsure*<br>☐ *Prefer not to answer*	*C2_S5_Q5a*
6.	[If Q4a or Q5a = HIV-negative] Were you linked to PrEP?	☐ *Yes*<br>☐ *No*	*C2_S5_Q6*
6a.	[If Q6=No] Why were you not linked to PrEP?	*Explain:_____________________*<br>☐ *Prefer not to answer*	*C2_S5_Q6a*
7.	[If Q4a or Q5a = HIV-positive] Were you linked to *confirmatory HIV testing?*	☐ *Yes*<br>☐ *No*	*C2_S5_Q7*
7a.	[If Q7 = Yes] Were you eligible to begin *antiretroviral therapies* (ARTs) (e.g., HIV treatment)?	☐ *Yes*<br>☐ *No*	*C2_S5_Q7a*
7b.	[If Q7a = Yes] Have you begun taking *antiretroviral therapies* (ARTs) (e.g., HIV treatment)?	☐ *Yes*<br>☐ *No*	*C2_S5_Q7b*
7c.	[If Q7b = No] Why have you not begun taking *antiretroviral therapies* (ARTs) (e.g., HIV treatment)	*Explain:_____________________*<br>*(participants who are HIV-positive and have not yet begun ARTs should be counselled in a culturally-appropriate manner following the questionnaire as per the standard of care)*<br>☐ *Prefer not to answer*	*C2_S5_Q7c*

8.	[If Q3< Q2 or Q3=0] What did you do with the HIV self-test(s) you *did not use* to test yourself for HIV? [check all that apply]	☐ *Still have the HIVST with me*<br>☐ *Gave to a friend/peer*<br>☐ *Gave to a sexual partner*<br>☐ *Gave to a family member*<br>☐ *Other:__________________*	*C2_S5_Q8*<br>*C2_S5_Q8_other*
9.	[If Q2>0] How comfortable were you with your peer **giving** you HIV self-tests?	☐ *Very comfortable*<br>☐ *Comfortable*<br>☐ *Neutral*<br>☐ *Uncomfortable*<br>☐ *Very uncomfortable*	*C2_S5_Q9*
9a.	[If very uncomfortable; uncomfortable] Why were you uncomfortable with your peer **giving** you HIV self-testing kits? [check all that apply]	☐ *My other peers, not included in this study, wanted HIVST kits*<br>☐ *I wanted/needed more than 2 HIVST kits*<br>☐ *I do not feel that my peer had enough information/knowledge about conducting HIVST*<br>☐ *I do not feel that I had enough information/knowledge about conducting HIVST*<br>☐ *I had safety concerns owning HIVST kits*<br>☐ *Prefer not to answer*<br>☐ *Other:___________________*	*C2_S5_Q9a*<br>*C2_S5_Q9a_other*
10.	[If Q3>0] Did your peer help you to **use** your HIV self-tests?	☐ *Yes*<br>☐ *No*	*C2_S5_Q10*
10a.	[If Q10 ==Yes] How comfortable were you with your peer helping you **use** your HIV self-test?	☐ *Very comfortable*<br>☐ *Comfortable*<br>☐ *Neutral*<br>☐ *Uncomfortable*<br>☐ *Very uncomfortable*	*C2_S5_Q10a*
10b.	[If very uncomfortable; uncomfortable] Why were you uncomfortable with your peer helping you **use** your HIV self-test? [check all that apply]	☐ *I did not feel that my peer had the ability to help me use my HIVST*<br>☐ *My peer had trouble reading and interpreting the HIVST instructions*<br>☐ *I felt pressured to conduct the HIVST in the presence of my peer*<br>☐ *Prefer not to answer*<br>☐ *Other:___________________*	*C2_S5_Q10b*<br>*C2_S5_Q10b_other*
11.	[If Q3>0] Did your peer help you to **interpret** your HIV self-test results?	☐ *Yes*<br>☐ *No*	*C2_S5_Q11*
11a.	[If Q11== Yes] How comfortable were you with your peer helping you to **interpret** your HIV self-test results?	☐ *Very comfortable*<br>☐ *Comfortable*<br>☐ *Neutral*<br>☐ *Uncomfortable*<br>☐ *Very uncomfortable*	*C2_S5_Q11a*
11b.	[If very uncomfortable; uncomfortable] Why were you uncomfortable with your peer helping you **interpret**	☐ *I did not feel that my peer had the ability to help me interpret my HIVST*	*C2_S5_Q11b*<br>*C2_S5_Q11b_ot*

	your HIV self-test results? [check all that apply]	*results*<br>☐ *I feared disclosing an HIV positive status*<br>☐ *I feared disclosing an HIV negative status*<br>☐ *I felt pressured to conduct the HIVST in the presence of my peer*<br>☐ *I did not think my peer could help me link to care (PrEP/ HIV treatment)*<br>☐ *Prefer not to answer*<br>☐ *Other:___________________*	*her*
12.	[If Q3>0] Did you **share** (disclose) your HIV status with your peer?	☐ *Yes*<br>☐ *No [skip to Q14]*	*C2_S5_Q12*
12a.	[If Q12==Yes] How comfortable were you **sharing** (disclosing) your HIV status with your peer?	☐ *Very comfortable*<br>☐ *Comfortable*<br>☐ *Neutral*<br>☐ *Uncomfortable*<br>☐ *Very uncomfortable*	*C2_S5_Q12a*
12b.	[If very uncomfortable; uncomfortable] Why were you uncomfortable **sharing** (disclosing) your HIV status with your peer? [check all that apply]	☐ *I feared disclosing an HIV positive status*<br>☐ *I feared disclosing an HIV negative status*<br>☐ *I did not think my peer could help me link to care (PrEP/ HIV treatment)*<br>☐ *I do not think the HIVST results were correct*<br>☐ *I do not think my peer would keep my HIV status private*<br>☐ *Prefer not to answer*<br>☐ *Other:___________________*	*C2_S5_Q12b*<br>*C2_S5_Q12b_other*
13.	[If Q12==Yes] How confident are you that your peer kept your HIV status **private** (i.e., confidential)?	☐ *Very confident*<br>☐ *Fairly Confident*<br>☐ *Neutral*<br>☐ *A little confident*<br>☐ *Not at all confident*	*C2_S5_Q13*
14.	How beneficial do you think HIV self-testing is to you?<br>[TFA: Perceived Effectiveness]	☐ *Very beneficial*<br>☐ *Somewhat beneficial*<br>☐ *Neutral*<br>☐ *A little beneficial*<br>☐ *Not at all beneficial*	*C2_S5_Q14*
15.	In the future, would you be willing to pay for HIV self-tests?	☐ *Yes*<br>☐ *No*	*C2_S5_Q15*
15a.	[If Q15=Yes] How much would you be willing to pay for *HIV self-tests?*	|__|__||__|__||__|__||__|__|_|__*kSH*	*C2_S5_Q15a*

Now I am going to show you a series of pictures with different possible HIV self-test results and am going to ask you to interpret each result. Possible test results include: "HIV positive", "HIV negative" or "Invalid" (e.g., failed test).	*C2_S5_HIVST_interpret*

[These next four images will be presented to participants]

Source: HIVST Questionnaire (Ortblad)

Image Citation: Chembio Diagnostic Systems Inc. Sure Check HIV Self Test [Internet]. Medford, NY, United States: Chembio (2024). https://chembio.com/products/sure-check-hiv-self-test-international/. [Accessed August 13, 2024].

16.	How would you interpret the results of this self-test?<br>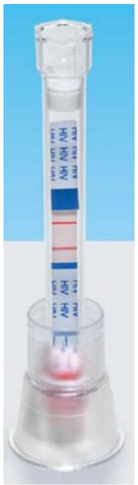	☐ *HIV-negative*<br>☐ *HIV-positive*<br>☐ *Invalid*<br>☐ *Don't know*	*C2_S5_Q16*
17.	How would you interpret the results of this self-test?<br>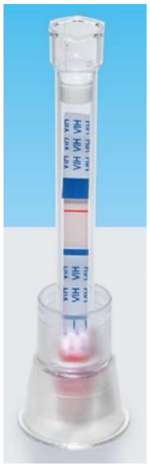	☐ *HIV-negative*<br>☐ *HIV-positive*<br>☐ *Invalid*<br>☐ *Don't know*	*C2_S5_Q17*
18.	How would you interpret the results of this self-test?<br>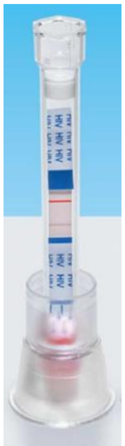	☐ *HIV-negative*<br>☐ *HIV-positive*<br>☐ *Invalid*<br>☐ *Don't know*	*C2_S5_Q18*

19.	How would you interpret the results of this self-test?<br>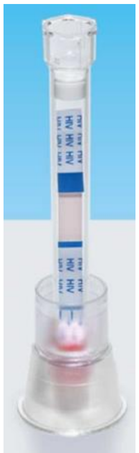	☐ *HIV-negative*<br>☐ *HIV-positive*<br>☐ *Invalid*<br>☐ *Don't know*	*C2_S5_Q19*
**5b. Feedback: PrEP**			*C2_S5_Prep*
20.	Did your peer **approach** and **talk** to you about PrEP?	☐ *Yes*<br>☐ *No [skip to Q22]*	*C2_S5_Q20*
20a.	How comfortable were you with your peer **approaching** you and **talking** to you about PrEP?	☐ *Very comfortable*<br>☐ *Comfortable*<br>☐ *Neutral*<br>☐ *Uncomfortable*<br>☐ *Very uncomfortable*	*C2_S5_Q20a*
20b.	[If very uncomfortable; uncomfortable] Why were you uncomfortable with your peer **approaching** and **talking** to you about PrEP? [check all that apply]	☐ *My peer did not appropriately approach me about PrEP*<br>☐ *I felt my peer accused me of being HIV positive*<br>☐ *I did not feel my peer had enough information/knowledge about PrEP*<br>☐ *I did not feel I had enough information/knowledge about PrEP*<br>☐ *Prefer not to answer*<br>☐ *Other:____________________*	*C2_S5_Q20b*<br>*C2_S5_Q20b_other*
21.	Did your peer **share** (disclose) with you if *they* take PrEP?	☐ *Yes*<br>☐ *No*	*C2_S5_Q21*
22.	Did your peer **link** you to PrEP services?	☐ *Yes*<br>☐ *No*	*C2_S5_Q22*
23.	Did your peer **accompany** you to a clinic to start PrEP?	☐ *Yes*<br>☐ *No*	*C2_S5_Q23*
24.	In the past month, did **you start taking** PrEP following this intervention?	☐ *Yes*<br>☐ *No [Skip to Q26]*	*C2_S5_Q24*
25.	In the past month, has your peer **supported** you take your PrEP medication every day?	☐ *Yes*<br>☐ *No*	*C2_S5_Q25*

25a.	[If Q25==Yes] How has your peer **supported** you to take your PrEP medication every day? [check all that apply]	☐ *By calling me*<br>☐ *By sending reminders/text messages*<br>☐ *By reminding me in person (i.e., at work or school)*<br>☐ *Other:____________________*	*C2_S5_Q25a*<br>*C3_S5_Q25a_other*
26.	How beneficial do you think PrEP is to you?<br><br>[TFA: Perceived Effectiveness]	☐ *Very beneficial*<br>☐ *Somewhat beneficial*<br>☐ *Neutral*<br>☐ *A little beneficial*<br>☐ *Not at all beneficial*	*C2_S5_Q26*
**5b. Feedback: Follow-up**			
27.	Did you ping study staff to participant in this research?	☐ *Yes*<br>☐ *No*	
28.	[If Q25==Yes] How difficult was this process?	☐ *Very difficult*<br>☐ *Difficult*<br>☐ *Neutral*<br>☐ *Easy*<br>☐ *Very easy*	
29.	[If Q25==No] Why did you not ping the study staff?	☐ *Peer provider didn't to tell them*<br>☐ *Didn't have a phone*<br>☐ *Didn't feel comfortable sharing phone number*<br>☐ *Didn't want to participate in research activities*<br>☐ *Didn't want others to know they were participating in the study*<br>☐ *PrEP stigma*<br>☐ *Other*	
30.	Do you have your own phone?	☐ *Yes*<br>☐ *No*	
31.	[If Q30==No] Is there a phone line that is not your own that you would feel comfortable having research staff call to contact you and discuss this intervention?	☐ *Yes*<br>☐ *No*	
**Section 6: PrEP use, perceptions, stigma**			*C2_S6_PrEP*
**6a. PrEP use and adherence**<br>*Now I would like to ask you some questions about your PrEP use and adherence to PrEP.*<br>*Source: Modified Medication Adherence Scale (Wilson et al., AIDS & Behavior, 2016)*			*C2_S6_PrEP_use_adherence*
1.	Are you currently taking PrEP?	☐ *Yes*<br>☐ *No*	*C2_S6_Q1*
2.	Were you currently taking PrEP when your peer referred you to PrEP?	☐ *Yes*<br>☐ *No*	*C2_S6_Q2*
3.	[If Q1==Yes] On which date did you start taking PrEP following peer referral?	*(Y Y Y Y) - (M M)- (DD)*	*C2_S6_Q3*
4.	[If Q1==Yes or Q2==Yes] After receiving PrEP medication, did you wait any days before starting to use	☐ *Yes*<br>☐ *No*	*C2_S6_Q4*

	PrEP?		
4a.	[If Q4= Yes] How many days did you wait to start using PrEP?	|__|__| *days*	*C2_S6_Q4a*
4b.	[If Q1==Yes or Q2==Yes] Have you stopped and re-started PrEP since then?	☐ *Yes*<br>☐ *No*	*C2_S6_Q4b*
5.	[If Q1==Yes or Q2==Yes] Have you stopped (i.e., discontinued) PrEP since initiation?	☐ *Yes*<br>☐ *No*	*C2_S6_Q5*
5a.	[If Q5=Yes] When did you stop (i.e., discontinue) using PrEP?	*(YYYY- MM- DD)*	*C2_S6_Q5a*
5b.	[If Q5 = Yes] Why have you stopped (i.e., discontinued) using PrEP? [check all that apply]	☐ *Experienced side effects*<br>☐ *Concerns about PrEP stigma*<br>☐ *No longer at HIV risk (e.g., relationship ended)*<br>☐ *Had a hard time using PrEP*<br>☐ *Had a hard time accessing PrEP*<br>☐ *Could not afford PrEP (e.g., transportation)*<br>☐ *Do not believe PrEP prevents HIV*<br>☐ *Prefer not to answer*<br>☐ *Other:_________________*	*C2_S6_Q5b*<br>*C2_S6_Q5b_other*
6.	[If Q1==Yes or Q2==Yes] Is this your first-time taking PrEP?	☐ *Yes*<br>☐ *No*	*C2_S6_Q6*
6a.	[If Q6 == No] On what date did you start taking PrEP the first time?	*(Y Y Y Y) - (M M)- (DD)*	*C2_S6_Q6a*
6b.	[If Q6==No] What are some of the reasons you stopped taking *PrEP* after you started the first time? [check all that apply]	☐ *Experienced side effects*<br>☐ *Concerns about PrEP stigma*<br>☐ *No longer at HIV risk (e.g., relationship ended)*<br>☐ *Had a hard time using PrEP*<br>☐ *Had a hard time accessing PrEP*<br>☐ *Could not afford PrEP (e.g., transportation)*<br>☐ *Did not believe PrEP prevented HIV*<br>☐ *Prefer not to answer*<br>☐ *Other:_________________*	*C2_S6_Q6b*<br>*C2_S6_Q6b_other*
7.	How did you first learn about PrEP?	☐ *Online/internet*<br>☐ *From healthcare workers/at a health facility*<br>☐ *Through **another** research study*<br>☐ *Through **this** research study*<br>☐ *Community outreach program*<br>☐ *Advertisements (posters, pamphlets, radio, etc.)*	*C2_S6_Q7*<br>*C2_S6_Q7_other*

		☐ *Friend/peer*<br>☐ *Family member*<br>☐ *Sexual partner(s)*<br>☐ *Other:_________________*	
8.	[If Q1==Yes or Q2==Yes] **In the past 30 days**, how many days did you miss taking your dose of PrEP?	|__|__| *days (number 0-30)*<br>☐ I have not yet initiated PrEP for 30 days	*C2_S6_Q8*
9.	[If Q1==Yes or Q2==Yes] **In the past 30 days**, how often did you take your PrEP in the way you were supposed to?	☐ *Never*<br>☐ *Rarely*<br>☐ *Sometimes*<br>☐ *Usually*<br>☐ *Almost always*<br>☐ *Always*	*C2_S6_Q9*
10.	[If Q1==Yes or Q2==Yes] **In the past 30 days**, how well do you think you took your PrEP in the way you were supposed to?	☐ *Very poor*<br>☐ *Poor*<br>☐ *Fair*<br>☐ *Good*<br>☐ *Very good*<br>☐ *Excellent*	*C2_S6_Q10*
11.	[If Q1==Yes or Q2==Yes] What strategies do you use to remember to take PrEP? [Check all that apply]	☐ *I do not use any strategies*<br>☐ *Alarm*<br>☐ *Reminders from peers*<br>☐ *Reminders from family members*<br>☐ *Reminders from sexual partner(s)*<br>☐ *Other: _______________*	*C2_S6_Q11*<br>*C2_S6_Q11_other*
12.	Do you know anyone taking PrEP?	☐ *Yes*<br>☐ *No*	*C2_S6_Q12*
12a.	[If Q12= Yes] Who do you know who is taking PrEP?	☐ *Sexual partner(s)*<br>☐ *Friend/peer(s)*<br>☐ *Family member(s)*<br>☐ *Other: _______________*	*C2_S6_Q12a*<br>*C2_S6_Q12a_other*
13.	[If Q1==Yes or Q2==Yes] Does anyone know you are taking PrEP?	☐ *Yes*<br>☐ *No*	*C2_S6_Q13*
13a	[If Q13== Yes] Who knows you are taking PrEP? [Check all that apply]	☐ *Sexual partner(s)*<br>☐ *Friend/peer(s)*<br>☐ *Family members*<br>☐ *Other: _______________*	*C2_S6_Q13a*<br>*C2_S6_Q13a_other*
14	[If Q1==Yes or Q2==Yes] Have you had any side effects after starting PrEP?	☐ *Yes*<br>☐ *No*	*C2_S6_Q14*
14a.	[If Q14== yes] Which side effects did you experience after starting PrEP? [Check all that apply]	☐ *Diarrhea*<br>☐ *Nausea*<br>☐ *Headache*<br>☐ *Stomachache*	*C2_S6_Q14a*<br>*C2_S6_Q14a_other*

		☐ *Fatigue*<br>☐ *Other:____________________*	
15	[If Q1==Yes or Q2==Yes] Thinking into the future, how much longer do you think you will continue using PrEP?	|__|__||__|__ *months*	*C2_S6_Q15*
16.	Would you be willing to pay for transportation to PrEP services in the future (i.e., PrEP refills)?	☐ *Yes*<br>☐ *No*<br>☐ *I am not eligible for PrEP (e.g., HIV-positive)*	*C2_S6_Q16*
16a.	[If Q16= Yes] How much would you be willing to pay for transportation to PrEP services (i.e., PrEP refills)?	|__|__||__|__||__|__||__|__|_|__*kSH*	*C2_S6_Q16a*
17.	*A new form of PrEP might be available soon, which is an injection (e.g., shot) you would have to take every 8 weeks.*<br><br>How interested would you be in this form of PrEP delivery?	☐ *Not at all interested*<br>☐ *Slightly interested*<br>☐ *Neutral*<br>☐ *Interested*<br>☐ *Very interested*	*C2_S6_Q17*
17a.	[If Q17= neutral, interested or very interested]: If this form of long-acting injectable PrEP were available now, what form of PrEP would you most prefer?	☐ *Long-acting injectable PrEP (with shots every 2 months)*<br>☐ *Daily oral PrEP (with refills every 3 months)*<br>☐ *Neither*<br>☐ *Don't know*	*C2_S6_Q17a*
17b.	[If Q17 = neutral, interested or very interested]: Where would you most prefer to access this long-acting injectable PrEP from?	☐ *Healthcare clinic*<br>☐ *Family planning clinic*<br>☐ *Retail pharmacy*<br>☐ *Other: _______________*	*C2_S6_Q17b*<br>*C2_S6_Q17b_other*
17c.	[If Q17 = neutral, interested or very interested]: How much would you be willing to pay for 1 shot of long-acting injectable PrEP (that provides 8 weeks, or 2 months, of protective coverage)?	|__|__||__|__||__|__||__|__|_|__*kSH*	*C2_S6_Q17c*
**6b. PrEP Perceptions**<br>*We will now ask you a few questions about your thoughts and feelings about daily (oral) PrEP.*<br>*Source: Modified scales from HIVST and Pharmacy PrEP questionnaires (Ortblad)*			*C2_S6_prep_perceptions_label*
18.	Do you think PrEP makes sex completely safe from HIV?	☐ *Yes*<br>☐ *No*<br>☐ *Unsure*	*C2_S6_Q18*
19.	How worried are you about getting HIV **in the next 3 months**?	☐ *Not worried*<br>☐ *Somewhat worried*<br>☐ *Very worried*<br>☐ *N/A I am currently living with HIV*	*C2_S6_Q19*
20.	Do you agree with the following statement: *"My future*	☐ *Completely agree*<br>☐ *Agree*	*C2_S6_Q20*

	health depends on me taking PrEP"?	☐ *Neutral*<br>☐ *Disagree*<br>☐ *Completely disagree*	
21.	**Before you began PrEP**, how worried were you about getting side effects from PrEP?	☐ *Not worried*<br>☐ *Somewhat worried*<br>☐ *Very worried*<br>☐ *I am not currently using PrEP*	*C2_S6_Q21*
**6c. PrEP Stigma**<br>*We are interested in understanding how you think people in your community generally feel about PrEP. I am going to read some statements, and I'd like you to indicate how much you agree or disagree with the statement. Your answer choices are completely agree; agree; disagree; or completely disagree; or don't know.*<br>*SOURCE: Modified perceived stigma (Kaai, Sahara-J: Journal of Social Aspects of HIV/AIDS, 2012).*			*C2_S6_prep_stigma_label*
22.	**The first statement is,** *"People in my community think people who take PrEP are promiscuous and having casual sex."*	☐ *Completely agree*<br>☐ *Agree*<br>☐ *Neutral*<br>☐ *Disagree*<br>☐ *Completely disagree*<br>☐ *Don't know*	*C2_S6_Q22*
23.	**The second statement is,** *"People in my community think people who take PrEP are being responsible for their sexual health."*	☐ *Completely agree*<br>☐ *Agree*<br>☐ *Neutral*<br>☐ *Disagree*<br>☐ *Completely disagree*<br>☐ *Don't know*	*C2_S6_Q23*
24.	**The third statement is**, *"People in my community think PrEP may not be safe for your health."*	☐ *Completely agree*<br>☐ *Agree*<br>☐ *Neutral*<br>☐ *Disagree*<br>☐ *Completely disagree*<br>☐ *Don't know*	*C2_S6_Q24*
25.	**The last statement is,** *"People in my community think that taking PrEP means you have HIV."*	☐ *Completely agree*<br>☐ *Agree*<br>☐ *Neutral*<br>☐ *Disagree*<br>☐ *Completely disagree*<br>☐ *Don't know*	*C2_S6_Q25*
**Section 7. Acceptability, Appropriateness, Feasibility of a Peer PrEP Referral + HIVST model**<br>*Now we are going to ask you some questions about how acceptable you found the intervention.*<br>*Source: Modified Theoretical Framework of Acceptability (TFA) adapted from the Pharmacy PrEP study (Ortblad & Roche).*			*C2_S7_acceptability_appropriateness_feasibility*
I'm going to read a series of statements, and I would like you to say how much you agree or disagree with each statement. Your choices are "completely disagree", "disagree", "neutral", "agree", "completely agree"			

**7a. Acceptability of a peer PrEP referral + HIVST model**			*C2_S7_acceptability*
1.	**The first statement is:** *"I liked receiving HIVST kits from my peer and being referred to PrEP (or HIV treatment)."*<br><br>[TFA: Affective Attitude]	☐ *Completely disagree*<br>☐ *Disagree*<br>☐ *Neutral*<br>☐ *Agree*<br>☐ *Completely agree*	*C2_S7_Q1*
2.	**The second statement is:** *"I found it hard to receive HIVST kits from my peer and be referred to PrEP (or HIV treatment). "*<br><br>[TFA: Burden]	☐ *Completely disagree*<br>☐ *Disagree*<br>☐ *Neutral*<br>☐ *Agree*<br>☐ *Completely agree*	*C2_S7_Q2*
3.	**The third statement is:** *"I was confident in my ability to carry-out all elements of the intervention successfully (i.e., use HIVST kits and enroll in PrEP/HIV treatment)."*<br>[TFA: Self-Efficacy]	☐ *Completely disagree*<br>☐ *Disagree*<br>☐ *Neutral*<br>☐ *Agree*<br>☐ *Completely agree*	*C2_S7_Q3*
4.	**The fourth statement is:** *"I was confident in my ability to solve any problems that arose while receiving this intervention (e.g., use HIVST kits and enroll in PrEP/HIV treatment)."*<br><br>[TFA: Self-Efficacy]	☐ *Completely disagree*<br>☐ *Disagree*<br>☐ *Neutral*<br>☐ *Agree*<br>☐ *Completely agree*	*C2_S7_Q4*
5.	**The fifth statement is:** *"It was acceptable for me to receive HIVST kits from my peer and be referred to PrEP (or HIV treatment)."*<br><br>[General Acceptability]	☐ *Completely disagree*<br>☐ *Disagree*<br>☐ *Neutral*<br>☐ *Agree*<br>☐ *Completely agree*	*C2_S7_Q5*
6.	**The sixth statement is: "***I didn't understand why I should receive HIVST kits from my peer and be referred to PrEP (or HIV treatment)"*<br><br>[TFA: Intervention coherence]	☐ *Completely disagree*<br>☐ *Disagree*<br>☐ *Neutral*<br>☐ *Agree*<br>☐ *Completely agree*	*C2_S7_Q6*
7.	**The seventh statement is***: "It is clear to me how this intervention helps to reduce HIV among young women in my community"*<br><br>[TFA: Intervention coherence]	☐ *Completely disagree*<br>☐ *Disagree*<br>☐ *Neutral*<br>☐ *Agree*<br>☐ *Completely agree*	*C2_S7_Q7*
8.	**The eight statement is:** *"Receiving HIVST kits from my peer and being referred to PrEP (or HIV treatment) interfered with my other priorities"*<br>[TFA: Opportunity Costs]	☐ *Completely disagree*<br>☐ *Disagree*<br>☐ *Neutral*<br>☐ *Agree*	*C2_S7_Q8*

		☐ *Completely agree*	
9.	**The ninth statement is:** *"Receiving HIVST kits from my peer and being referred to PrEP (or HIV treatment) did not create any moral or ethical consequences for me"*<br>[TFA: Ethicality]	☐ *Completely disagree*<br>☐ *Disagree*<br>☐ *Neutral*<br>☐ *Agree*<br>☐ *Completely agree*	*C2_S7_Q9*
10.	**The tenth statement is:** "*Receiving HIVST kits from peers and being referred to PrEP helps young women to remain HIV-negative"*<br>[TFA: Perceived effectiveness]	☐ *Completely disagree*<br>☐ *Disagree*<br>☐ *Neutral*<br>☐ *Agree*<br>☐ *Completely agree*	*C2_S7_Q10*
**7b. Appropriateness of a peer PrEP referral + HIVST model**			*C2_S7_appropriateness*
11.	This intervention (i.e., HIVST delivery with peer PrEP referral) seems fitting to adolescent girls and young women.	☐ *Completely disagree*<br>☐ *Disagree*<br>☐ *Neutral*<br>☐ *Agree*<br>☐ *Completely agree*	*C2_S7_Q11*
12.	This intervention (i.e., HIVST delivery with peer PrEP referral) seems like a good match to HIV prevention programs in Kenya.	☐ *Completely disagree*<br>☐ *Disagree*<br>☐ *Neutral*<br>☐ *Agree*<br>☐ *Completely agree*	*C2_S7_Q12*
**7c. Feasibility of a peer PrEP referral + HIVST model**			*C2_S7_feasibility*
13.	This peer delivered intervention (i.e., HIVST delivery and peer PrEP referral) seems possible to implement in Kenya.	☐ *Completely disagree*<br>☐ *Disagree*<br>☐ *Neutral*<br>☐ *Agree*<br>☐ *Completely agree*	*C2_S7_Q13*
14.	This intervention seems easy to implement in Kenya.	☐ *Completely disagree*<br>☐ *Disagree*<br>☐ *Neutral*<br>☐ *Agree*<br>☐ *Completely agree*	*C2_S7_Q14*
**Section 8. Acceptability of Peer-delivered PrEP**<br>*In the future, PrEP may be delivered by peers in the community. We would like to ask you a few questions about how acceptable you would find delivering or receiving PrEP from a peer. Please remember that we are now asking about peer delivered PrEP and not a peer referral to PrEP.*<br>*Source: Modified Theoretical Framework of Acceptability (TFA) adapted from the Pharmacy PrEP study (Ortblad & Roche).*			*C2_S8_acceptability*
1.	**The first statement is:** *"I think I would like delivering PrEP directly to my peers"*	☐ *Completely disagree*<br>☐ *Disagree*<br>☐ *Neutral*	*C2_S8_Q1*

	[TFA: Affective Attitude]	☐ *Agree*<br>☐ *Completely Agree*	
2	**The second statement is:** *"I think I would like receiving PrEP directly from my peers"*<br>[TFA: Affective Attitude]	☐ *Completely disagree*<br>☐ *Disagree*<br>☐ *Neutral*<br>☐ *Agree*<br>☐ *Completely Agree*	*C2_S8_Q2*
3.	**The third statement is:** *"I think it would be hard to receive PrEP directly from my peers"*<br>[TFA: Burden]	☐ *Completely disagree*<br>☐ *Disagree*<br>☐ *Neutral*<br>☐ *Agree*<br>☐ *Completely Agree*	*C2_S8_Q3*
4.	**The fourth statement is:** *"I am confident that I would be able to receive PrEP directly from my peers"*<br>[TFA: Self-Efficacy]	☐ *Completely disagree*<br>☐ *Disagree*<br>☐ *Neutral*<br>☐ *Agree*<br>☐ *Completely Agree*	*C2_S8_Q4*
5.	**The fifth statement is:** *"It is clear to me how the PrEP delivered by peers can reduce HIV among young women in my community"*<br>[TFA: Intervention coherence]	☐ *Completely disagree*<br>☐ *Disagree*<br>☐ *Neutral*<br>☐ *Agree*<br>☐ *Completely Agree*	*C2_S8_Q5*
6.	**The sixth statement is:** *"Receiving PrEP directly from my peers would likely interfere with my other priorities"*<br>[TFA: Opportunity Costs]	☐ *Completely disagree*<br>☐ *Disagree*<br>☐ *Neutral*<br>☐ *Agree*<br>☐ *Completely Agree*	*C2_S8_Q6*
7.	**The seventh statement is:** *"Receiving PrEP directly from my peers would not create any moral or ethical consequences for me"*<br>[TFA: Ethicality]	☐ *Completely disagree*<br>☐ *Disagree*<br>☐ *Neutral*<br>☐ *Agree*<br>☐ *Completely Agree*	*C2_S8_Q7*
8.	**The eight statement is:** "*Receiving PrEP directly from peers would help young women to remain HIV-negative"*<br>[TFA: Perceived effectiveness]	☐ *Completely disagree*<br>☐ *Disagree*<br>☐ *Neutral*<br>☐ *Agree*<br>☐ *Completely Agree*	*C2_S8_Q8*

**Section 9. Human Papillomavirus (HPV)**<br>*Now I would like to ask you some questions about HPV.*<br>*Source: Modified Gaborone Children's Health Survey (DiAngi et al., PLoS One, 2011)*<br>*Source: Modified Cervical Cancer and Awareness Screening (Mingo et al., International Journal of Gynecological Cancer, 2012)*			*C2_S9_HPV*
1.	Have you ever heard of **Human Papillomavirus** or **HPV**? *(HPV is different from HIV).*	☐ *Yes*<br>☐ *No*	*C2_S9_Q1*
2.	Have you heard of genital warts?	☐ *Yes*<br>☐ *No*	*C2_S9_Q2*
2a.	[if Q1==Yes or if Q2==Yes] Has a doctor or other medical professional ever told you that you have genital warts or HPV?	☐ *Yes*<br>☐ *No*<br>☐ *Prefer not to answer*	*C2_S9_Q2a*
3.	Have you ever heard of cancer of the cervix or cervical cancer?	☐ *Yes*<br>☐ *No*	*C2_S9_Q3*
3a.	[if Q3==Yes] Has a doctor or other medical professional ever told you that you have cervical cancer?	☐ *Yes*<br>☐ *No*<br>☐ *Prefer not to answer*	*C2_S9_Q3a*
3b.	[if Q3==Yes] Do you know anyone who has been diagnosed or treated for cervical cancer?	☐ *Yes*<br>☐ *No*<br>☐ *Prefer not to answer*	*C2_S9_Q3b*
3c.	[If Q3==Yes] Who do you know that has been diagnosed or treated for cervical cancer? [check all that apply]	☐ *Family member*<br>☐ *Friend/peer*<br>☐ *Neighbor/community member*<br>☐ *Other:______________________*	*C2_S9_Q3c*<br>*C2_S9_Q3c_other*
An HPV vaccine is now available that protects against most genital warts and cervical cancer. Sometimes it's called the cervical cancer vaccine, HPV shot, Cervavix, or Gardasil.			*C2_S9_HPV_vaccine*
4.	Have you heard of the HPV vaccine before today?	☐ *Yes*<br>☐ *No*	*C2_S9_Q4*
5.	[if Q4==Yes] Have you received the HPV vaccine (any or all doses)?	☐ *Yes*<br>☐ *No*<br>☐ *Unsure*	*C2_S9_Q5*
5a.	[If Q4==No; If Q5= No; Unsure] Would you be interested in getting an HPV vaccine in the future?	☐ *Yes*<br>☐ *No*<br>☐ *Unsure*	*C2_S9_Q5a*
5b.	[If Q5a==No; Unsure]: Why would you not be interested in getting the HPV vaccine? [check all that apply]	☐ *Don't know enough about the HPV vaccine*<br>☐ *Don't feel at risk of HPV*<br>☐ *Worried about side effects*<br>☐ *Concerns about HPV vaccine safety*<br>☐ *Concerns of stigma associated with the HPV vaccine*<br>☐ *Other:_________________*	C2_S9_Q5b<br>C2_S9_Q5b_other
6.	[If Q5==Yes | Q5a==Yes]: If the HPV vaccine were available at all of the following locations, where would	☐ *Public clinic*<br>☐ *Private clinic*	*C2_S9_Q6*<br>*C2_S9_Q6_other*

	you most prefer to access the HPV vaccine?	☐ *Retail pharmacy*<br>☐ *Online delivery*<br>☐ *I would not like an HPV vaccine*<br>☐ *Other:________*	
7.	[If Q5==Yes | Q5a==Yes]: How comfortable would you feel receiving your HPV vaccine at your local retail pharmacy?	☐ *Very uncomfortable*<br>☐ *Uncomfortable*<br>☐ *Neutral*<br>☐ *Comfortable*<br>☐ *Very comfortable*	*C2_S9_Q7*
8.	[If Q5==Yes | Q5a==Yes]: How much might you be willing to pay to receive 1 dose of the HPV vaccine at your local retail pharmacy?	|__|__||__|__||__|__||__|__|_|__*kSH*	*C2_S9_Q8*
**Section 3 EX: Extended Depression Screening (PHQ 9)**<br>*[This portion of the questionnaire will only appear for individuals who reported depression in the depression screening. Please collect the following information in a culturally appropriate way in the context of a counseling session and be prepared to offer referral to mental health services. This PHQ 9 will only include the 7 questions not previously asked in this questionnaire]*<br>*Source: PHQ 9 (Kroenke & Spitzer, 2002)*			*C2_S3_EX*
3.	**In the past two weeks,** how often have you had trouble falling asleep, staying asleep, or sleeping too much?	☐ *Not at all (0 days)*<br>☐ *Several days (1-7 days)*<br>☐ *More than half the days (8-12 days)*<br>☐ *Nearly every day (13-14 days)*	*C2_S3_Q3*
4.	**In the past two weeks,** how often have you felt tired or had little energy?	☐ *Not at all (0 days)*<br>☐ *Several days (1-7 days)*<br>☐ *More than half the days (8-12 days)*<br>☐ *Nearly every day (13-14 days)*	*C2_S3_Q4*
5.	**In the past two weeks,** how often have you been bothered by poor appetite or overeating?	☐ *Not at all (0 days)*<br>☐ *Several days (1-7 days)*<br>☐ *More than half the days (8-12 days)*<br>☐ *Nearly every day (13-14 days)*	*C2_S3_Q5*
6.	**In the past two weeks,** how often have you been feeling bad about yourself or that you're a failure and have let yourself or your family down?	☐ *Not at all (0 days)*<br>☐ *Several days (1-7 days)*<br>☐ *More than half the days (8-12 days)*<br>☐ *Nearly every day (13-14 days)*	*C2_S3_Q6*

7.	**In the past two weeks,** how often have you had trouble concentrating on things, such as reading a newspaper or watching television?	☐ *Not at all (0 days)*<br>☐ *Several days (1-7 days)*<br>☐ *More than half the days (8-12 days)*<br>☐ *Nearly every day (13-14 days)*	*C2_S3_Q7*
8.	**In the past two weeks,** how often have you been moving so slowly that other people could have noticed. Or the opposite- being so fidgety and restless that you have been moving around a lot more than usual?	☐ *Not at all (0 days)*<br>☐ *Several days (1-7 days)*<br>☐ *More than half the days (8-12 days)*<br>☐ *Nearly every day (13-14 days)*	*C2_S3_Q8*
9.	**In the past two weeks,** how often have you been bothered by thoughts that you would be better off dead or hurting yourself in some way?	☐ *Not at all (0 days)*<br>☐ *Several days (1-7 days)*<br>☐ *More than half the days (8-12 days)*<br>☐ *Nearly every day (13-14 days)*	*C2_S3_Q9*
**SHR: Social harm report**<br>*[This portion of the questionnaire will only appear for individuals who reported any social harm above. Please collect the following information in a culturally appropriate way in the context of a counseling session and please be prepared to offer referral to GBV/IPV services if warranted.]*			*C2_SHR_social_harms*
1.	In the **last 3 months**, was the participant **verbally** abused by anyone?	☐ *Yes*<br>☐ *No*<br>☐ *Prefer not to answer*	*C2_SHR_Q1*
1a.	[If Q1='Yes'] How often was the participant **verbally** abused (in the last 3 months)?	|__|__|__| *number of times*<br>☐ *Don't know*<br>☐ *Prefer not to answer*	*C2_SHR_Q1a*
1b.	[If Q1='Yes'] Who was the participant **verbally** abused by? [check all that apply]	☐ *Family member*<br>☐ *Intimate partner(s)*<br>☐ *Neighbor/ community member*<br>☐ *Friend/peer*<br>☐ *Other:_____________*<br>☐ *Prefer not to answer*	*C2_SHR_Q1b*<br>*C2_SHR_Q1b_other*
1c.	[If Q1='Yes'] Was this **verbal** abuse a consequence of this study/study activities?	☐ *Yes*<br>☐ *No*<br>☐ *Prefer not to answer*	*C2_SHR_Q1c*
1d	If Q1c='Yes'] Please concisely describe the **verbal abuse** resulting from this study/study activities:	☐ *Prefer not to answer*<br><br>*Explain: ____________________*	*C2_SHR_Q1d*

2.	In the **last 3 months**, was the participant **physically** abused by anyone	☐ *Yes*<br>☐ *No*<br>☐ *Prefer not to answer*	*C2_SHR_Q2*
2a.	[If Q2='Yes'] How often was the participant **physically** abused (in the last 3 months)?	|__|__|__| *number of times*<br>☐ *Don't know*<br>☐ *Prefer not to answer*	*C2_SHR_Q2a*
2b	[If Q2='Yes'] Who was the participant **physically** abused by? [check all that apply]	☐ *Family member*<br>☐ *Intimate partner(s)*<br>☐ *Neighbor/ community member*<br>☐ *Friend/peer*<br>☐ *Other:_____________*<br>☐ *Prefer not to answer*	*C2_SHR_Q2b*<br>*C2_SHR_Q2b_other*
2c	[If Q2='Yes'] Was this **physical** abuse a consequence of this study/study activities?	☐ *Yes*<br>☐ *No*<br>☐ *Prefer not to answer*	*C2_SHR_Q2c*
2d	[If Q2c=Yes] Please concisely describe the **physical abuse** resulting from this study/study activities:	☐ *Prefer not to answer*<br><br>*Explain: ____________________*	*C2_SHR_Q2d*
3.	In the **last 3 months**, was the participant **emotionally** abused by anyone?	☐ *Yes*<br>☐ *No*<br>☐ *Prefer not to answer*	*C2_SHR_Q3*
3a.	[If Q3='Yes'] How often was the participant **emotionally abused** (in the last 3 months)?	|__|__|__| *number of times*<br>☐ *Don't know*<br>☐ *Prefer not to answer*	*C2_SHR_Q3a*
3b	[If Q3='Yes'] Who was the participant **emotionally** abused by? [check all that apply]	☐ *Family member*<br>☐ *Intimate partner(s)*<br>☐ *Neighbor/ community member*<br>☐ *Friend/peer*<br>☐ *Other:_____________*<br>☐ *Prefer not to answer*	*C2_SHR_Q3b*<br>*C2_SHR_Q3b_other*
3c	[If Q3='Yes'] Was this **emotional** abuse a consequence of this study/study activities?	☐ *Yes*<br>☐ *No*<br>☐ *Prefer not to answer*	*C2_SHR_Q3c*
3d	[If Q3c= Yes] Please concisely describe the **emotional** abuse resulting from this study/study activities:	☐ *Prefer not to answer*<br><br>*Explain: ____________________*	*C2_SHR_Q3d*
4	Was the participant abused by anyone in any other way (not fitting into the categories of verbal, physical, or emotional abuse)?	☐ *Yes (If Yes, describe in Q4a)*<br>☐ *No*<br>☐ *Prefer not to answer*	*C2_SHR_Q4*
4a	Please concisely describe the social harm:	☐ *Prefer not to answer*<br><br>*Explain: ____________________*	*C2_SHR_Q4a*

4b	[If Q4='Yes'] How often did this social harm occur in the last three months?	|__|__|__| *number of times*<br>☐ *Don't know*<br>☐ *Prefer not to answer*	*C2_SHR_Q4b*
4c	[If Q4=='Yes'] Who did the participant experience this social harm from? [check all that apply]	☐ *Family member*<br>☐ *Intimate partner(s)*<br>☐ *Neighbor/ community member*<br>☐ *Friend/peer*<br>☐ *Other:_____________*<br>☐ *Prefer not to answer*	*C2_SHR_Q4c*<br>*C2_SHR_Q4c_other*
4d	Was this social harm a consequence of the study/study activities?	☐ *Yes*<br>☐ *No*<br>☐ *Prefer not to answer*	*C2_SHR_Q4d*
