# Supplementary material for: Formalized peer referral to HIV pre-exposure prophylaxis supported with self-testing: a mixed-methods pilot study among young Kenyan women
Source: Front Public Health. 2024 Sep 11;12:1428609. doi: 10.3389/fpubh.2024.1428609 (PMC11422135; doi:10.3389/fpubh.2024.1428609)
Supplement: Supplementary file 2 [file Data_Sheet_2.pdf]

IRO REC'D 7/12/2022

Peer PrEP: Focus Group Discussion Guide - Peer Provider

<u>Interviewer instructions</u>: Please find a quiet/private sitting area where all participants can be seated and given the opportunity to take part in the Focus Group Discussion.

Main Research Question: What are weak points of the peer PrEP referral + HIVST delivery model that could potentially be refined?

**Interview Information**<br><br>***The focus group discussions will include approximately 6 participants.***<br><br>***Please record the following information prior to moving onto the Focus Group Discussion. During the interview, please keep in mind that participants should refer to each other by number (1-6) rather than using their names.***
(a) Participant IDs:________________<br>*Format: R1, R2, R3….*<br><br>(b) Informed consent has been administered: YES / NO<br>*If consent form has not been signed by participant, interview must not proceed.*<br><br>(c) Interview ID: _____________________________<br>*Format: (Interview type-interview number-participant type)*<br>*Ex: FGD-01-peer provider*<br><br>(d) Date of interview: ____/_____/_______<br>*Format: DD/MM/YYYY*<br><br>(e) Location of interview: ____<br><br>(f) Interviewer's full name: ____<br><br>(g) Interview start time: ____________________<br>*Format: HH:MM am <u>or</u> pm*<br><br>(h) Interview end time: _____________________<br>*Format: HH:MM am <u>or</u> pm*

<u>Facilitator introduction</u>: [DO NOT READ; GUIDE ONLY]

FHCRC IRB Approval
AUG 11 2022
Document Released Date

Hello. My name is _______, and I am a ________, working at _______. Thank you for taking the time to have a discussion with me today.

The purpose of this discussion group is to understand your experiences delivering HIV self-test kits to your friends and referring them to PrEP (or HIV treatment) services. We are specifically interested to learn what you found easy or challenging and how we can improve on this approach in the future.

I'm going to pose some open-ended questions to the group, and I'd like all of you to share your thoughts and experiences both in response to my questions but also in response to things that your fellow participants say. A lot of the discussion today will actually be among each other rather than directly with me. My role is to encourage the conversation and make sure that everyone has a chance to participate. So please, feel welcome to interact with each other directly.

This discussion should take around an hour. Please let me know if at any time you have questions, if something I say is not clear, or if anyone needs to take a break.

Demographics (to be completed with each participant individually):	
1. Participant age:	|__|__| *number of years*
2. Where do you live? *(city or village names)*	______________________________
3. Are you currently using PrEP?	☐ *Yes*<br>☐ *No*
4. Have you ever used an HIV self-test to test yourself for HIV?	☐ *Yes, blood-based HIV self-test only*<br>☐ *Yes, oral-fluid HIVST only*<br>☐ *Yes, both blood-based and oral-fluid based HIVST*<br>☐ *No*<br>☐ *Prefer not to answer*
5. After the training, how many friends did you approach to talk to about HIV self-testing and PrEP?	|__|__| *number of friends*
6. How many of your friends accepted the HIV self-testing kits from you?	|__|__| *number of friends*
7. To the best of your knowledge, how many of your friends started PrEP after you provided them with HIV self-testing kits?	|__|__| *number of friends*<br>☐ *Prefer not to answer*
8. To the best of your knowledge, how many of your friends started HIV treatment after you provided them with HIV self-testing kits?	|__|__| *number of friends*<br>☐ *Prefer not to answer*
9. To what extent did you like being a "peer provider"- giving HIV self-testing kits to your friends and encouraging them to start PrEP (or HIV treatment)?	☐ *Strongly dislike*<br>☐ *Dislike*<br>☐ *Neutral*<br>☐ *Like*<br>☐ *Strongly like*

10. To what extent would you like to be a “peer provider” in the future?	☐ *Strongly dislike*<br>☐ *Dislike*<br>☐ *Neutral*<br>☐ *Like*<br>☐ *Strongly like*

Research question we are trying to answer with this FGD: X

1. *All of you received training on how to HIV self-test, how to give HIV self-testing kits to your friends, and how to help them use the HIV self-test if they needed support.* ***We'd like to know: what did you think about this part of the training?***

   PROBES*: Were there any things you liked about the training you received on HIV self-testing? Were there any things you found confusing? Did your friends ask you questions about HIV self-testing that you felt like you could not answer? What could we do to make this part of the training better?*

2. *In the training you also learned about PrEP and how to encourage your friends to enroll in PrEP at a health facility of their choosing if they tested HIV-negative.* ***We'd like to know: what did you think about this part of the training?***

   PROBES*: Were there any things you liked about the training you received on PrEP? Were there any things you found confusing? Did your friends ask you questions about PrEP that you felt like you could not answer? What could we do to make this part of the training better?*

3. *As a peer provider, you talked to your friends about HIV self-testing and PrEP.* ***How difficult or easy was it to engage your friends in these conversations?***

   PROBES*: [If participants indicate it was easy:]*

   Can you tell me about how you did it, such as where you were when you brought up these topics and what you said to get the conversation going? What recommendations would you have for other peer providers who want to engage their friends in such conversations?

   [If participants indicate that it was difficult, probe to understand what, exactly, made it difficult—for example, whether it was difficult to find a private place to have the conversation or to get the friend alone; whether their friends are really private/shy people and seemed uncomfortable with the subject matter, etc.]

   Can you tell me more about why it was difficult to have these conversations with your friends? What recommendations would you have for other peer providers who want to engage their friends in such conversations?

4. *After you talked to your friends about HIV self-testing and PrEP, some friends may have not wanted to use the HIV self-testing kits or be referred to PrEP.* ***We'd like to know: why do you think these friends may not have wanted to use the HIV self-testing kits or be referred to PrEP?***

   PROBES: *[if no participants had peers refuse to join the study, this question may also be asked hypothetically. For example, we can probe to find out the barriers some young women may face when presented with this intervention]. What do you think may prevent young women from accepting HIV self-testing kits and being referred*

to PrEP? What recommendations would you propose to encourage these young women to accept HIV self-testing kits or PrEP referrals?

5. *As a peer provider, you also gave HIV self-tests to your friends and may have described how to use the kit.* ***What was that experience like for you?***

   PROBES*: Did your friends have any concerns about the HIV self-test or challenges using the HIV self-test? If so, what, if anything, did you do in those scenarios? [Probe to understand what they told their friends, whether they ultimately helped their friend conduct the test, and/or if they used any other tactics to support their friends to use the HIV self-test, like advising their friend to watch an instructional video online.] What recommendations would you have for other peer providers who want to deliver HIV self-tests to their friends?*

6. *Some of you may have had friends share their HIV self-test result with you, and you encouraged these friends to consider going to a clinic of their choosing for either PrEP or ART.* ***We'd like to know: after talking to your friends about going to a clinic, how easy or difficult was it for you to convince them to go?***

   PROBES*: Were there any things that you did to help your friend make it to the clinic? [Probe to understand, for example, whether they texted their friend the clinic address, helped their friend book an appointment, accompanied their friend to the clinic, etc.] For your friends who did not ultimately go a clinic for PrEP or ART, why do you think they didn't go? [Probe to understand if it was because the friend wasn't ready/interested in starting PREP/ART, if they faced other barriers like transportation or stigma, etc.] What recommendations would you have for other peer providers who want to connect their friends to PrEP or ART?*

7. ***What are some of the things you like about being a peer provider?***

   PROBES*: In what ways, if any, did being a peer provider impact your life for the better? What did you like about being a peer provider?*

8. ***What are some of the things you don't like about being a peer provider?***

   PROBES*: What were some negative consequences of being a peer provider? What did you dislike about being a peer provider?*

9. *In the future, we would like other young women, like yourself, to serve as peer providers.* ***What might encourage other young women to give HIV self-testing kits to their friends and refer their friends to PrEP or ART services?***

   PROBES: *Beyond financial compensation, what motivated you to be a peer provider for this study? Do you think other young women may be similarly motivated to be peer providers, why or why not? What can you recommend would encourage other young women to become peer providers to their communities?*

Research question we are trying to answer with this FGD: X

10. *Aside from the things we've already discussed today,* ***what would you change about this intervention?***<br><br>*We have come to the end of our discussion. What else would you like to share with me today?*

We have come to the conclusion of the topics I had prepared to discuss today. THANK YOU FOR YOUR TIME!

[Mark interview end time on page 1 (Item I).]

Focus Group Discussion Guide- Peer Client

Interviewer instructions: Please find a quiet/private sitting area where all participants can be seated and given the opportunity to take part in the Focus Group Discussion.

Main Research Question: What are weak points of the peer PrEP referral + HIVST delivery model that could potentially be refined?

**Interview Information**
The focus group discussions will include approximately 6 participants.   ***Please record the following information prior to moving onto the Focus Group Discussion. During the interview, please keep in mind that participants should refer to each other by number (1-6) rather than using their names.***
1. Participant IDs:________________   *Format: R1, R2, R3….* <br>2. Informed consent has been administered: YES / NO   *If consent form has not been signed by participant, interview must not proceed.* <br>3. Interview ID: _____________________________   *Format: (Interview type-interview number-participant type)*   *Ex: FGD-01-peer clients* <br>4. Date of interview: ____/_____/_______   *Format: DD/MM/YYYY* <br>5. Location of interview: ____ <br>6. Interviewer's full name: ____ <br>7. Interview start time: ____________________   *Format: HH:MM am or pm* <br>8. Interview end time: _____________________   *Format: HH:MM am or pm*

FHCRC IRB Approval
AUG 11 2022
Document Released Date

<u>Facilitator introduction</u>: [DO NOT READ; GUIDE ONLY]

Hello. My name is _______, and I am a ________, working at _______. Thank you for taking the time to have a discussion with me today.

The purpose of this discussion group is to understand your experiences receiving HIV self-test kits from your friends and being referred to PrEP or HIV treatment services. We are specifically interested to learn what you found easy or challenging and how we can improve on this approach in the future.

I'm going to pose some open-ended questions to the group, and I'd like all of you to share your thoughts and experiences both in response to my questions but also in response to things that your fellow participants say. A lot of the discussion today will actually be among each other rather than directly with me. My role is to encourage the conversation and make sure that everyone has a chance to participate. So please, feel welcome to interact with each other directly.

This discussion should take around an hour. Please let me know if at any time you have questions, if something I say is not clear, or if anyone needs to take a break.

Demographics (to be completed with each participant individually):	
1. Participant age:	|__|__| *number of years*
2. Where do you live? *(city or village names)*	____
3. Have you ever used an HIV self-test to test yourself for HIV?	☐ *Yes, blood-based HIV self-test only*<br>☐ *Yes, oral-fluid HIVST only*<br>☐ *Yes, both blood-based and oral-fluid based HIVST*<br>☐ *No*<br>☐ *Prefer not to answer*
4. Are you currently using PrEP?	☐ *Yes*<br>☐ *No*
5. [If Q4=No] Are you currently using HIV treatment?	☐ *Yes*<br>☐ *No*
6. To what extent did you like being given an HIV self-testing kit and being referred to PrEP/ HIV treatment by your friend?	☐ *Strongly dislike*<br>☐ *Dislike*<br>☐ *Neutral*<br>☐ *Like*<br>☐ *Strongly like*
7. How knowledgeable did you find your friend on HIV self-testing and PrEP?	☐ *Not at all knowledgeable*<br>☐ *A little knowledgeable*<br>☐ *Neutral*<br>☐ *Knowledgeable*<br>☐ *Very knowledgeable*
8. In the future, you may have the opportunity to take part in the training provided to your friend and be a "peer provider"- give HIV self-test kits to friends and refer them to PrEP or HIV treatment. To what extent would *you* like to be a peer provider?	☐ *Strongly dislike*<br>☐ *Dislike*<br>☐ *Neutral*<br>☐ *Like*<br>☐ *Strongly like*

Questions
1. *The friends who gave you HIV self-test kits and referred you to PrEP or HIV treatment, were provided a one-day training where they learned how to use HIV self-test kits and how to support you to HIV self-test, if necessary.* ***We'd like to know: how informed was your friend about HIV self-testing?***<br><br>***PROBES****: What did your friend teach you about HIV self-testing? Was your peer able to answer all of your questions about HIV self-testing? What would you have liked to learn about HIV self-testing from your friend, if at all? How confident do you feel about your friend's knowledge of HIV self-testing?*<br><br>2. *In the training given to your friend, they also learned about PrEP and how to encourage their friends, all of you, to enroll in PrEP if the results of the HIV self-test are HIV negative.* ***We'd like to know: how informed was your peer about PrEP?***<br><br>***PROBES****: What did your friend teach you about PrEP? Was your friend able to answer all of your questions about PrEP? What would you have liked to learn about PrEP from your friend, if at all? How confident do you feel about your friend's PrEP knowledge?*<br><br>3. *After your friend approached you about HIV self-testing and PrEP/HIV treatment:* ***How difficult or easy was it to have these conversations with your friends?***<br><br>***PROBES****: [If participants indicate it was easy:]*<br>*Can you tell me about how your friend approached you about these topics, such as where you were and what they said? How did you react to your friend approaching you about these topics? What did you like about being approached by your friend about these topics? What recommendations would you have for young women who approach friends about HIV self-testing and PrEP/HIV treatment in the future?*<br><br>*[If participants indicate that it was difficult, probe to understand what, exactly, made it difficult—for example, whether they found it inappropriate to be approached and talked to about HIV self-testing or PrEP/HIV treatment, whether the location where one was approached was not private; or how these conversations were approached etc.]*<br>*Can you tell me more about why it was difficult for you to have these conversations with your friend? How would you have liked to be approached and talked to about these topics by your friend? What recommendations would you have for young women who approach friends about HIV self-testing and PrEP/HIV treatment in the future?*<br><br>***4.*** *You should have received HIV self-testing kits from your friend. In some cases, your friend may have described how to use the kit or shown you how to use the kit.* ***What was that experience like for you?***<br><br>***PROBES****: Did you have any concerns about the HIV self-test or challenges using the HIV self-test? If so, what, if anything, did your friend do to help you? [Probe to understand if the peer provider helped the client conduct the test, and/or provided them with other tactics to support their HIVST use, like advising their friend to watch an instructional*

video online.] What recommendations would you have to improve how HIV self-tests were delivered to you? What can be improved on to support you to HIV self-test?

5. *Some of you may have shared the results of your HIV self-test with your friend. Please do not share the results of your HIV self-test with the group.* ***We would like to know: if your friend supported you to go to a clinic (for PrEP or HIV treatment services), and if so, how they supported you?***

   PROBES*: Were there any things your friend did to help you make it to the clinic? [Probe to understand, for example, whether their friend texted them the clinic address, if their friend helped them book an appointment, if their friend accompanied them to the clinic, etc.] What recommendations would you have to support other young women, like you, to go to PrEP or HIV treatment services?*

6. ***What are some of the things you like*** *about being given HIV self-testing kits and referred to PrEP/HIV treatment?*

   PROBES*: In what ways, if any, has this impacted your life for the better? What did you like about being given HIV self-testing kits and being referred to PrEP/HIV treatment?*

7. ***What are some of the things you don't like*** *about being given HIV self-testing kits and referred to PrEP/HIV treatment?*

   PROBES*: What were some negative consequences about this? What did you dislike about being given HIV self-testing kits and referred to PrEP/HIV treatment?*

8. *Beyond financial compensation for this study,* ***what motivated you to accept HIV self-testing (and possibly be referred to PrEP or HIV treatment)?***

   PROBES: *Do you think other young women may be similarly motivated to accept HIV self-testing and enroll in PrEP/or HIV treatment, why or why not? What can you recommend would encourage other young women to accept HIV self-testing and enroll in PrEP/ HIV treatment?*

9. *Aside from the things we've already discussed today,* ***what would you change about this intervention?***

We have come to the end of our discussion. What else you would like to share with me today?

We have come to the conclusion of the topics I had prepared to discuss today. THANK YOU FOR YOUR TIME!

[Mark interview end time on page 1 (Item I).]
